# A heat-induced HagF-HrcA regulatory loop promotes chlamydial fitness and infectious progeny yield under stress

**DOI:** 10.1371/journal.ppat.1014749

**Published:** 2026-11-04

**Authors:** Yehong Huang, Yuxuan Wang, Matthew Pan, Danny Wan, Lingling Wang, Joseph D. Fondell, Xiang Wu, Guangming Zhong, Huizhou Fan

**Affiliations:** 1 Department of Pediatrics, Xiangya Hospital, Central South University, Changsha, Hunan, China; 2 Department of Pharmacology, Robert Wood Johnson Medical School, Rutgers University, Piscataway, New Jersey, United States of America; 3 Department of Parasitology, Central South University Xiangya Medical School, Changsha, Hunan, China; 4 Department of Microbiology and Immunology, University of Texas Health San Antonio, San Antonio, Texas, United States of America; Max Planck Institute for Terrestrial Microbiology: Max-Planck-Institut fur terrestrische Mikrobiologie, GERMANY

## Abstract

Bacterial survival under environmental stress requires precise regulation of protein homeostasis, yet how this is achieved in organisms with streamlined genomes remains incompletely understood. Here, we identify HagF as a previously unrecognized protein that antagonizes the conserved heat-inducible transcriptional repressor HrcA in *Chlamydia*. Using genetic and biochemical approaches, including overexpression and CRISPRi-mediated knockdown, we show that under non-stressed conditions, HrcA represses *hagF* along with its five known targets, including *hrcA* and four chaperone genes. Upon heat stress, HagF is induced and binds HrcA, attenuating its repressor activity. This negative feedback loop enhances chaperone gene induction, particularly from the *hrcA-grpE-dnaK* operon, and promotes infectious elementary body yield. Notably, HagF is conserved across members of the order *Chlamydiales*, highlighting a lineage-specific adaptation. Together, these findings define a regulatory circuit in which a specialized antagonist modulates a widely conserved repressor, enabling *Chlamydia* to couple heat-responsive gene expression with developmental progression and enhance fitness under restrictive conditions.

## Author summary

Pathogens must adapt to stressful conditions to survive and spread, yet how bacteria with very small genomes accomplish this remains unclear. *Chlamydia*, a common human pathogen, encounters thermal and other host-associated stresses during infection and must preserve its developmental capacity to remain infectious. In this study, we identify a previously uncharacterized protein, HagF, that helps *Chlamydia* respond to heat stress. We show that HagF counteracts the activity of HrcA, a conserved protein that normally represses stress-response genes. By inhibiting HrcA, HagF enhances molecular chaperone expression and infectious progeny yield under stress conditions. This interaction forms a feedback loop that fine-tunes gene expression during stress. Our findings reveal how a pathogen with a streamlined genome uses a specialized regulatory protein to control a conserved stress-response system, thereby enabling it to maintain developmental fitness in challenging host environments.

## Introduction

The ability of living cells to respond to environmental stress is fundamental to survival, with the heat shock response being among the most studied mechanisms of cellular defense [1–5]. This universally conserved response equips organisms to withstand and recover from the detrimental effects of elevated temperatures and other stressors through transcriptomic reprogramming [1,3–10]. In many bacteria, the upregulation of stress-responsive genes is mediated by specialized sigma factors that target RNA polymerase to distinct heat shock promoters [1,3–5]. Additionally, some stress-response genes can be upregulated following dissociation of heat-inducible transcriptional repressors from their target promoters [1,3–5,11]. In this regard, HrcA is the most widely conserved transcriptional repressor among bacteria.

Members of the phylum Chlamydiota, including the human pathogen *Chlamydia trachomatis*, are obligate intracellular bacteria with a distinctive developmental cycle that alternates between the infectious elementary body (EB) and the intracellular, replicative reticulate body (RB) [12–14]. Under extreme stress, RBs cease division but may continue to replicate their genomes, resulting in enlarged, non-dividing forms known as aberrant bodies. This condition, termed chlamydial persistence, is reversible upon return to favorable conditions, when aberrant bodies convert back to dividing RBs and resume progeny EB formation [7,8,15–18]. In response to milder stress, some RBs may enter persistence, whereas others continue to replicate slowly, resulting in reduced but detectable infectious progeny yield [19,20]. This distinction is particularly relevant for thermal stress, which can vary in magnitude and duration in host tissues and may impair, rather than completely arrest, the developmental cycle. Unlike stresses that impose specific nutrient limitation, antimicrobial damage, or strong immune restriction, moderate thermal stress may primarily challenge protein homeostasis, making chaperone-mediated maintenance of development particularly important. The ability to preserve or recover infectious progeny yield under such restrictive conditions is important for maintaining chlamydial fitness and developmental competence [6,7,16,18,21,22].

Despite possessing one of the smallest bacterial genomes and lacking a dedicated heat shock sigma factor, *Chlamydia* is capable of mounting a robust heat shock response characterized by extensive transcriptomic reprogramming [5,10]. This suggests expanded roles for other transcriptional regulators. Indeed, heat-induced expression changes in numerous chlamydial genes have been linked to alterations in the expression and activities of the remaining sigma factors (i.e., σ^66^, σ^28^, and σ^54^) [5], thought to primarily control chlamydial growth and development.

HrcA is the only known heat-inducible transcriptional repressor in *Chlamydia*. Similar to numerous other bacteria [23–26], HrcA directly regulates two operons in *C. trachomatis* that encode five canonical genes: *hrcA*, *grpE*, *dnaK*, *groES*, and *groEL* [27,28]. Under non-stressed conditions, HrcA binds CIRCE (controlling inverted repeat of chaperone expression) elements in the *dnaK* promoter (KP1) and *groESL* promoter (EP) [23–28]. Binding to KP1 represses *hrcA*, *grpE*, and *dnaK*, which encode components of the DnaK-GrpE chaperone system. Binding to EP represses *groES* and *groEL*, whose products form the *GroES-groEL* (GroESL) chaperonin complex. These systems assist in the folding of nascent polypeptides and the refolding or stabilization of stress-denatured proteins to maintain proteostasis under both physiological and stress conditions [29,30]. During heat stress, HrcA becomes destabilized, relieving repression and enabling induction of both chaperone systems to meet increased demand [23–28].

The repression by HrcA under non-stress conditions is thought to be reinforced by GroEL- or GroESL-mediated stabilization of HrcA [31–33], forming a positive feedback loop. Here, we identify *ctl0271* as a sixth HrcA target gene. This gene, now termed *hagF* (heat-activated HrcA-repressed gene F), encodes a lineage-specific protein conserved among Chlamydiota. We show that *hagF* is strongly induced by heat shock and that its product binds HrcA and interferes with its association with CIRCE DNA. This establishes a heat-induced negative feedback loop that complements GroESL-mediated positive feedback. By amplifying chaperone gene induction, especially from the *dnaK-grpE* operon, HagF supports late developmental gene expression and promotes infectious progeny yield. These findings define a feedback architecture that fine-tunes the chaperone response and enhances chlamydial fitness in restrictive host environments.

## Results

### *ctl0271 (hagF)* is a novel heat-activated gene repressed by HrcA

HrcA is known to repress the expression of five chlamydial genes by binding to the promoters of the *hrcA-grpE-dnaK* and *groES-groEL* operons under non-stressed conditions (Fig 1A), with repression relieved during heat stress (Fig 1B) [27,28]. We recently identified *ctl0271* as a novel gene strongly upregulated by heat shock [5], and suspected it may be an additional HrcA regulatory target because its promoter region contains a putative CIRCE element (Figs 1C and S1). The *ctl0271* CIRCE element is located five nucleotides upstream of a predicted -35 core promoter element positioned relative to a transcription start site previously mapped by deep-sequencing-based transcriptome analysis (S1 Fig), the same positioning as the CIRCE elements found in the promoters of the *hrcA-grpE-dnaK* and *groES-groEL* operons, KP1 and EP (Fig 1A), respectively. Notably, the sequence of the *ctl0271* CIRCE element deviates from that of the consensus bacterial CIRCE element by only two nucleotides [34], one more than the KP1 CIRCE element but two fewer than the EP element (Fig 1C). Although the KP1 and EP CIRCE elements both form stronger stem regions near the nine-nucleotide loop, the *ctl0271* CIRCE element possesses both higher sequence conservation and a more stable stem region when compared to the bona fide CIRCE element found in *Sinorhizobium* (formerly *Rhizobium*) *meliloti* [35] (Fig 1C). These findings suggest that, in addition to the *hrcA-grpE-dnaK* and *groES-groEL* operons, chlamydial HrcA also regulates the expression of *ctl0271.*

**Fig 1:**
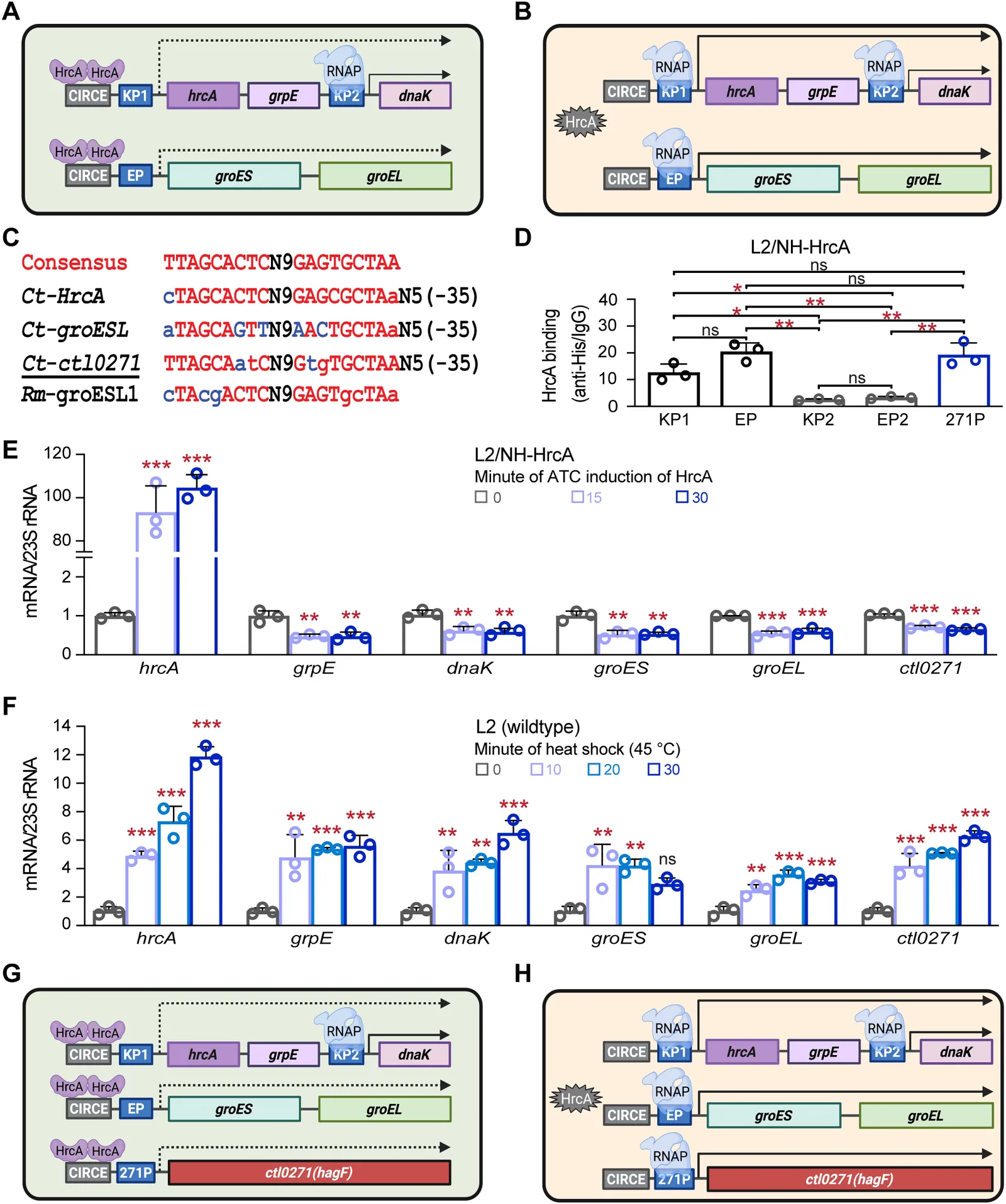
*ctl0271 (hagF)* is the 6th heat-inducible HrcA-repressed gene. (A) Under non-stressed conditions, HrcA binds to CIRCE elements and represses transcription from the KP1 and EP promoters. (B) Heat shock disrupts HrcA repression, allowing RNA polymerase (RNAP) to transcribe the *hrcA-grpE-dnaK* and *groES-groEL* operons. Arrows denote the extent of transcription. Note that *dnaK* is regulated by both the operon promoter and its own internal promoter (KP2). (C) Consensus and putative CIRCE sequences upstream of indicated heat shock genes in *C. trachomatis (Ct)* and *Rhizobium meliloti (Rm)*. N5 denotes the five-nucleotide spacer separating the inverted repeat arms of the CIRCE element, while N9 denotes the nine nucleotides between the CIRCE element and the downstream -35 core promoter element. Blue letters indicate nucleotide deviations from the consensus; lowercase letters denote mismatches between inverted repeats. The -35 core promoter elements for *C. trachomatis* genes are shown. (D) HrcA binds the *ctl0271* promoter in chlamydiae. ChIP-qPCR was performed in biological triplicate using L2/NH-HrcA cultures treated with 10 nM ATC for 30 min at 37 °C. Anti-His antibody enrichment of the *ctl0271* promoter was comparable to that of known HrcA targets (*groESL* and *dnaK* P1), and significantly higher than enrichment at non-target promoters KP2 and EP2 (*groEL2* promoter) [31,36]. (E) Expression of *ctl0271*, *dnaK*, and *groEL* was reduced upon ATC-induced NH-HrcA overexpression in L2/NH-HrcA, as determined by RT-qPCR. (F) *ctl0271* expression is induced in wild-type L2 cultured at 45 °C, as measured by RT-qPCR. (D–F) Data represent the mean ± SD of biological triplicates. (G, H) Based on results from panels B–F, the updated HrcA regulon includes *ctl0271* (renamed *hagF*) as its sixth target gene. (A, B, G, H) Created in BioRender. Wang, Y. (2026), https://BioRender.com/mk82ltj.

To establish whether HrcA binds to the *ctl0271* promoter in *C. trachomatis*, we conducted chromatin immunoprecipitation followed by quantitative PCR (ChIP-qPCR) in *C. trachomatis* L2 (L2) transformed with a plasmid expressing His-tagged HrcA (L2/NH-HrcA). As expected, anti-His immunoprecipitation detected enriched HrcA binding at the KP1 and EP promoters by 12.6- and 20.4-fold over control IgG, respectively (Fig 1D). Significantly, the anti-His antibody also revealed a 19.1-fold enrichment in HrcA binding at the *ctl0271* promoter (denoted 271P) (Fig 1D). These results clearly indicate that HrcA binds at the *ctl0271* promoter in *C. trachomatis* L2.

Based on both the presence of a CIRCE element (Fig 1C) and HrcA binding at its promoter (Fig 1D), and given the functional role of HrcA as a transcriptional repressor, we hypothesized that HrcA represses *ctl0271* expression. To test this hypothesis, we first performed reverse transcription-real-time PCR (RT-qPCR) analysis in L2/NH-HrcA chlamydiae. We found that induction of NH-HrcA expression at 37 ºC for 15 and 30 min resulted in statistically significant decreases in *ctl0271* transcripts (-1.4 and -1.5-fold, respectively) (Fig 1E), compared with control non-induction. These decreases are comparable to decreases in *grpE, dnaK, groES,* and *groEL* expression following NH-HrcA induction (Fig 1E)*.* We next determined the effect of heat shock in cultures of wildtype L2 cells. We used 45 °C as a short-term experimental heat-shock induction condition, rather than as a model of physiological fever, to robustly activate the chlamydial heat-shock response through heat-induced relief of HrcA-mediated repression [5]. This acute condition has also been used previously to examine HrcA-dependent heat-shock transcription in infected cells [36]. Incubation at 45 °C for 10, 20, and 30 minutes resulted in 4.2-, 5.1-, and 6.3-fold increases in *ctl0271* expression, respectively, compared to incubation at 37 °C (Fig 1F). Importantly, these increases are comparable to increases in *hrcA, grpE, dnaK, groES,* and *groEL* expression following heat induction (Fig 1F)*.* The marked expression increase in response to heat shock, and modest decreased expression in response to massive *hrcA* overexpression, is consistent with the notion that like the KP1 and EP promoters, 271P is also repressed by HrcA in chlamydiae cultured at 37 °C and strongly de-repressed in response to heat shock.

Taken together, our findings reveal *ctl0271* to be a novel heat-activated regulatory target gene of HrcA in *C. trachomatis* (Fig 1G and 1H)*.* We have therefore renamed *ctl0271* as *hagF* (heat-activated HrcA-regulated gene F). Notably, HagF is conserved in all *Chlamydia* species and *Chlamydia-*like organisms (S2 Fig). Although BlastP analysis using *C. trachomatis* HagF as a query revealed that one non-chlamydia bacterium, *Bacteroides fragilis*, might express a HagF-like ortholog (S3A Fig), further analysis showed that the predicted *B. fragilis* protein sequence is identical to that of the HagF ortholog in *C. gallinacea* (S3B Fig), implying that the *B. fragilis* DNA in that sequence analysis was likely contaminated with *C. gallinacea* genomic DNA. The sequence data thus reveal HagF as a Chlamydiales-specific protein, suggesting that it evolved to play an important role in chlamydial physiology.

### HagF promotes infectious progeny yield under heat stress

In addition to regulating stress responses, some HrcA target genes are known to be essential for bacterial growth under non-stress conditions [37,38]. To probe the physiological role of HagF, we employed deactivated CRISPR-associated protein 12 (dC12) [39,40] to silence *hagF* expression in *C. trachomatis*. We derived three L2 transformants, each expressing dC12 in an ATC-inducible manner and constitutively expressing a dC12 guide RNA (gRNA). While gRNA g1 and g2 target different regions of *hagF,* ntg targets a synthetic gene not found in *Chlamydia* (see Materials and Methods). As expected, in the transformant L2/dC12-ntg, induction of dC12 expression with ATC did not affect the expression level of *hagF* (Fig 2A). In comparison, in transformants L2/dC12-hagF*-*g1 and L2/dC12-hagF*-*g2, induction of dC12 expression (Fig 2A) resulted in -5.4 and -3.1 fold changes, respectively, in the level of *hagF* transcripts (Fig 2B), thus demonstrating the effective *hagF* silencing by both gRNAs.

**Fig 2:**
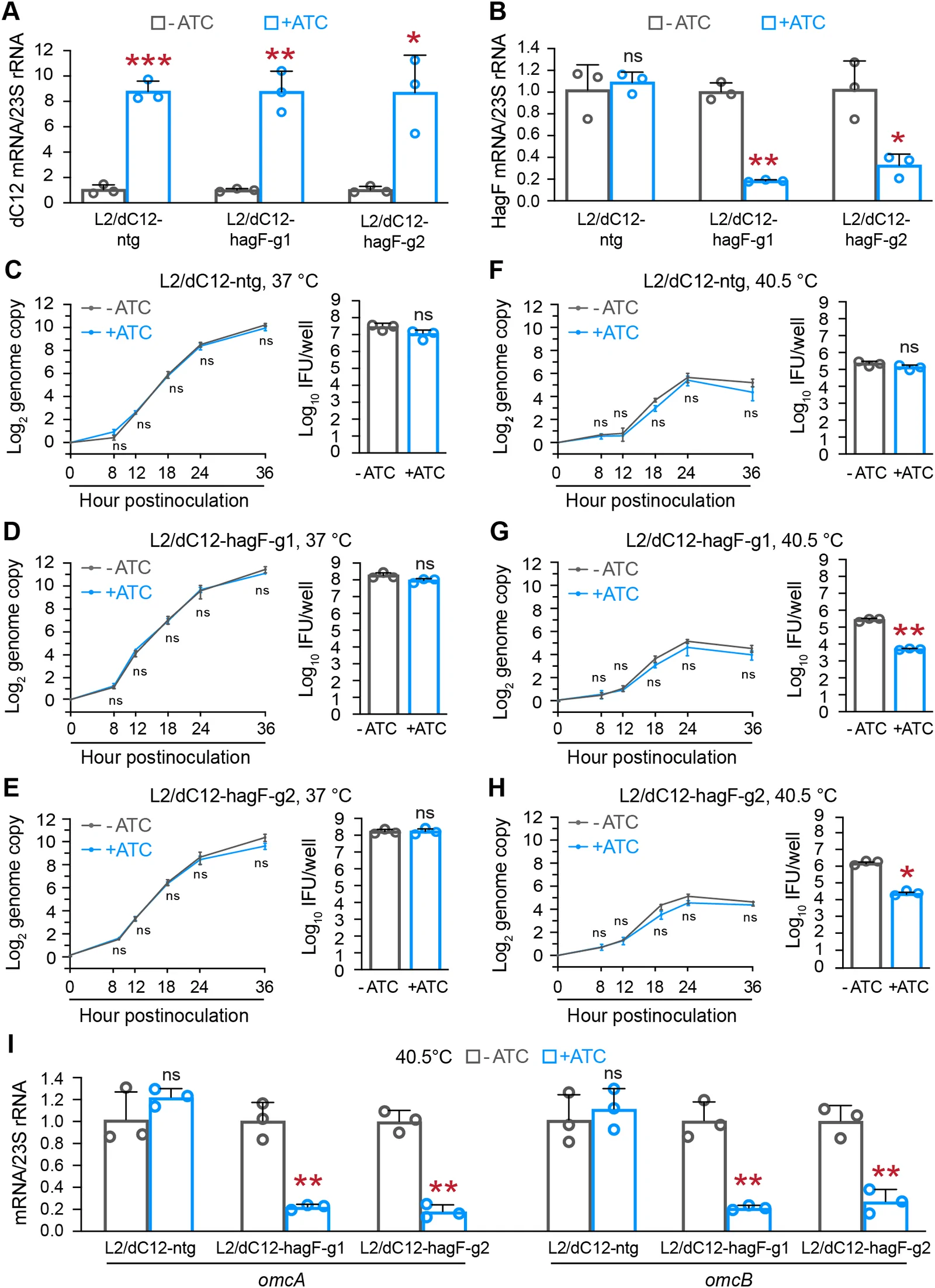
*hagF* facilitates infectious progeny yield during heat shock. (A) ATC-induced increases in the transcripts of deactivated CRISPR-associated protein 12 (dC12) in cultures of L2/dC12-ntg, L2/dC12-hagF-g1, and L2/dC12-hagF-g2, *C. trachomatis* L2 transformants of a vector carrying an ATC-inducible dC12 together with a non-targeting guide RNA (ntg) or the *hagF*-targeting guide RNA g1 or g2. (B) Effective silencing of *hagF* in L2/dC12-hagF-g1 and L2/dC12-hagF-g2 but not L2/dC12-ntg. (A, B) RNA levels were quantified by RT-qPCR and normalized to 23S rRNA (23S). (C) ATC-induced *dC12* expression has no effect on genome replication (left) or infectious progeny yield (right) in 37 °C cultures of L2/dC12-ntg. (D, E) ATC-induced *hagF* silencing has no detectable effect on genome replication (left) or infectious progeny yield (right) in 37 °C cultures of L2/dC12-hagF-g1 (D) or L2/dC12-hagF-g2 (E). (F) ATC-induced *dC12* expression has no effect on genome replication (left) or infectious progeny yield (right) in 40.5 °C cultures of L2/dC12-ntg. (G, H) ATC-induced *hagF* silencing does not affect genome replication (left) but reduces the inclusion-forming units (right) in 40.5 °C cultures of L2/dC12-hagF-g1 (G) and L2/dC12-hagF-g2 (H). (C-H) ATC was added at 0 hpi. Genome copy number was quantified at the indicated times. IFU yields were measured at 30 hpi for 37°C cultures and at 40 hpi for 40.5°C cultures to account for slower chlamydial developmental kinetics at elevated temperature. (I) *hagF* knockdown reduces expression of late EB-associated genes during heat stress. Transcript levels of *omcA* and *omcB* were measured by RT-qPCR in L2/dC12-ntg, L2/dC12-hagF-g1, and L2/dC12-hagF-g2 cultured in the absence or presence of ATC.

When dC12 expression was ATC-induced in L2/dC12-ntg chlamydiae cultured at 37 °C, the genome replication kinetics as well as EB yield were indistinguishable from uninduced bacteria (Fig 2C). These findings show that dC12 has no significant effect on chlamydial RB replication or progeny EB yield. Similarly, when L2/dC12-hagF-g1 and L2/dC12-hagF-g2 were cultured at 37 °C, ATC-induced *hagF* silencing through g1 or g2 did not affect the genome replication kinetics or EB yield (Fig 2D and 2E), indicating that *hagF* is not essential for chlamydial growth and development under normal culture conditions.

We next assessed the effects of HagF silencing on chlamydial growth and development. We first performed a temperature titration to identify heat-stress conditions that impaired, but did not completely arrest, chlamydial development. Infectious progeny yields progressively declined as culture temperature increased from 39 to 40.5 °C (S4 Fig), whereas no detectable IFUs were recovered from any strain at 41 °C, with or without ATC treatment. *hagF* knockdown reduced infectious progeny yield throughout the 39–40.5 °C range, with the strongest effect observed at 40.5 °C (S4 Fig). We therefore used 40.5 °C to examine the effects of HagF silencing on chlamydial genome replication and infectious progeny yield in greater detail (Fig 2F–2H). Similar to 37 °C cultures, ATC-induced dC12 expression in L2/dC12-ntg did not affect genome replication or infectious progeny yield at 40.5 °C (Fig 2F). By contrast, ATC-induced *hagF* silencing in L2/dC12-hagF-g1 and L2/dC12-hagF-g2 did not significantly affect genome replication kinetics but markedly reduced recovered inclusion-forming units (IFUs) at 40.5 °C (Fig 2G and 2H).

To determine whether impaired late developmental progression contributes to the reduced IFU yield, we measured expression of the late, EB-enriched genes *omcA* and *omcB* at 40.5 °C. ATC treatment did not reduce *omcA* or *omcB* transcript levels in the control strain L2/dC12-ntg, whereas *hagF* silencing significantly decreased expression of both genes in L2/dC12-hagF-g1 and L2/dC12-hagF-g2 (Fig 2I). Together with the IFU and genome replication data, these results indicate that impaired late developmental progression contributes to this phenotype and support a physiological role for HagF in promoting infectious progeny yield during heat stress.

### HagF interacts with HrcA and interferes with its ability to bind CIRCE DNA

To understand how HagF promotes infectious progeny EB yield under heat stress, we first examined its subcellular localization, which has been controversial in the literature [41–43]. We employed an ATC-inducible system to express HagF carrying either an N- or C-terminal His tag (NH-HagF and CH-HagF) to minimize potential interference with protein trafficking by the tag [44–50]. Following ATC induction for 6 h, both NH-HagF (S5A Fig) and CH-HagF (S5B Fig) displayed granular signals that overlapped with those of GrgA, a known chlamydial cytoplasmic transcription factor [51,52]. These observations support the presence of HagF within chlamydiae and are consistent with substantial cytoplasmic localization, although they do not exclude the possibility that a fraction of HagF may also associate with the bacterial surface.

Previous studies demonstrated that the bacterial chaperonin GroEL interacts with HrcA to enhance its transcriptional repression activity [31]. To determine whether HagF might similarly interact with HrcA and influence its regulatory function, we performed pulldown assays using purified HagF and HrcA proteins carrying differential affinity tags. His-tagged HagF efficiently pulled down Strep-tagged HrcA (Fig 3A). Conversely, His-tagged HrcA also pulled down Strep-tagged HagF (Fig 3B). These reciprocal pulldown experiments support a direct interaction between HagF and HrcA.

**Fig 3:**
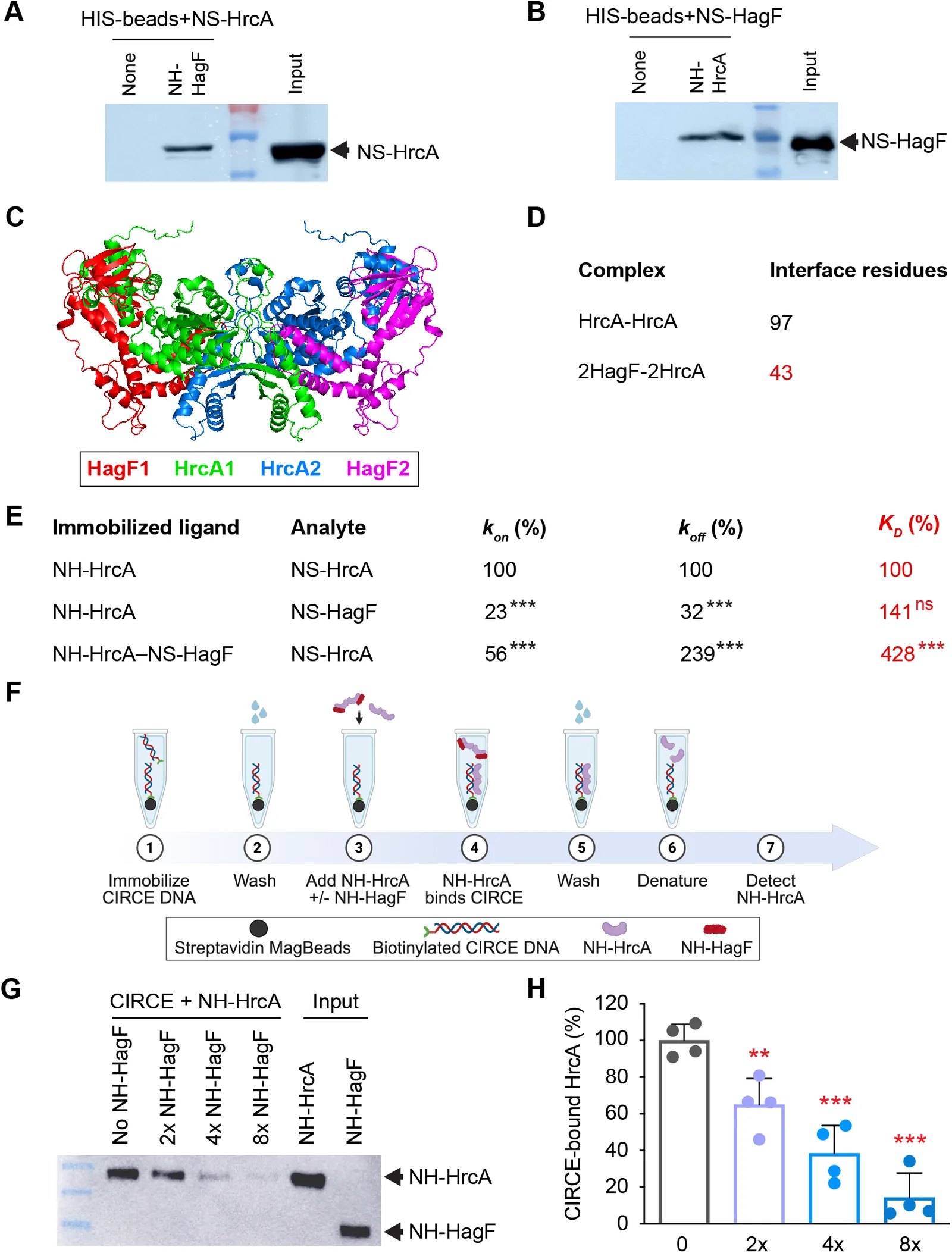
HagF binds HrcA and inhibits its association with CIRCE DNA. (A, B) HagF physically interacts with HrcA. In panel A, purified Strep-tagged HrcA (NS-HrcA) was incubated with purified His-tagged HagF (NH-HagF) and subjected to pulldown using His affinity beads. In panel B, purified Strep-tagged HagF (NS-HagF) was incubated with purified His-tagged HrcA (NH-HrcA) and analyzed using the same pulldown strategy. After washing, bead-bound proteins were resolved by SDS-PAGE and detected using Strep-Tactin HRP conjugate. Input lanes show the corresponding proteins prior to pulldown. (C) AlphaFold3-predicted 2HagF–2HrcA heterotetramer. HagF1, HrcA1, HrcA2, and HagF2 are shown in red, green, blue, and magenta, respectively. (D) Comparison of HrcA residues participating in the predicted HrcA–HrcA interface in the HrcA homodimer and 2HagF–2HrcA heterotetramer models. The 2HagF–2HrcA heterotetramer retains a reduced HrcA–HrcA interface. Detailed interface residues are listed in S1 Table. (E) Relative biolayer interferometry (BLI) kinetic parameters for HrcA–HrcA, HrcA–HagF, and HrcA–HagF/HrcA interactions. Values are expressed relative to the NH-HrcA versus NS-HrcA interaction, which was set to 100%. NH-HrcA–NS-HagF denotes a preformed HrcA–HagF complex immobilized on the biosensor. Full kinetic constants are provided in S2 Table, and representative sensorgrams are shown in S8 Fig. (F) Schematic of the CIRCE DNA-binding assay. Biotinylated CIRCE DNA was immobilized on streptavidin magnetic beads and incubated with NH-HrcA in the absence or presence of increasing NH-HagF. Created in BioRender. Wang, Y. (2026) https://BioRender.com/uc9ne1x. (G) Representative western blot showing reduced CIRCE-bound HrcA with increasing NH-HagF. (H) Quantification of CIRCE-bound HrcA. Data represent mean ± SD from four independent experiments. (E, H) **P < 0.01; ***P < 0.001.

To investigate a possible mechanism underlying this interaction, we used AlphaFold3 [53] to model chlamydial HrcA and HagF, first individually and then in combination. As a reference point for the HrcA model, the predicted structure of *C. trachomatis* HrcA closely resembled the experimentally determined structure of HrcA from *Thermotoga maritima* (S6 Fig) [54]. We next examined the predicted HagF models. When modeled alone, HagF adopted a less compact and less ordered conformation (S7A Fig). By contrast, HagF appeared more structurally ordered when modeled as part of an HrcA-containing complex (S7B Fig), suggesting that HrcA binding may stabilize HagF conformation.

AlphaFold3 further predicted that HagF and HrcA can form a 2HagF–2HrcA heterotetramer (Fig 3C). In this model, the HagF-containing complex retained an HrcA–HrcA interface but with a reduced number of predicted interface residues compared with the HrcA homodimer model (Fig 3D and S1 Table). Specifically, 97 HrcA residues were predicted to participate in the HrcA–HrcA interface in the HrcA homodimer model, whereas 43 HrcA residues were predicted to participate in the HrcA–HrcA interface in the 2HagF–2HrcA model (Fig 3D). These modeling results generate the hypothesis that HagF binding may weaken, rather than completely disrupt, HrcA self-association, but do not by themselves establish the stoichiometry or molecular interface of an authentic complex.

To test this possibility experimentally, we performed biolayer interferometry (BLI) assays using NH-HrcA as the immobilized ligand and NS-HrcA or NS-HagF as analytes. HrcA self-associated with HrcA and also bound HagF *in vitro* (Fig 3E and S2 Table). Relative to the NH-HrcA/NS-HrcA interaction, NH-HrcA bound NS-HagF with a lower apparent association rate, a lower apparent dissociation rate, and a comparable apparent affinity, with relative *k*_on_, *k*_off_, and *K*_D_ values of 24%, 32%, and 139%, respectively (Fig 3E and S2 Table). We next examined whether HagF affects the ability of HrcA to bind additional HrcA. When NH-HrcA was preincubated with NS-HagF before immobilization, the resulting NH-HrcA–NS-HagF complex retained the ability to bind NS-HrcA but did so with reduced apparent affinity. Compared with the NH-HrcA/NS-HrcA interaction, the NH-HrcA–NS-HagF/NS-HrcA interaction showed a reduced relative *k*_on_ of 56%, an increased relative *k*_off_ of 234%, and an increased relative *K*_D_ of 430% (Fig 3E and S2 Table). These data indicate that HagF weakens HrcA self-association, consistent with the reduced HrcA–HrcA interface predicted in the 2HagF–2HrcA model.

To directly test whether HagF affects HrcA binding to CIRCE DNA, we performed an *in vitro* DNA-binding assay using purified proteins. A biotinylated fragment containing the CIRCE element of the KP1 promoter was immobilized on streptavidin beads and incubated with recombinant NH-HrcA in the absence or presence of increasing amounts of NH-HagF (Fig 3F). As expected, HrcA robustly associated with the CIRCE element in the absence of HagF (Fig 3G). Addition of HagF resulted in a progressive reduction in CIRCE-bound HrcA, with increasing amounts of NH-HagF producing a concentration-dependent decrease in DNA-bound HrcA (Fig 3G). Quantification of four independent experiments confirmed a statistically significant reduction in HrcA binding in the presence of HagF (Fig 3H). Together with the pulldown, structural modeling, and BLI data, these results demonstrate that HagF directly interacts with HrcA, weakens HrcA self-association, and reduces HrcA association with CIRCE DNA. The AlphaFold3 model and BLI data support a mechanism in which HagF attenuates HrcA repression by destabilizing HrcA-containing DNA-binding complexes, while the CIRCE-binding assay shows that HagF functionally interferes with HrcA association with its target DNA.

### HagF inhibits the transcriptional repression activity of HrcA in *Chlamydia*

To determine whether HagF modulates HrcA activity *in vivo*, we quantified the expression of HrcA target genes in *C. trachomatis* under conditions of *hagF* overexpression or silencing. ATC-induced HagF overexpression in L2/HagF cultured at 37 °C led to consistent increases in the expression of *hrcA*, *grpE*, *dnaK*, *groES*, and *groEL* (Fig 4A). In contrast, ATC-induced overexpression of a σ^66^-binding-deficient GrgA variant, did not increase expression of these HrcA-regulated heat shock genes, as demonstrated by RT-qPCR analysis (S9 Fig) and RNA-seq analysis [55]. These control experiments argue against a nonspecific protein-overexpression effect and support the conclusion that HagF specifically inhibits HrcA-mediated transcriptional repression. Conversely, ATC-induced dC12-mediated *hagF* silencing in both L2/dC12-hagF-g1 and L2/dC12-hagF-g2 had no detectable effect on the expression of these genes at 37 °C (Fig 4B) but significantly reduced expression of *hrcA*, *grpE*, and *dnaK*, though not *groES* or *groEL*, at 40.5 °C (Fig 4C). These findings indicate that HagF promotes derepression of the *hrcA-grpE-dnaK* operon during heat shock and suggest differential sensitivity of HrcA-regulated promoters to HagF-dependent antagonism.

**Fig 4:**
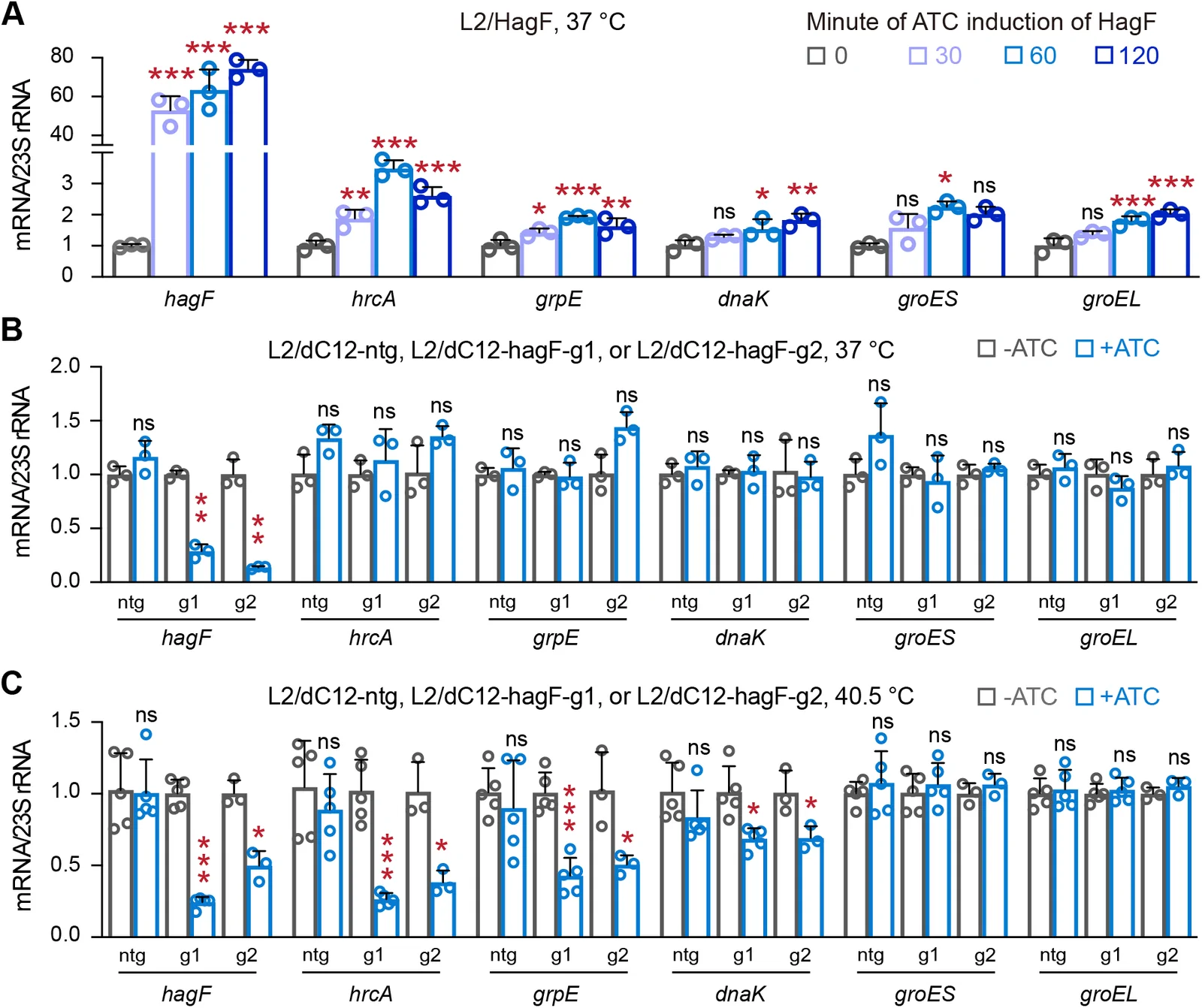
HagF inhibits HrcA-mediated transcriptional repression in *Chlamydia.* (A) HagF overexpression increases the expression of HrcA target genes. In L2/HagF, which expresses untagged HagF from a recombinant plasmid, ATC was added at 14, 15, or 15.5 hpi to induce HagF expression. RNA was extracted at 16 hpi, and transcript levels of *hrcA*, *grpE*, *dnaK*, *groES*, and *groEL* were quantified by RT-qPCR. (B, C) *hagF* silencing does not affect expression of HrcA target genes under non-stress conditions but reduces expression of the *hrcA–grpE–dnaK* operon during heat stress. L2/dC12-hagF-g1 and L2/dC12-hagF-g2 transformants expressing ATC-inducible dC12 were cultured at either 37 °C (B) or 40.5 °C (C). ATC was added at 0 hpi to induce *hagF* silencing, and RNA was extracted at 18 hpi for RT-qPCR analysis. Expression levels of *hrcA*, *grpE*, *dnaK*, *groES*, and *groEL* were measured relative to the 23S rRNA reference. Data represent the mean ± SD of biological replicates.

### Unregulated HagF expression inhibits chlamydial growth at 37 °C

To assess the physiological consequences of HagF-mediated antagonism of HrcA, we next examined the effects of HagF overexpression in *Chlamydia*. Because HagF overexpression increases the expression of HrcA-regulated chaperone genes, we reasoned that excessive derepression of these genes might impair chlamydial growth under non-stressed conditions. Consistent with this possibility, induction of HagF expression in L2/CH-HagF cultures resulted in smaller inclusions compared with uninduced cultures with reduced mKate fluorescence intensity compared with uninduced cultures (Fig 5A–C). In addition, L2/HagF exhibited significantly slower genome replication kinetics (Fig 5D) and yielded reduced infectious progeny (Fig 5E). These findings indicate that tight repression of HagF under non-stressed conditions is important for maintaining normal chlamydial growth and infectious progeny yield.

**Fig 5:**
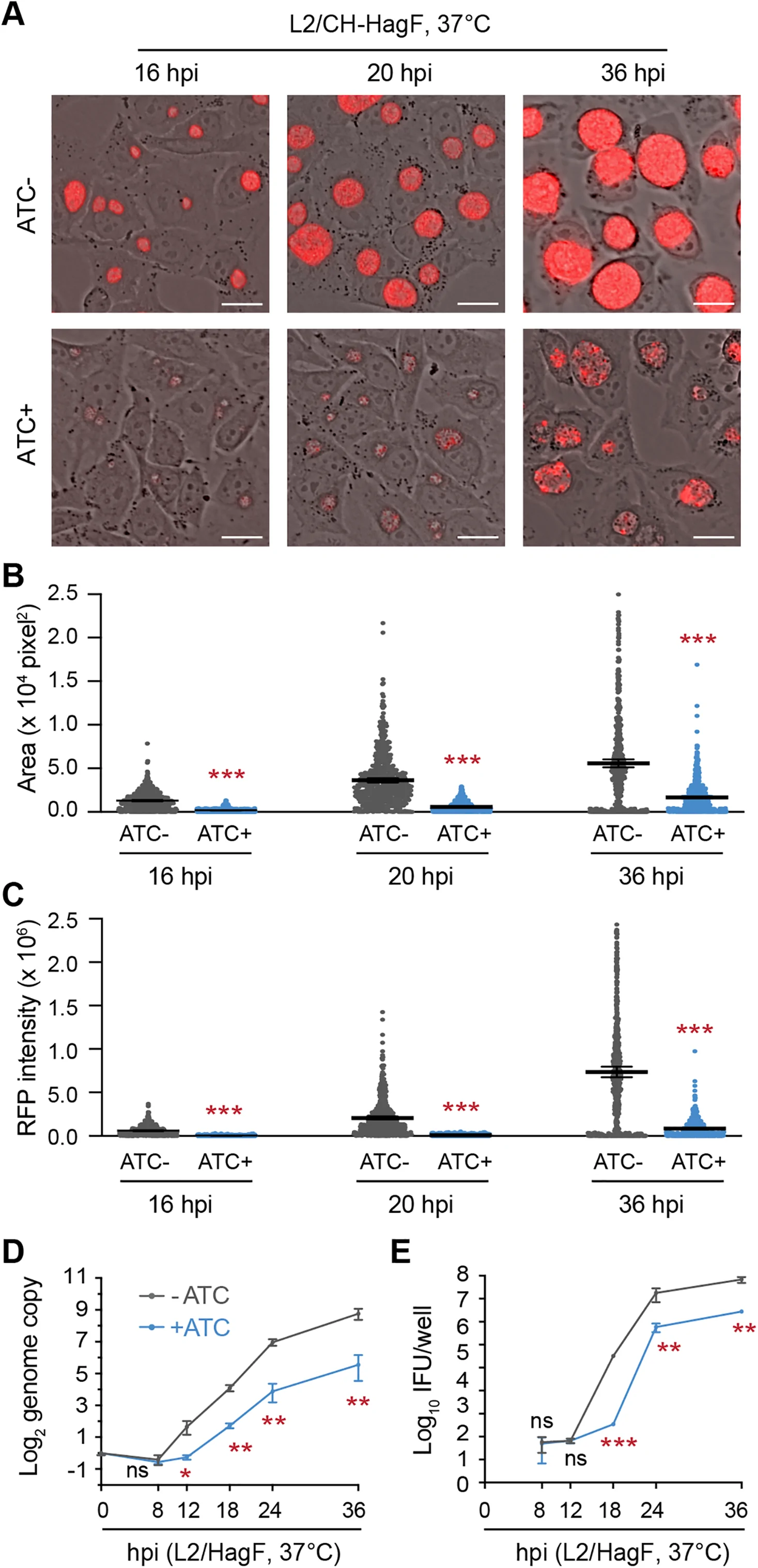
Forced HagF overexpression disrupts chlamydial growth and infectious progeny yield under non-stressed conditions. (A) ATC-induced HagF overexpression in L2/CH-HagF results in smaller inclusions with reduced red fluorescence intensity. Representative fluorescence microscopy images are shown at the indicated time points. Scale bars, 20 µm. (B) Quantification of inclusion size under ATC- and ATC+ conditions at the indicated time points. (C) Quantification of red fluorescent protein (RFP) intensity under ATC- and ATC+ conditions at the indicated time points. (B, C) Each point represents an individual inclusion. The long horizontal bar indicates the mean, and the shorter bars indicate the 95% confidence interval. (D) ATC-induced HagF overexpression in L2/HagF slows chlamydial genome replication. Genome copy number was quantified at the indicated time points. (E) ATC-induced HagF overexpression in L2/HagF reduces infectious progeny yield. Inclusion-forming units (IFUs) were measured at the indicated time points. (A-E) All cultures were maintained at 37 °C.

## Discussion

Bacterial survival under environmental stress requires precise regulation of transcriptional programs that maintain protein homeostasis [1,3–10]. One widely conserved regulator of this response is the transcriptional repressor HrcA, which suppresses expression of molecular chaperones under non-stressed conditions. In many bacteria, HrcA binds CIRCE elements upstream of the *hrcA-grpE-dnaK* and *groES-groEL* operons and represses transcription of these essential chaperone genes [23,27,34,36,56,57]. Heat stress disrupts HrcA activity, enabling rapid induction of chaperone expression and thereby restoring proteostasis. Although this regulatory system is broadly conserved, the composition and regulatory architecture of the HrcA regulon can vary among bacterial lineages [23,34,57].

In *Chlamydia*, the HrcA regulon has long been thought to consist only of the five classical chaperone genes [27,58]. Here, we identify *hagF* (*ctl0271*) as a sixth HrcA-regulated gene and reveal that the chlamydial heat shock response includes an additional regulatory component. Multiple lines of evidence support this conclusion, including the presence of a CIRCE-like element upstream of *hagF*, direct HrcA binding to its promoter, repression of *hagF* expression by HrcA overexpression, and strong induction of *hagF* following heat shock. These observations extend the known HrcA regulon and indicate that stress-responsive regulation in *Chlamydia* involves an additional layer of control beyond the canonical chaperone network.

While finalizing this study, Kozusnik et al. reported stress-induced expression of *ctl0271* (*hagF*) and its orthologs in *Chlamydia*-like organisms and proposed that these genes are HrcA targets [43]. In contrast to that proposed assignment, our study directly demonstrates HrcA binding to the ctl0271 promoter and HrcA-dependent repression in *C. trachomatis* and further shows that the encoded protein HagF is a direct antagonist of HrcA: HagF physically associates with HrcA, weakens HrcA self-association, and reduces HrcA binding to CIRCE DNA, thereby establishing a negative feedback loop that actively modulates chaperone gene expression and infectious progeny yield during heat stress.

The reduced expression of late, EB-enriched genes is consistent with impaired late developmental progression, which contributes to the decrease in infectious progeny yield. However, because late-stage IFU assay results reflect both the generation of infectious EBs and their ability to remain infectious, HagF may promote infectious progeny yield by supporting EB formation, preserving EB infectivity under heat stress, or both. This phenotype is notable given the reduced chlamydial genome and underscores the importance of adaptive regulatory mechanisms for maintaining developmental fitness under restrictive conditions. The conservation of HagF across sequenced members of the order Chlamydiales, including both *Chlamydia* and *Chlamydia*-like organisms, suggests a shared evolutionary requirement to couple stress responses with developmental progression. Although many chlamydial and host factors have been implicated in persistence under stress, relatively few bacterial proteins are known to support productive developmental output under restrictive conditions [7,8,15–18]. Thus, our work reveals a previously unrecognized Chlamydiales-specific mechanism that links stress-responsive transcriptional regulation to infectious progeny yield.

Mechanistically, HagF antagonizes HrcA by reducing its ability to associate with CIRCE DNA. Reciprocal pulldown assays support a direct association between HagF and HrcA. AlphaFold3 modeling further predicts that HagF and HrcA can form a 2HagF–2HrcA heterotetramer in which the HrcA–HrcA interface is reduced relative to the predicted HrcA homodimer. Consistent with this predicted structural model, BLI analysis showed that HrcA bound HagF with an apparent affinity comparable to HrcA self-association, whereas a preformed HrcA–HagF complex bound additional HrcA with reduced apparent affinity, reflected by decreased apparent *k*_on_ and increased apparent *k*_off_. These data indicate that HagF weakens, rather than abolishes, HrcA self-association. The predicted 2HagF–2HrcA architecture and specific interface residues remain to be experimentally established. Consistent with this mechanism, HagF reduced the ability of HrcA to associate with CIRCE DNA *in vitro*. Thus, HagF functions as a direct antagonist of HrcA. The precise mechanism by which HagF reduces HrcA-CIRCE association remains to be determined and may involve weakened HrcA self-association, allosteric or steric inhibition of DNA binding, or a combination of these mechanisms.

Our findings reveal a heat-responsive regulatory circuit in which HagF functions as a stress-induced antagonist of the HrcA repressor. Under non-stressed conditions, HrcA represses *hagF* along with the *hrcA-grpE-dnaK* and *groES-groEL* operons (Fig 6, left). GroEL or the GroESL chaperonin complex stabilizes HrcA and reinforces its repressive activity, forming a positive feedback loop that maintains repression of chaperone genes. Under heat stress, HrcA destabilization provides the initial relief of repression and permits induction of *hagF*. Accumulated HagF then binds residual HrcA and further attenuates its repressor activity, thereby amplifying chaperone gene expression (Fig 6, right). This model does not require preferential binding of HagF to a heat-destabilized HrcA species; rather, HagF provides an additional layer of derepression by antagonizing residual functional HrcA during heat stress. This HagF-mediated negative feedback loop complements the GroESL-mediated positive feedback and enables graded amplification of the heat shock response.

**Fig 6:**
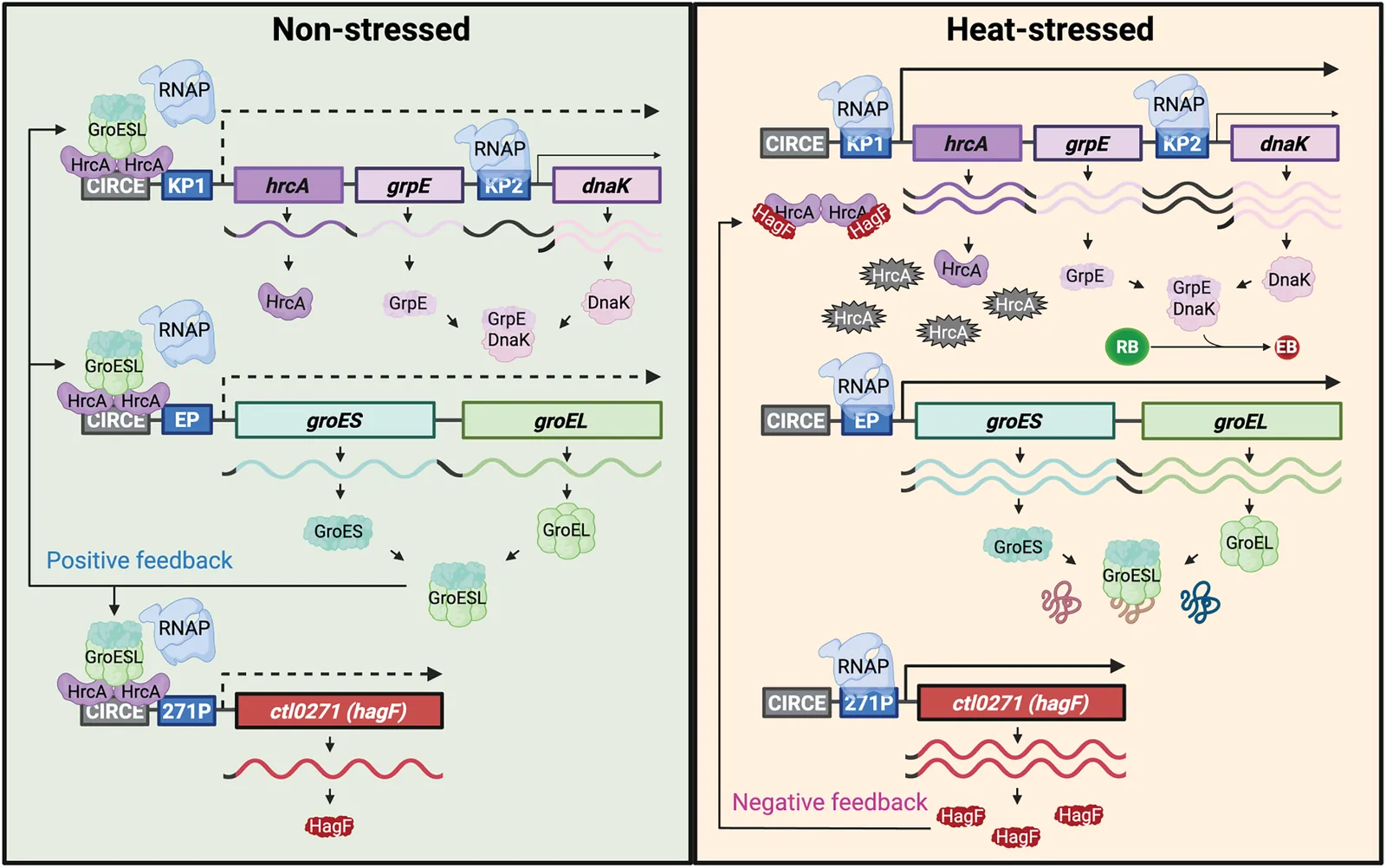
Model for dual feedback regulation of HrcA by GroESL and HagF in *Chlamydia.* (Left) Under non-stressed conditions, HrcA binds CIRCE elements upstream of the *hrcA-grpE-dnaK* and *groES-groEL* operons, as well as upstream of *ctl0271* (*hagF*), repressing transcription from the KP1, EP, and 271P promoters. The GroEL-GroES chaperonin complex, GroESL, enhances HrcA function, likely by stabilizing HrcA, forming a broadly conserved positive feedback loop that maintains repression of chaperone genes in the absence of stress. Although not depicted, GroEL alone has also been shown to enhance HrcA activity in *Chlamydia* [31]. (Right) Under heat-stressed conditions, HrcA becomes destabilized and its DNA-binding activity is reduced, resulting in increased transcription from the KP1, EP, and 271P promoters. Concurrently, heat-induced expression of *hagF* leads to HagF accumulation. HagF binds residual HrcA and weakens HrcA self-association, thereby further reducing HrcA–CIRCE association and attenuating HrcA-mediated repression. This HagF-mediated negative feedback loop amplifies the heat shock response, with a particularly important effect on induction of the *hrcA-grpE-dnaK* operon. By enhancing chaperone gene expression, HagF promotes the yield of infectious progeny under heat stress. Figure created in BioRender. Wang, Y. (2026) https://BioRender.com/jbrm4re.

Interestingly, *hagF* silencing during heat stress selectively reduced expression of the *hrcA–grpE–dnaK* operon but not the *groES–groEL* operon, suggesting differential promoter sensitivity to HagF-dependent HrcA antagonism. One possible explanation is that the KP1 CIRCE element, which is more highly conserved than the EP CIRCE element (Fig 1C), may bind residual functional HrcA more effectively during heat stress. In this scenario, KP1 would retain greater HrcA-mediated repression when HagF is depleted, making expression from the *hrcA–grpE–dnaK* operon more dependent on HagF-mediated relief of HrcA repression. By contrast, the *groES–groEL* promoter may be sufficiently derepressed by heat-induced HrcA destabilization alone. The strong reduction in infectious progeny yield under heat stress further indicates that, under restrictive conditions, enhanced expression of the DnaK–GrpE chaperone system may be particularly important for productive differentiation, maintenance of EB infectivity, or both. Notably, while GroEL-mediated modulation of HrcA is broadly conserved among bacteria [31–33], HagF-mediated HrcA antagonism appears restricted to members of the *Chlamydiales*. Thus, the chlamydial HrcA system combines a broadly conserved positive feedback mechanism with a lineage-specific negative feedback mechanism to fine-tune chaperone gene expression during heat stress.

In summary, our study identifies *hagF* as a heat-induced, HrcA-repressed gene that encodes a direct antagonist of HrcA in *Chlamydia*. This mutually inhibitory relationship integrates thermal stress signals with transcriptional and developmental control. By promoting the yield of infectious progeny under heat stress, HagF enhances chlamydial fitness under restrictive conditions. More broadly, these findings suggest that antagonist-based modulation may represent a flexible mechanism for tuning conserved stress responses in bacteria. Together with the recent observation that HagF orthologs are upregulated by heat shock and other stressors in environmental chlamydiae [42], these findings expand the HrcA regulatory paradigm and reveal a *Chlamydiales*-specific strategy for preserving developmental competence under adverse conditions.

## Materials and methods

### Plasmids

Plasmids pTRL2-HagF, pTRL2-NH-HagF, and pTRL2-CH-HagF were constructed for the expression of untagged HagF, N-terminally His-tagged HagF, and C-terminally His-tagged HagF, respectively, under the control of an ATC-inducible promoter in *C. trachomatis*. All three plasmids were generated using the NEBuilder HiFi DNA Assembly Kit (New England Biolabs) as described previously [52,55]. Plasmid backbone fragments were amplified from pASK-GFP-L2-mKate2 [59] or pTRL2-NHGrgA [55] as the template, while the HagF insert was amplified from the L2 genomic DNA.

Plasmids pTRL2-dC12-ntg, pTRL2-dC12-hagF-g1, and pTRL2-dC12-hagF-g2 were constructed for ATC-inducible dCas12 (dC12)-mediated gene silencing in *C. trachomatis*. pTRL2-dC12-ntg, which expresses a guide RNA (gRNA) with no predicted target site in *C. trachomatis*, was generated by assembling a plasmid backbone (PCR-amplified from pTRL2-His-GrgA-67m [52]) and a CRISPR/dC12 array fragment amplified from pBbS8c-ddcpf1-rfp [39], a gift from Nigel S. Scrutton. pTRL2-dC12-hagF-g1 and pTRL2-dC12-hagF-g2 were derived from pTRL2-dC12-ntg by replacing the ntg sequence with gRNAs targeting *hagF*. The sequences of the gRNAs were 5′-CTCGGCATTGTAGTTAAGGGGTA-3′ and 5′-TATTTCCCCATACATTTCTGCTC-3′ for g1 and g2, respectively.

For recombinant protein expression in *Escherichia* coli, plasmids pET28a-NH-HrcA, pET21a-NS-HrcA, pET28a-NH-HagF, and pET21a-NS-HagF were constructed to express N-terminally His-tagged or Strep II-tagged versions of HrcA and HagF. These plasmids were derived by replacing the *grgA* coding sequence in pET28a-NH-GrgA or pET21a-NS-GrgA as described previously [51].

The identities of all plasmids were confirmed by Sanger or Nanopore sequencing (Quintara Biosciences, Boston, MA).

### Host cells and culture conditions

Human cervical epithelial HeLa 229 cells were purchased from ATCC. Murine fibroblast L929 cells were a kind gift from Dr. Terence S. Dermody (Vanderbilt University). Both HeLa and L929 cells were maintained as monolayers at 37 °C in 5% CO_2_ using Dulbecco’s modified Eagle’s medium (DMEM) supplemented with 4.5 g/L glucose, 0.11 g/L sodium pyruvate, 20 µg/mL gentamicin, and either 10% (HeLa) or 5% (L929) fetal bovine serum (FBS) (Gibco, A52567-01).

### Chlamydiae and culture conditions

Wildtype *C. trachomatis* serovar L2 strain 434/BU (L2) was obtained from the American Type Culture Collection (ATCC) and expanded in our laboratory using L929 cells as host cells. The L2/NH-HrcA strain, which expresses N-terminally His-tagged HrcA, has been described previously [55]. The following L2 transformants were generated by transforming wildtype L2 with ATC-inducible shuttle vectors as indicated: L2/dC12-ntg (pTRL2-dC12-ntg), L2/dC12-hagF-g1 (pTRL2-dC12-hagF-g1), L2/dC12-hagF-g2 (pTRL2-dC12-hagF-g2), L2/HagF (pTRL2-HagF), L2/NH-HagF (pTRL2-NH-HagF), and L2/CH-HagF (pTRL2-CH-HagF). Transformations and selection were performed as previously described [52]. Clonal populations of the transformants were generated by limiting dilution [52]. EBs were centrifuged through a 35% MD-76R gradient to remove host cell debris, further separated from RBs by centrifugation through a 40%/44%/52% MD-76R discontinuous gradient as described previously [60]. Purified EBs were resuspended in the EB storage solution SPG (0.25 M sucrose, 10 mM sodium phosphate, 5 mM L-glutamate, pH 7.2), aliquoted, and stored at −80 °C.

### Synchronized infection for growth assays and gene expression analysis

Near-confluent HeLa monolayers cultured in 6-, 12-, or 24-well plates were infected with MD-76R gradient-purified EBs, typically at a multiplicity of infection of approximately one inclusion-forming unit (IFU) per host cell. This condition resulted in 80–90% infection efficiency, as assessed by fluorescence microscopy. To synchronize infection, inoculated monolayers were centrifuged at 900 × g and 25 °C for 20 min, then washed three times with Hank’s Balanced Salt Solution. The endpoint of the wash step was designated as 0 hour post-infection (hpi). Infected cells were then cultured in DMEM supplemented with 5% FBS and 20 µg/mL gentamicin in a humidified 37 °C incubator with 5% CO_2_.

### Quantification of chlamydial genome copy number

Chlamydial genome copy number was used as a quantitative measure of total bacterial load, including both reticulate bodies (RBs) and elementary bodies (EBs). This method is particularly useful for assessing RB replication, since RBs are non-infectious and cannot be quantified by inclusion-forming unit (IFU) assays (see below). Genome copy number thus serves as a complementary metric to IFU-based progeny analysis.

At the indicated time points, culture medium was replaced with 0.5 mL SPG per well (12- or 24-well plates) or 1.0 mL per well (6-well plates). Infected cells were detached using Cell Lifters (Corning) and collected into 4.5 mL culture tubes. The cell suspensions were sonicated using a 130-watt ultrasonic processor equipped with a 3-mm tip, set to 35% amplitude. Sonication was applied for a total of 12 seconds in alternating 2-second on/off pulses to lyse host cells and release chlamydiae. Chlamydiae were pelleted by 10-min centrifugation at 20,000 x g and 4 °C. After carefully removing the supernatant, the pellet was resuspended in 100 µL of alkaline lysis buffer (0.1 M NaOH, 0.2 mM EDTA). Lysates were heated at 95 °C for 15 min and then neutralized by adding 400 µL of neutralization buffer (25 mM Tris-HCl, pH 7.2). The neutralized DNA samples were stored at –20 °C for subsequent analysis.

Relative chlamydial chromosome abundance was quantified by qPCR using primers targeting *grgA*. Each reaction was performed in technical duplicates using DNA samples from three biological replicates. Reactions were carried out on a QuantStudio 5 Real-Time PCR System using Power SYBR Green PCR Master Mix (Thermo Fisher Scientific, catalog #A25778).

### Inclusion-forming unit (IFU) assay

The IFU assay is functionally equivalent to a colony-forming unit assay and was used to quantify infectious EB particles in either purified EB stocks or crude cell lysates based on their ability to form inclusions in host cells. To prepare cell lysates, infected cells on multi-well plates were collected and lysed, similarly to genome copy quantification assays. Lysates were centrifuged at 500 x g for 10 min at 4 °C to remove cell debris and stored at –80 °C until further use.

EB stocks or clarified lysates from infected cultures were thawed, serially diluted 1:10 with culture medium (DMEM containing 5% fetal bovine serum, 20 µg/mL gentamicin, and 1 µg/mL cycloheximide) on 96-well plates with 2- to 3-hour-old confluent L929 cell monolayers. Plates were centrifuged at 900 x g and 25 °C for 20 min and then incubated at 37 °C for 30 hours. Since all chlamydial transformants used in this study express the far-red fluorescent protein mKate, red fluorescent inclusions were counted in live cultures using an Olympus IX-50 fluorescence microscope [52].

### Inclusion size and red fluorescence intensity quantification

Inclusion size and red fluorescence protein (RFP) intensity, both correlating with chlamydial growth, were quantified as previously described [61] with modifications. Before imaging, the culture medium was replaced with phosphate-buffered saline (PBS) containing calcium and magnesium to minimize background fluorescence. Brightfield and red fluorescence images were acquired using an Olympus IX51 fluorescence microscope with an Infinity i8-3 CMOS camera, maintaining constant exposure settings across all samples. Image overlay was performed using ACINST03 software. Inclusion size and RFP intensity were quantified using the Java-based ImageJ software.

### Chromatin immunoprecipitation (ChIP)

To prepare *C. trachomatis* cultures for ChIP, two 150-mm dishes of L929 cells were infected with L2/NH-HrcA and cultured in medium supplemented with cycloheximide (1 µg/mL final concentration). At 14 hpi, ATC was added to a final concentration of 10 nM to induce HrcA expression. At 16 hpi, crosslinking and DNA fragmentation were performed as previously described [62] with modifications. The medium was replaced with 15 mL of 1% formaldehyde in PBS, and crosslinking was performed at room temperature for 20 min. Formaldehyde was then quenched by replacing it with 20 mL of quench solution (PBS containing 0.25 M glycine, pH 7.5). After incubation at room temperature for 15 min, the quench solution was aspirated, and cells were scraped into 15 mL of quench solution containing 0.2% bovine serum albumin (BSA), which were subject to centrifugation at 500 × g for 10 min at 4 °C.

The resulting pellet was resuspended in 1 mL of lysis buffer, made of 25 mM Tris-HCl (pH 7.5), 150 mM NaCl, 0.5% sodium deoxycholate, 1% NP-40, 0.1% SDS, and protease inhibitor cocktail cOmplete (Roche). Cells were lysed by sonication using a 130-watt Ultrasonic Processor (3-mm tip, 35% amplitude) in 5-second on/off cycles for 9 min, yielding DNA fragments between 300 and 1200 bp. The sonicated lysate was clarified by centrifugation at 6,000 × g for 10 min at 4 °C and pre-cleared by incubation with 50 µL (bed volume) of Protein A/G Sepharose beads (Millipore Sigma, catalog # IP-10) for 2 h at 4 °C on a Nutator.

While pre-clearing was in progress, 5 µg of either monoclonal anti-His antibody (GenScript, A00186) or control mouse IgG (Millipore Sigma, M9269) was mixed with 80 µL of washed Protein A/G Sepharose in a 1.5-mL tube. After 1 h of rotation at 4 °C, 1 mL of 5% BSA was added, and the tubes were incubated for 2 h at 4 °C to block nonspecific binding. BSA and free antibodies were removed, and 250 µL of pre-cleared lysate was added to the antibody-coated beads, followed by overnight incubation on a Nutator at 4 °C.

Beads were washed sequentially with the following buffers: low-salt wash buffer (20 mM Tris-HCl, pH 8.1, 150 mM NaCl, 2 mM EDTA, 1% Triton X-100, 0.1% SDS), high-salt wash buffer (same as above, but with 500 mM NaCl), LiCl wash buffer (10 mM Tris-HCl, pH 8.1, 0.25 M LiCl, 1 mM EDTA, 1% NP-40, 1% sodium deoxycholate), and twice with TE buffer (10 mM Tris-HCl pH 8.1, 1 mM EDTA).

Protein-DNA complexes were eluted using 200 µL of freshly prepared elution buffer (1% SDS, 0.1 M NaHCO_3_). After brief centrifugation, supernatants were transferred to new tubes. To reverse crosslinking, 8 µL of 5 M NaCl and 4 µL of RNase A (10 mg/mL) were added, and tubes were incubated overnight at 65 °C with shaking. Protein digestion was performed by adding 2 µL of proteinase K (20 mg/mL), 16 µL of 0.5 M EDTA (pH 8.0), and 8 µL of 1 M Tris (pH 6.5), followed by incubation at 45 °C for 2 h with shaking. ChIP DNA was purified using the QIAquick PCR Purification Kit (Qiagen) and stored at –20 °C until further use.

### Quantification of gene promoters in ChIP DNA

Enrichment of target gene promoters in ChIP DNA was assessed by qPCR using DNA precipitated with anti-His antibody or control IgG and a non-precipitated input DNA control. PCR reactions were performed using promoter-specific primers (S3 Table) as described for genome copy number qualification. Promoter enrichment was calculated relative to input DNA and compared between anti-His and IgG ChIP to assess HrcA binding specificity.

### RNA extraction and purification

*C. trachomatis*-infected cells cultured in 6- or 24-well plates were lysed directly in TRI reagent (Millipore Sigma, catalog no. 93289). Total RNA was extracted from the aqueous phase according to the manufacturer’s instructions and treated with two successive rounds of DNase I-XT digestion (New England Biolabs) to remove residual genomic DNA. Complete DNA removal was verified by PCR analysis [52]. RNA concentration and purity were determined using Qubit RNA assay kits (Thermo Fisher Scientific). DNA-free RNA was aliquoted and stored at –80 °C until use.

### Quantification of gene expression

We performed reverse transcription and quantitative PCR (RT-qPCR) using a QuantStudio 5 Real-Time PCR System (Thermo Fisher Scientific) and the Luna Universal One-Step RT-qPCR Kit (New England BioLabs, catalog #E3005E) to quantify gene expression in *C. trachomatis* as described previously [52,55]. RNA samples stored at –80 °C were thawed on ice and diluted 1:200 in nuclease-free water. Each 10 µL reaction contained 5 µL of Luna Universal One-Step Reaction Mix, 0.5 µL of 20X Luna WarmStart Reaction Enzyme Mix, 0.4 µL each of 10 µM forward and reverse primers, and 3.7 µL of diluted RNA. In addition to assays for target genes (*hagF*, *hrcA*, *grpE*, *dnaK*, *groES*, *groEL*, *dC12, omcA,* and *omcB*), RT-qPCR was also performed for 23S rRNA, which served as an input reference. We selected 23S rRNA as the input reference because its expression is unaffected by short-term (≤30 min) heat shock or short-term (≤ 2 h) gene overexpression [5,55], and *hagF* knockdown did not affect genome replication under the conditions used in this study. Relative gene expression levels were calculated using the 2^–ΔΔCT method [63]. All experiments were conducted using three or five biologically independent RNA samples, each measured in technical duplicate. Information for RT-qPCR primers is provided in S3 Table.

### Expression of His- and Strep II-tagged proteins in and their purification from *E. coli*

*E. coli* BL21 (DE3) pLysS cells were transformed with plasmids pET28a-NH-HrcA, pET21a-NS-HrcA, pET28a-NH-HagF, or pET21a-NS-HagF. Protein expression was induced as previously described [51]. His-tagged proteins (NH-HrcA and NH-HagF) were purified using HisPur cobalt Sepharose (Thermo Fisher Scientific) following established protocols [51]. Strep II-tagged proteins (NS-HrcA and NS-HagF) were purified using Strep-Tactin XT Sepharose according to the manufacturer’s instructions.

### Protein pulldown assays

Pulldown assays were performed as previously described with modifications [64]. For each reaction, 20 µg of purified NH-HagF or NH-HrcA was incubated with 20 µg of recombinant NS-HrcA or NS-HagF, respectively, in a total volume of 40 µL. In negative control reactions, NH-HagF or NH-HrcA was replaced with protein storage buffer containing 50 mM Tris-HCl (pH 7.5), 150 mM NaCl, and 10% glycerol. Protein mixtures were incubated at 37 °C for 30 min. During the incubation, 50 µL of HisPur cobalt Sepharose suspension (Thermo Fisher Scientific) was washed twice with 1 mL of HisPur purification wash buffer containing 50 mM imidazole, followed by two washes with 200 µL of wash buffer containing 10 mM imidazole. Protein mixtures were then added to the imidazole-prewashed beads and incubated on a Nutator at 4 °C for 1 h. After binding, beads were washed five times with 1 mL of wash buffer containing 10 mM imidazole and 1% NP-40, followed by two washes with 1 mL of wash buffer containing 50 mM imidazole. Bound proteins were eluted in 30 µL of elution buffer (50 mM sodium phosphate, pH 7.4, 300 mM NaCl, 150 mM imidazole). Eluted proteins were resolved by 12% SDS-PAGE and transferred to a PVDF membrane. Membranes were blocked with 3% BSA and probed with Strep-Tactin HRP conjugate (IBA Life Sciences, catalog # 2-1502-001). Signal was detected by chemiluminescence (Millipore Sigma, catalog # OR03L).

### Biolayer interferometry (BLI) assays

Biolayer interferometry (BLI) assays were performed using an Octet N1 instrument. NH-HrcA, NS-HrcA, and NS-HagF were dialyzed overnight at 4°C against 1,000 volumes of pre-chilled BLI buffer containing 25 mM Tris-HCl, 150 mM NaCl, 0.1 mM EDTA, 10 mM MgCl_2_, and 0.1 mM DTT, pH 8.0. Ni-NTA biosensors were hydrated in 200 μL of BLI buffer for at least 10 min before use. Hydrated biosensors were mounted onto the instrument and equilibrated in BLI buffer for 30 s to establish a baseline. NH-HrcA, either alone or following preincubation with a 10-fold molar excess of NS-HagF, was loaded onto Ni-NTA biosensors by incubating the sensor tips with 4 μL of protein solution for 240 s. Biosensors were then washed in BLI buffer for 30 s to remove unbound protein. Association was measured by incubating ligand-loaded biosensors with 4 μL of NS-HrcA or NS-HagF for 180 s, and dissociation was measured by transferring the biosensors into BLI buffer for 180 s. For each ligand–analyte pair, three independent BLI experiments were performed. In each experiment, binding was measured using three analyte concentrations, and the association and dissociation data from these concentrations were globally fitted using Octet N1 software version 1.4 to generate a set of apparent kinetic parameters. The *k*_on_, *k*_off_, and *K*_D_ values reported in S2 Table represent the mean ± SD of the three independently derived parameter sets.

### *In vitro* HrcA-CIRCE DNA binding assay

A biotinylated 237-bp fragment containing the CIRCE element of the KP1 promoter was generated by PCR using *C. trachomatis* L2 genomic DNA as template and primers [BtnTg]-5′-ATACAAGAAAGATCCCTCCA-3′ and 5′-CGTATCGTTGCCGAACT-3′. The PCR product was purified using the QIAquick PCR Purification Kit (Qiagen, catalog #28104) and quantified using a NanoDrop spectrophotometer.

For each DNA-binding assay, 30 μL Streptavidin MagBeads (GenScript, catalog #L00936) was washed three times with 1 mL CIRCE-binding assay (CBA) buffer consisting of 40 mM Tris-HCl (pH 8.0), 135 μM EDTA, 100 μM DTT, and 7.5% glycerol (vol/vol). Beads were then incubated with 1 μg biotinylated DNA in a total volume of 50 μL CBA buffer on a Nutator at room temperature for 30 min. DNA-bound beads were washed three times with CBA buffer to remove unbound DNA.

3 μg NH-HrcA was incubated alone or together with 3, 6, or 12 μg NH-HagF in a total volume of 40 μL protein storage buffer. After incubation at room temperature for 10 min, NH-HrcA or NH-HrcA-NH-HagF mixtures were added to the CIRCE-bound MagBeads. Each reaction was adjusted to a final volume of 300 μL with CBA buffer. Following incubation on a Nutator at 4 °C for 1 h, beads were washed four times with CBA buffer. Bound proteins were eluted by adding 40 μL SDS-PAGE sample buffer and heating at 95 °C for 5 min. Eluted proteins were resolved by SDS-PAGE and analyzed by western blot using anti-His antibody to detect NH-HrcA. Band intensities were quantified by densitometry using ImageJ software, and CIRCE-bound HrcA levels were normalized to the HrcA-only condition.

### Immunofluorescence assays

Cells cultured in 6-well plates were infected with L2/NH-HagF or L2/CH-HagF and incubated at 37 °C. At 16 hpi, anhydrotetracycline (ATC) was added to a final concentration of 10 nM to induce NH-HagF or CH-HagF expression. At 22 hpi, cells were fixed sequentially with 4% paraformaldehyde, then with cold methanol. NH-HagF and CH-HagF were detected using a mouse anti-His antibody (Abcam, ab9108) and a TRITC-conjugated goat anti-mouse IgG secondary antibody (Millipore Sigma, catalog #T5393). GrgA was visualized using a rabbit anti-GrgA antibody [55] and a FITC-conjugated goat anti-rabbit IgG (Immunotech, catalog # IM0833). Fluorescence images were acquired using an Infinity i8-3 CMOS monochrome camera. Image overlay and color merging were performed using ACINST03 software [52].

### Protein three-dimensional structure prediction

The three-dimensional structures of HagF, HrcA, the HrcA homodimer, and the 2HagF–2HrcA heterotetramer were predicted using the AlphaFold3 server [53]. Structure predictions were based on full-length amino acid sequences, and multimeric models were generated using AlphaFold’s multimer mode where applicable.

### Statistical analysis

Statistical comparisons of EB titer, genome copy number, and RNA expression between two groups were performed using two-tailed *t*-tests. Comparisons of inclusion size and RFP intensity between two groups were performed using the Mann-Whitney test. Where applicable, *P* values were adjusted for multiple comparisons using the Benjamini-Hochberg procedure to control the false discovery rate. Comparisons involving three or more groups were conducted using one-way ANOVA followed by Dunnett’s post hoc test. In figures, one, two, and three asterisks indicate *P* < 0.05, 0.01, and 0.001, respectively.

## Supporting information

S1 Fig Predicted σ^66^ promoter and CIRCE element upstream of hagF (ctl0271). (A) Consensus sequence of the chlamydial σ^66^ core promoter (1). (B) Sequence analysis of the intergenic region between *ctl0270* and *hagF* identified a putative CIRCE element (Fig 1) and a predicted σ^66^-dependent promoter. The transcriptional start site of *hagF* was experimentally mapped by deep-sequencing-based transcriptome analysis and is indicated in green (2). The predicted -35 (TGTACA) and -10 (TACAAT) elements, shown in red, each differ from the corresponding consensus elements of σ^66^ by a single nucleotide, with the unmatched nucleotide underlined.
(ZIP)

S2 Fig Sequence alignment of *hagF* orthologs from *Chlamydia* species and selected *Chlamydia-*like organisms. Amino acid sequence alignment of HagF orthologs from the indicated organisms. C. trac, *Chlamydia trachomatis*; C. suis, *Chlamydia suis*; C. muri, *Chlamydia muridarum*; C. sp., *Chlamydia* sp.; C. pneu, *Chlamydia pneumoniae*; C. cora, *Chlamydia corallus*; C. serp, *Chlamydia serpentis*; C. ibid, *Chlamydia ibidis*; C. peco, *Chlamydia pecorum*; C. croc, *Chlamydia crocodili*; C. poik, *Chlamydia poikilothermis*; C. cavi, *Chlamydia caviae*; C. feli, *Chlamydia felis*; C. abor, *Chlamydia abortus*; C. psit, *Chlamydia psittaci*; C. bute, *Chlamydia buteonis*; C. gall, *Chlamydia gallinacea*; C. aviu, *Chlamydia avium*; Parach, *Parachlamydia acanthamoebae*; and W. chon, *Waddlia chondrophila*. Conserved residues are highlighted.
(PDF)

S3 Fig *Bacteroides fragilis* S6L5 does not encode HagF. (A) Pairwise sequence alignment shows a high degree of identity between CTL0271 (HagF) and a hypothetical protein from *B. fragilis* S6L5 retrieved from NCBI. (B) Pairwise alignment reveals that the *B. fragilis* S6L5 protein is identical to the HagF ortholog from *Chlamydia gallinacea.*
(PDF)

S4 Fig *hagF* knockdown impairs infectious progeny formation over a physiologically relevant heat-stress range. L2/dC12-ntg (ntg), L2/dC12-hagF-g1 (g1), and L2/dC12-hagF-g2 (g2) transformants were cultured at the indicated temperatures in the absence or presence of ATC to induce dC12-mediated *hagF* knockdown. Inclusion-forming units (IFUs) in the culture at 36 h postinoculation were quantified and normalized to the corresponding non-ATC-treated controls. *hagF* silencing caused progressively greater reductions in infectious progeny formation as the temperature increased from 39 °C to 40.5 °C, with the strongest effect observed at 40.5 °C. No significant effect of ATC treatment was observed in the ntg strain. Cultures were also tested at 41 °C; however, no detectable IFUs were recovered from any strains under either ATC− or ATC+ conditions. Data represent the mean ± SD of biological duplicates (39 °C) or triplicates (40, 40.5, and 41 °C).
(PDF)

S5 Fig HagF localizes inside chlamydial cells HeLa 229 cells were infected with L2/NH-HagF (A) or L2/CH-HagF (B), fixed at 20 hpi, and immunostained. NH-HagF and CH-HagF were detected using a mouse anti-His antibody and TRITC-conjugated goat anti-mouse IgG. GrgA, a cytoplasmic chlamydial transcription factor, was detected using a rabbit anti-GrgA antibody and FITC-conjugated goat anti-rabbit IgG.
(PDF)

S6 Fig Structural alignment of the AlphaFold-predicted model of *C. trachomatis* HrcA (cyan) with the experimentally determined *Thermotoga maritima* HrcA structure (1STZ, grey). The root mean square deviation (RMSD) of the alignment is 2.6 Å.
(PDF)

S7 Fig AlphaFold3 models of HagF alone and in the HrcA-containing complex. (A) AlphaFold3-predicted structure of HagF modeled as a monomer. (B) HagF chain extracted from the AlphaFold3-predicted HagF–HrcA complex model. HagF appears more structurally ordered in the HrcA-containing model than when modeled alone.
(PDF)

S8 Fig Representative biolayer interferometry sensorgrams. Ni-NTA biosensors were loaded with NH-HrcA alone or NH-HrcA preincubated with NS-HagF as the immobilized ligand. Association was initiated by adding the indicated analyte, and dissociation was initiated by transferring the biosensor to BLI buffer at the wash step. (A) NS-HrcA binding to immobilized NH-HrcA. (B) NS-HagF binding to immobilized NH-HrcA. (C) NS-HrcA binding to immobilized NH-HrcA–NS-HagF complex. Analyte concentrations are indicated in each panel.
(PDF)

S9 Fig Overexpression of sigma-binding-deficient GrgA does not alter expression of HrcA-regulated heat shock genes. *C. trachomatis* L2/NH-GrgA Δ1–64 [1,2] were cultured in the absence or presence of ATC to induce expression of the sigma-binding-deficient GrgA variant. Transcript levels of *grgA, hrcA, grpE, dnaK, groES,* and *groEL* were quantified by RT-qPCR and normalized to 23S rRNA. ***, *P* < 0.001.
(ZIP)

S1 Table HrcA residues involved in the predicted HrcA–HrcA interface in HrcA homodimer and 2HagF–2HrcA heterotetramer models. Interface residues were identified from AlphaFold3-predicted HrcA–HrcA homodimer and 2HagF–2HrcA heterotetramer models. Residue counts refer to HrcA residues participating in the predicted HrcA–HrcA interface. In the 2HagF–2HrcA model, 42 of 43 HrcA interface residues overlap with the predicted HrcA homodimer interface.
(PDF)

S2 Table Biolayer interferometry assays were performed using His biosensors loaded with the NH-HrcA or NH-HrcA preincubated with a 10-fold molar excess of NS-HagF as the immobilized ligand. NS-HrcA or NS-HagF was used as the analyte. Representative graphs of recordings are shown in S8 Fig. Values of kinetic parameters (averages ± SDs) were generated by the Octet N1 1.4 software. *k*_on_, association rate constant; *k*_d_, dissociation rate constant; *K*_D_, dissociation equilibrium constant, calculated as *k*_off_/*k*_on_. P values were calculated using 2-tailed Student’s *t* tests, subjected to adjustment for multiple comparisons by Benjamini-Hochberg procedure.
(XLSX)

S3 Table qPCR and RT-qPCR primers. (PDF)

S1 Dataset Numerical data underlying the graphs, quantitative analyses, and tables. (XLSX)

S1 Rawgel Uncropped and Unadjusted Blot Images. (PDF)
