# Supplementary material for: A heat-induced HagF-HrcA regulatory loop promotes chlamydial fitness and infectious progeny yield under stress
Source: PLoS Pathog. 2026 Nov 4;22(11):e1014749. doi: 10.1371/journal.ppat.1014749 (PMC13750654; doi:10.1371/journal.ppat.1014749)
Supplement: Supplementary file 1 [file ppat.1014749.s002.pdf]

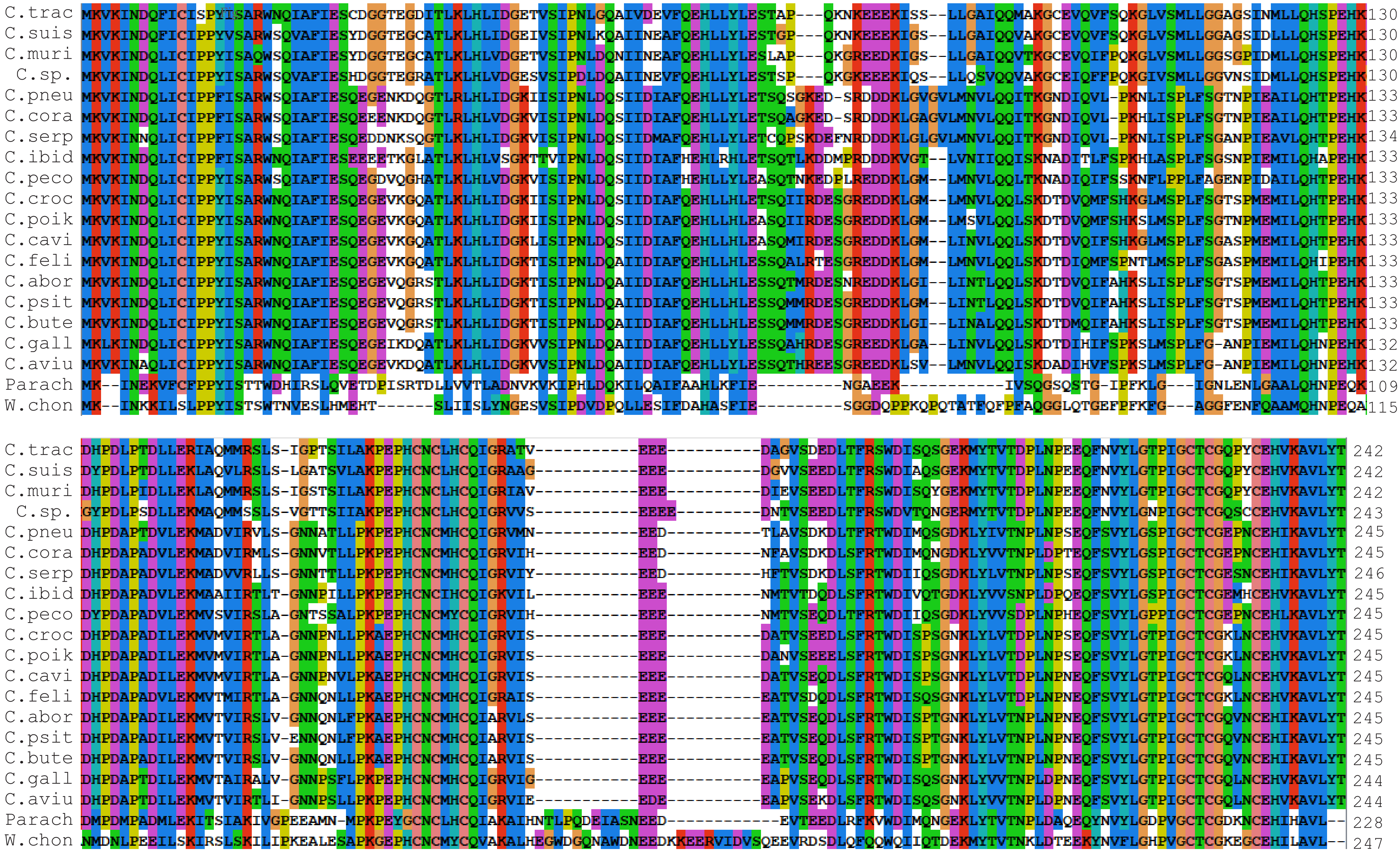

Fig. S2. Sequence alignment of *hagF* orthologs from *Chlamydia* species and selected *Chlamydia*-like organisms.
