# Supplementary material for: A heat-induced HagF-HrcA regulatory loop promotes chlamydial fitness and infectious progeny yield under stress
Source: PLoS Pathog. 2026 Nov 4;22(11):e1014749. doi: 10.1371/journal.ppat.1014749 (PMC13750654; doi:10.1371/journal.ppat.1014749)
Supplement: Supplementary file 2 [file ppat.1014749.s003.pdf]

**A**

```
C.trachomatis HagF           1   MKVKINDQFICISPYISARWNQIAFIESCDGGTEGDITLKLHLIDGETVSIPNLGQAIVD   60
                                 MK+KINDQ ICI PYISARWNQIAFIES +G   +   TLKLHLIDG+ VSIPNL QAI+D
B.fragilis S6L5 EYE60928.1   1   MKLKINDQLICIPPYISARWNQIAFIESQEGEIKDQATLKLHLIDGKVVSIPNLDQAIID   60

C.trachomatis HagF           61  EVFQEHLLYLESTAPQKN---KEEEKISSLLGAIQQMAKGCEVQVFSQKGLVSMLLGGAG   117
                                   FQEHLLYLES+   ++   +EE+K+ +L+  +QQ++K  ++ +FS K L+S L G A
B.fragilis S6L5 EYE60928.1   61  IAFQEHLLYLESSQAHRDESGREEDKLGALINVLQQLSKDTDIHIFSPKSLMSPLFG-AN   119

C.trachomatis HagF           118 SINMLLQHSPEHKDHPDLPTDLLERIAQMMRSLSIGPTSILAKPEPHCNCLHCQIGRATV   177
                                  I M+LQH+PEHKDHPD PTD+LE++   +R+L     S L KPEPHCNC+HCQIGR
B.fragilis S6L5 EYE60928.1   120 PIEMILQHNPEHKDHPDAPTDILEKMVTAIRALVGNNPSFLPKPEPHCNCMHCQIGRVIG   179

C.trachomatis HagF           178 EEEDAGVSDEDLTFRSWDISQSGEKMYTVTDPLNPEEQFNVVYLGTPIGCTCGQPYCEHVK   237
                                 EEE+A VS++DL+FR+WDISQSG K+Y VT+PL+P EQF+VYLGTPIGCTCGQ  CEHVK
B.fragilis S6L5 EYE60928.1   180 EEEEAPVSEQDLSFRTWDISQSGNKLYVVTNPLDPNEQFSVYLGTPIGCTCGQLNCEHVK   239

C.trachomatis HagF           238 AVLYT 242
                                 AVLYT
B.fragilis S6L5 EYE60928.1   240 AVLYT 244
```

**B**

```
B.fragilis S6L5 EYE60928.1      1   IAFQEHLLYLESSQAHRDESGREEDKLGALINVLQQLSKDTDIHIFSPKSLMSPLFGANP   60
                                    IAFQEHLLYLESSQAHRDESGREEDKLGALINVLQQLSKDTDIHIFSPKSLMSPLFGANP
C.gallinacea WP_021828457.1     1   IAFQEHLLYLESSQAHRDESGREEDKLGALINVLQQLSKDTDIHIFSPKSLMSPLFGANP   60

B.fragilis S6L5 EYE60928.1      61  IAFQEHLLYLESSQAHRDESGREEDKLGALINVLQQLSKDTDIHIFSPKSLMSPLFGANP   120
                                    IAFQEHLLYLESSQAHRDESGREEDKLGALINVLQQLSKDTDIHIFSPKSLMSPLFGANP
C.gallinacea WP_021828457.1     61  IAFQEHLLYLESSQAHRDESGREEDKLGALINVLQQLSKDTDIHIFSPKSLMSPLFGANP   120

B.fragilis S6L5 EYE60928.1      121 IEMILQHNPEHKDHPDAPTDILEKMVTAIRALVGNNPSFLPKPEPHCNCMHCQIGRVIGE   180
                                    IEMILQHNPEHKDHPDAPTDILEKMVTAIRALVGNNPSFLPKPEPHCNCMHCQIGRVIGE
C.gallinacea WP_021828457.1     121 IEMILQHNPEHKDHPDAPTDILEKMVTAIRALVGNNPSFLPKPEPHCNCMHCQIGRVIGE   180

B.fragilis S6L5 EYE60928.1      181 EEEAPVSEQDLSFRTWDISQSGNKLYVVTNPLDPNEQFSVYLGTPIGCTCGQLNCEHVKA   240
                                    EEEAPVSEQDLSFRTWDISQSGNKLYVVTNPLDPNEQFSVYLGTPIGCTCGQLNCEHVKA
C.gallinacea WP_021828457.1     181 EEEAPVSEQDLSFRTWDISQSGNKLYVVTNPLDPNEQFSVYLGTPIGCTCGQLNCEHVKA   240

B.fragilis S6L5 EYE60928.1      241 VLYT 244
                                    VLYT
C.gallinacea WP_021828457.1     241 VLYT 244
```

**Fig. S3. *Bacteroides fragilis* S6L5 does not encode HagF.** (A) Pairwise sequence alignment shows a high degree of identity between CTL0271 (HagF) and a hypothetical protein from *B. fragilis* S6L5 retrieved from NCBI. (B) Pairwise alignment reveals that the *B. fragilis* S6L5 protein is identical to the HagF ortholog from *Chlamydia gallinacea.*
