# Supplementary material for: A heat-induced HagF-HrcA regulatory loop promotes chlamydial fitness and infectious progeny yield under stress
Source: PLoS Pathog. 2026 Nov 4;22(11):e1014749. doi: 10.1371/journal.ppat.1014749 (PMC13750654; doi:10.1371/journal.ppat.1014749)
Supplement: Supplementary file 3 [file ppat.1014749.s004.pdf]

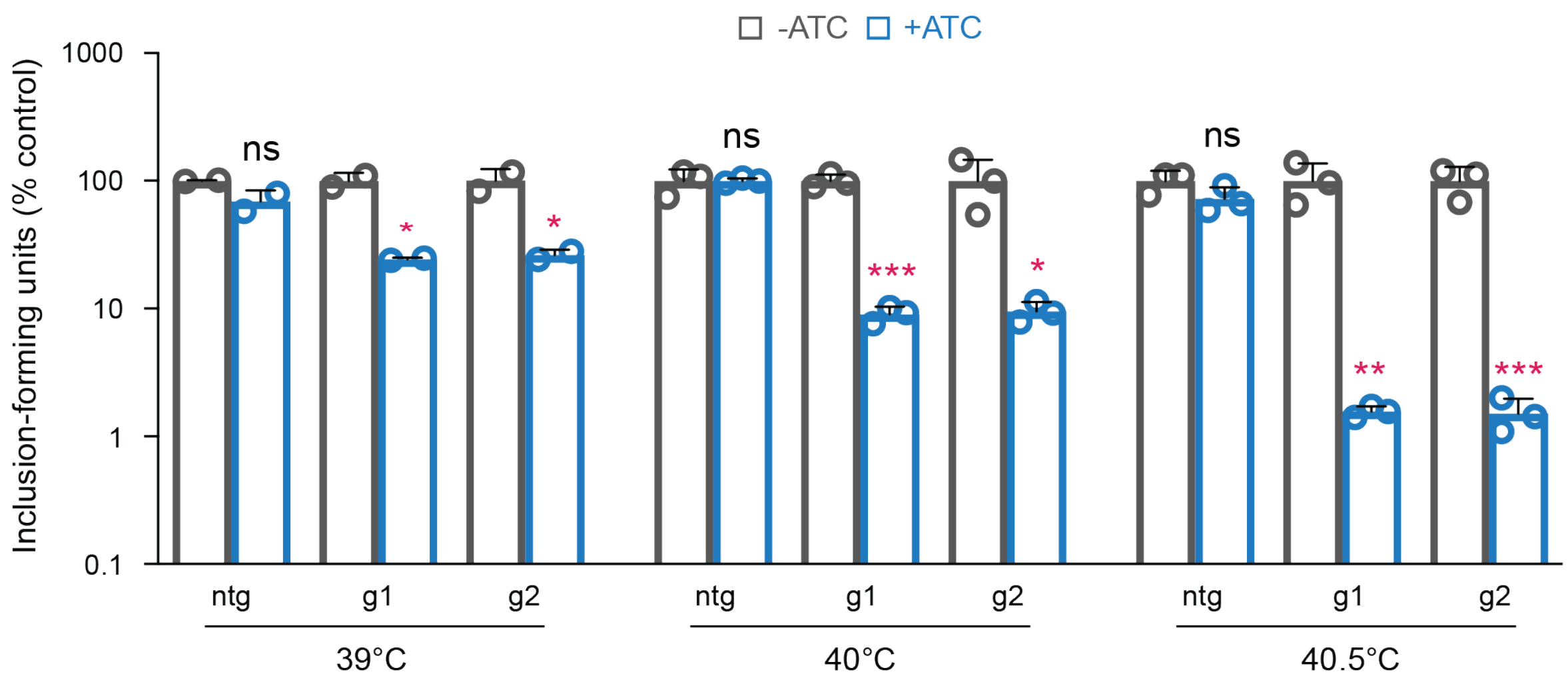

Fig. S4. *hagF* knockdown impairs infectious progeny formation over a physiologically relevant heat-stress range. L2/dC12-ntg (ntg), L2/dC12-hagF-g1 (g1), and L2/dC12-hagF-g2 (g2) transformants were cultured at the indicated temperatures in the absence or presence of ATC to induce dC12-mediated *hagF* knockdown. Inclusion-forming units (IFUs) in the culture at 36 h postinoculation were quantified and normalized to the corresponding non-ATC-treated controls. *hagF* silencing caused progressively greater reductions in infectious progeny formation as the temperature increased from 39 °C to 40.5 °C, with the strongest effect observed at 40.5 °C. No significant effect of ATC treatment was observed in the ntg strain. Cultures were also tested at 41 °C; however, no detectable IFUs were recovered from any strains under either ATC− or ATC+ conditions, and therefore these data are not shown in the graph. Data represent the mean ± SD of biological duplicates (39 °C) or triplicates (40, 40.5, and 41 °C).
