# Supplementary material for: A heat-induced HagF-HrcA regulatory loop promotes chlamydial fitness and infectious progeny yield under stress
Source: PLoS Pathog. 2026 Nov 4;22(11):e1014749. doi: 10.1371/journal.ppat.1014749 (PMC13750654; doi:10.1371/journal.ppat.1014749)
Supplement: Supplementary file 4 [file ppat.1014749.s005.pdf]

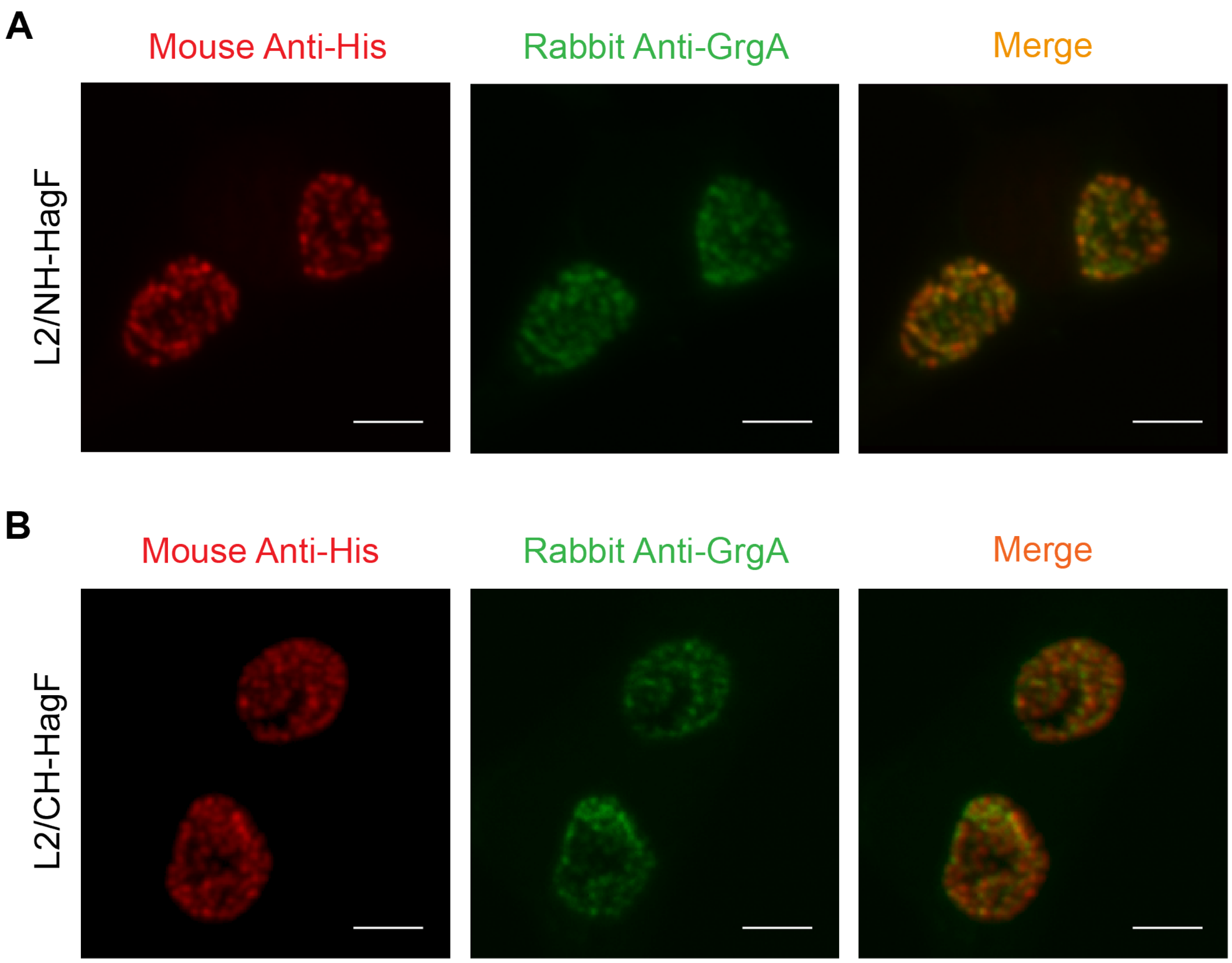

Fig. S5. HagF localizes inside chlamydial cells. HeLa 229 cells were infected with L2/NH-HagF (A) or L2/CH-HagF (B), fixed at 20 hpi, and immunostained. NH-HagF and CH-HagF were detected using a mouse anti-His antibody and TRITC-conjugated goat anti-mouse IgG. GrgA, a cytoplasmic chlamydial transcription factor, was detected using a rabbit anti-GrgA antibody and FITC-conjugated goat anti-rabbit IgG.
