# Supplementary material for: A heat-induced HagF-HrcA regulatory loop promotes chlamydial fitness and infectious progeny yield under stress
Source: PLoS Pathog. 2026 Nov 4;22(11):e1014749. doi: 10.1371/journal.ppat.1014749 (PMC13750654; doi:10.1371/journal.ppat.1014749)
Supplement: Supplementary file 5 [file ppat.1014749.s006.pdf]

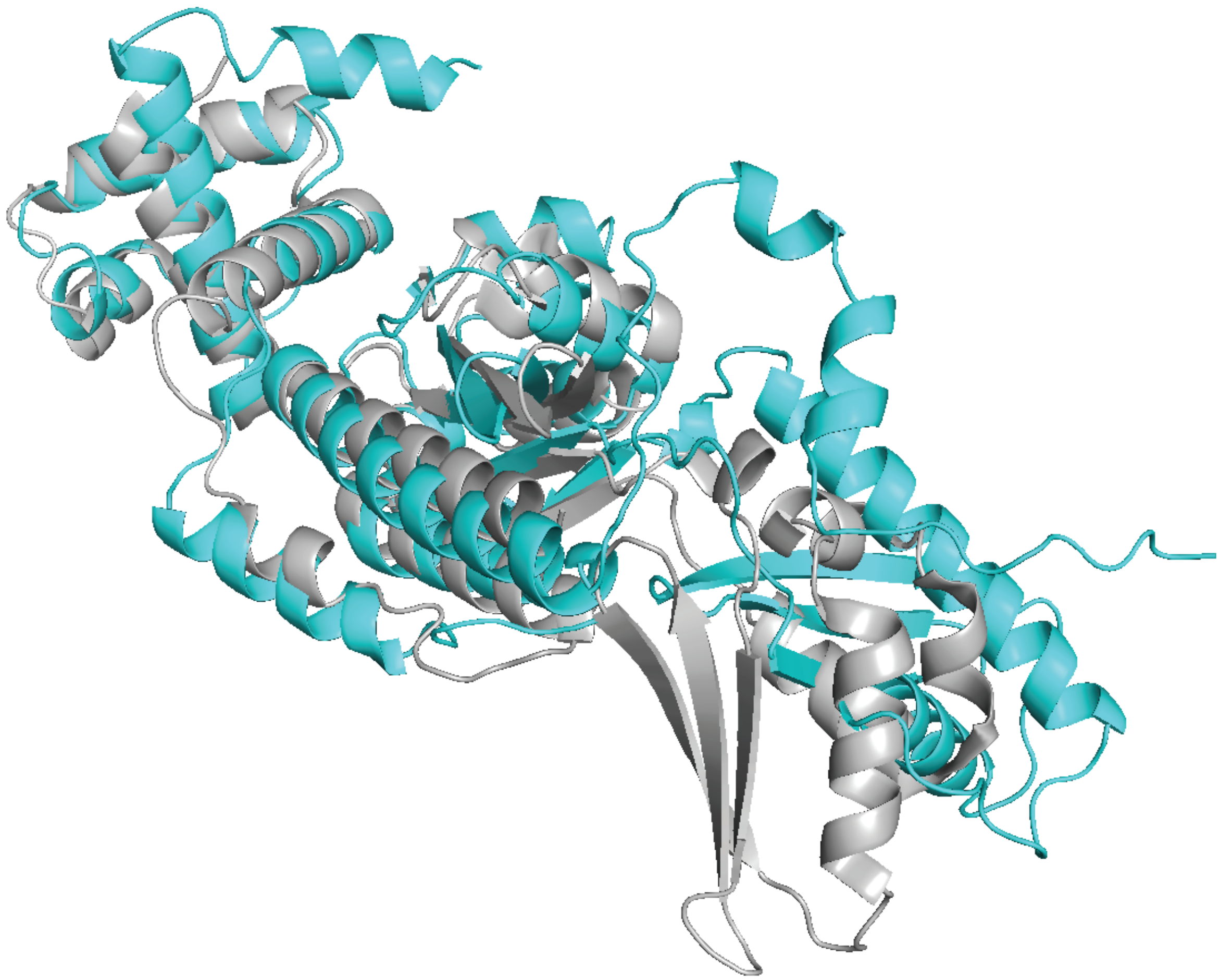

Fig. S6. Structural alignment of the AlphaFold-predicted model of *C. trachomatis* HrcA (cyan) with the experimentally determined *Thermotoga maritima* HrcA structure (1STZ, grey). The root mean square deviation (RMSD) of the alignment is 2.6 Å.
