# Supplementary material for: A heat-induced HagF-HrcA regulatory loop promotes chlamydial fitness and infectious progeny yield under stress
Source: PLoS Pathog. 2026 Nov 4;22(11):e1014749. doi: 10.1371/journal.ppat.1014749 (PMC13750654; doi:10.1371/journal.ppat.1014749)
Supplement: Supplementary file 6 [file ppat.1014749.s007.pdf]

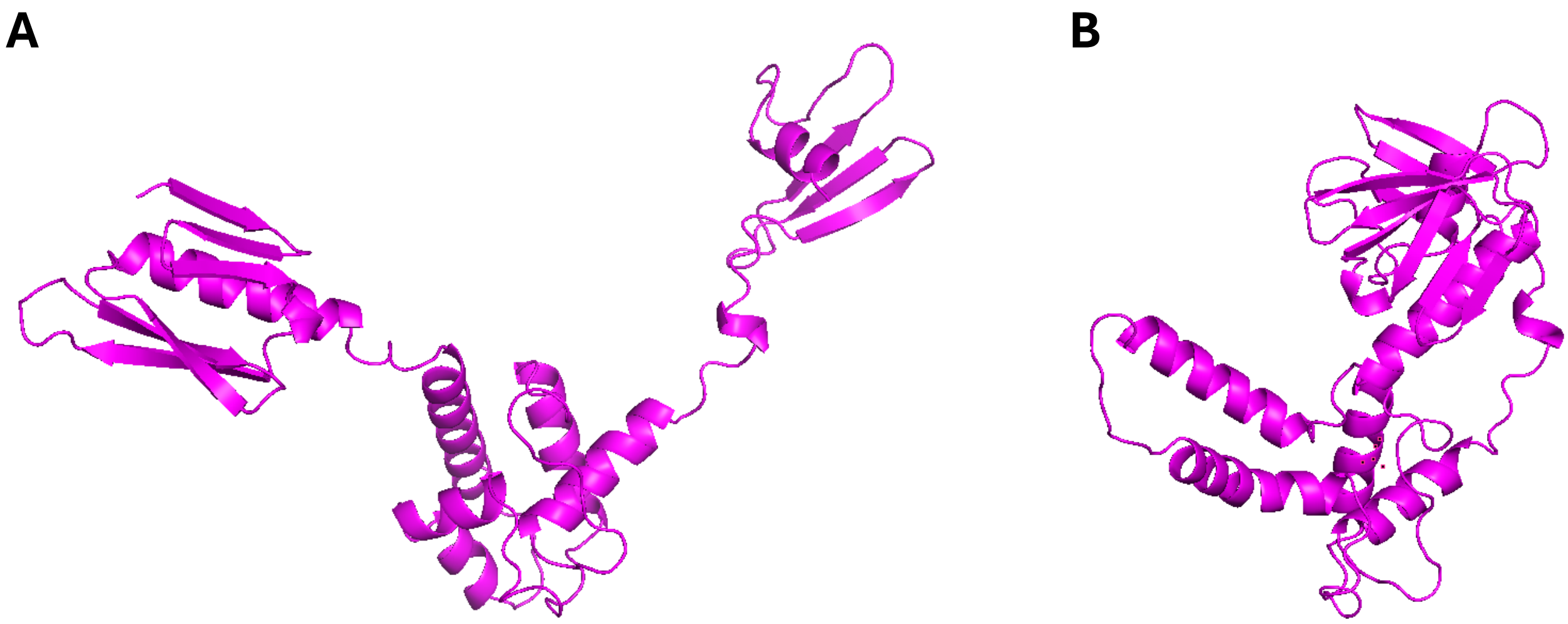

Fig. S7. AlphaFold3 models of HagF alone and in the HrcA-containing complex. (A) AlphaFold3-predicted structure of HagF modeled as a monomer. (B) HagF chain extracted from the AlphaFold3-predicted HagF–HrcA complex model. HagF appears more structurally ordered in the HrcA-containing model than when modeled alone.
