# Supplementary material for: A heat-induced HagF-HrcA regulatory loop promotes chlamydial fitness and infectious progeny yield under stress
Source: PLoS Pathog. 2026 Nov 4;22(11):e1014749. doi: 10.1371/journal.ppat.1014749 (PMC13750654; doi:10.1371/journal.ppat.1014749)
Supplement: Supplementary file 7 [file ppat.1014749.s008.pdf]

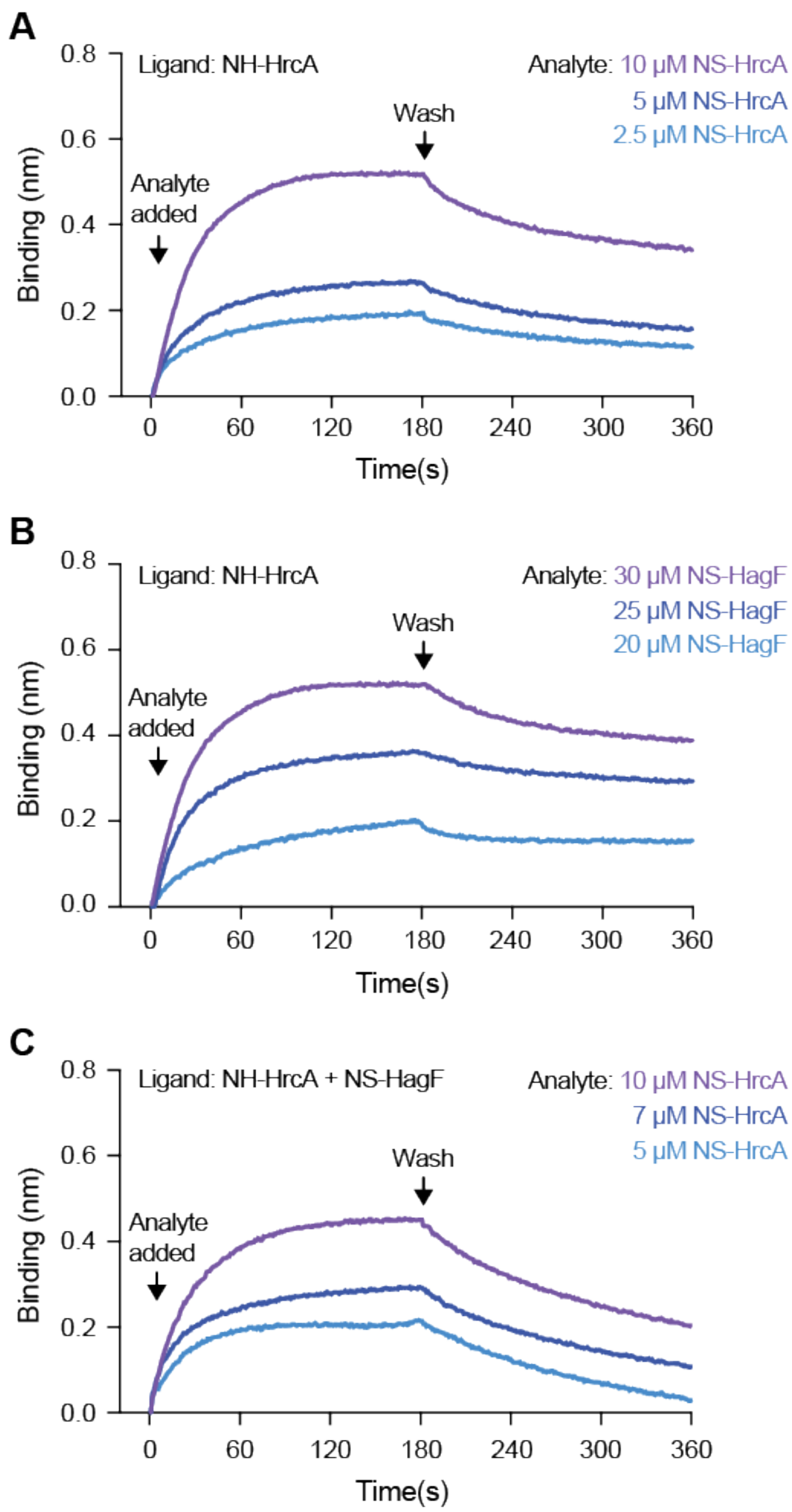

Fig. S8. Representative biolayer interferometry sensorgrams. Ni-NTA biosensors were loaded with NH-HrcA alone or NH-HrcA preincubated with NS-HagF as the immobilized ligand. Association was initiated by adding the indicated analyte, and dissociation was initiated by transferring the biosensor to BLI buffer at the wash step. (A) NS-HrcA binding to immobilized NH-HrcA. (B) NS-HagF binding to immobilized NH-HrcA. (C) NS-HrcA binding to immobilized NH-HrcA–NS-HagF complex. Analyte concentrations are indicated in each panel.
