# Supplementary material for: A heat-induced HagF-HrcA regulatory loop promotes chlamydial fitness and infectious progeny yield under stress
Source: PLoS Pathog. 2026 Nov 4;22(11):e1014749. doi: 10.1371/journal.ppat.1014749 (PMC13750654; doi:10.1371/journal.ppat.1014749)
Supplement: Supplementary file 8 [file ppat.1014749.s010.pdf]

Table S1. HrcA residues involved in the predicted HrcA–HrcA interface in HrcA homodimer and 2HagF–2HrcA heterotetramer models. Interface residues were identified from AlphaFold3-predicted HrcA–HrcA homodimer and 2HagF–2HrcA heterotetramer models. Residue counts refer to HrcA residues participating in the predicted HrcA–HrcA interface. In the 2HagF–2HrcA model, 42 of 43 HrcA interface residues overlap with the predicted HrcA homodimer interface.

HrcA homodimer				2HagF-2HrcA tetramer			
HrcA1-to-HrcA2		HrcA2-to-HrcA1		HrcA1-To-HrcA2		HrcA2-To-HrcA1	
Position	Residue	Position	Residue	Position	Residue	Position	Residue
11	ARG	11	ARG	119	LYS	119	LYS
14	LYS	14	LYS	122	GLN	122	GLN
17	SER	17	SER	123	LYS	123	LYS
18	LYS	18	LYS	126	GLU	126	GLU
21	TYR	21	TYR	150	ASN	150	ASN
24	LEU	24	LEU	152	GLN	152	GLN
25	MET	25	MET	154	VAL	154	VAL
32	GLU	32	GLU	155	ALA	155	ALA
33	SER	46	TYR	156	ILE	156	ILE
46	TYR	49	ASP	157	ASP	157	ASP
49	ASP	70	LYS	158	ASP	158	ASP
50	LEU	72	HIS	159	GLN	159	GLN
70	LYS	73	ILE	160	ARG	160	ARG
72	HIS	86	TYR	162	VAL	162	VAL
73	ILE	89	ASP	164	VAL	164	VAL
89	ASP	90	HIS	166	SER	166	SER
90	HIS	91	ASN	170	GLY	170	GLY
91	ASN	92	ALA	171	GLN	171	GLN
92	ALA	94	PHE	172	VAL	172	VAL
94	PHE	98	GLU	174	THR	174	THR
98	GLU	101	LEU	176	VAL	176	VAL
102	ALA	102	ALA	178	TRP	178	TRP
105	GLN	105	GLN	221	ASN	221	ASN
119	LYS	119	LYS	222	GLU	222	GLU
122	GLN	122	GLN	225	VAL	225	VAL
123	LYS	123	LYS	226	ARG	226	ARG
126	GLU	126	GLU	229	THR	229	THR
133	GLN	133	GLN	230	ARG	230	ARG
142	ARG	142	ARG	233	HIS	233	HIS
150	ASN	150	ASN	241	GLN	241	GLN
152	GLN	152	GLN	242	THR	242	THR
154	VAL	154	VAL	243	GLY	243	GLY
155	ALA	155	ALA	244	LEU	244	LEU
156	ILE	156	ILE	245	SER	245	SER
157	ASP	157	ASP	246	ARG	246	ARG
158	ASP	158	ASP	248	LEU	248	LEU
159	GLN	159	GLN	260	ALA	260	ALA

160	ARG	160	ARG	263	LEU	263	LEU
162	VAL	162	VAL	264	ALA	264	ALA
164	VAL	164	VAL	267	GLU	267	GLU
166	SER	166	SER	268	ASN	268	ASN
170	GLY	170	GLY	271	HIS	271	HIS
171	GLN	171	GLN	366	THR	366	THR
172	VAL	172	VAL				
174	THR	174	THR				
175	ASP	175	ASP				
176	VAL	176	VAL				
178	TRP	178	TRP				
218	VAL	218	VAL				
221	ASN	221	ASN				
222	GLU	222	GLU				
225	VAL	225	VAL				
226	ARG	226	ARG				
228	LEU	228	LEU				
229	THR	229	THR				
230	ARG	230	ARG				
232	CYS	232	CYS				
233	HIS	233	HIS				
235	SER	235	SER				
241	GLN	241	GLN				
242	THR	242	THR				
243	GLY	243	GLY				
244	LEU	244	LEU				
245	SER	245	SER				
246	ARG	246	ARG				
248	LEU	248	LEU				
249	LYS	249	LYS				
251	GLU	251	GLU				
253	PHE	253	PHE				
254	LYS	254	LYS				
255	ASP	255	ASP				
256	PRO	256	PRO				
257	GLU	257	GLU				
259	LEU	259	LEU				
260	ALA	260	ALA				
261	GLN	261	GLN				
263	LEU	263	LEU				
264	ALA	264	ALA				
266	PHE	266	PHE				
267	GLU	267	GLU				
268	ASN	268	ASN				
270	LYS	270	LYS				
299	ASN	299	ASN				
332	GLN	332	GLN				

365	PRO	365	PRO
366	THR	366	THR
367	ASP	367	ASP
368	PRO	368	PRO
373	ARG	373	ARG
374	PRO	374	PRO
375	ALA	375	ALA
378	THR	378	THR
385	LEU	385	LEU
386	LEU	386	LEU
388	ALA	388	ALA
389	LYS	389	LYS
390	GLU	390	GLU
