# Supplementary material for: A heat-induced HagF-HrcA regulatory loop promotes chlamydial fitness and infectious progeny yield under stress
Source: PLoS Pathog. 2026 Nov 4;22(11):e1014749. doi: 10.1371/journal.ppat.1014749 (PMC13750654; doi:10.1371/journal.ppat.1014749)
Supplement: Supplementary file 9 [file ppat.1014749.s012.pdf]

Table S3. qPCR and RT-qPCR primers

Name	Sequence	Utility
grgA-F	GCCGTTTTTACTGCCAGCAT	chromosomal qPCR
grgA-R	ACTATTGGAGCCACCTTCGG	chromosomal qPCR
23S rRNA-F	AGATAGACAGCGGGGGCTAA	RT-qPCR
23S rRNA-R	GGTGAGCTGTTACGCACTCT	RT-qPCR
hagF-F	CCACAGCGCCTCAGAAAAAC	RT-qPCR
hagF-R	TCGAACCAGCTCCTCCTAGT	RT-qPCR
dCas12-F	ATGGAACCGTCGTTGAGCTT	RT-qPCR
dCas12-R	AGGGTCGGCATTTGGAAGTT	RT-qPCR
hrcA-F	CGCTCAAGGTCTCGCTTTCT	qRT-PCR
hrcA-R	TACTGCACACGAAGGATCGG	RT-qPCR
grpE-F	TGGGATTCGCTTCTCAAACCT	RT-qPCR
grpE-R	AGCTTCATGCAGGTACGGATT	RT-qPCR
dnaK-F	CTGCAAAACGTCAGGCAGTA	RT-qPCR
dnaK-R	ACATCAAAGACCGCATCTCC	RT-qPCR
groES-F	GTCAGATCAAGCAACGACCC	RT-qPCR
groES-R	AATGATTCCGCCTCTTGCAGT	RT-qPCR
groEL-F	CCAGCAAAACTGCTGACAAA	RT-qPCR
groEL-R	TGCTCCAGCTGTTACATTGC	RT-qPCR
KP1-F	CGGTGGAGACGGTTTTCTTA	ChIP-qPCR
KP1-R	TCGCAGTTGGGACATTTCTA	ChIP-qPCR
EP-F	CGGAAGCTGGAAGAACTAGC	ChIP-qPCR
EP-R	GAACGTTCAGAGGAAGAACGA	ChIP-qPCR
KP2-F	CGCGTAGCCAAAGTGAAAGT	ChIP-qPCR
KP2-R	TTTTCGCTCATATTGAACACC	ChIP-qPCR
EP2-F	CTTCTCCCAGAATATGCTTGAT	ChIP-qPCR
EP2-R	AGACAAACCCTATTCCTCTATCAG	ChIP-qPCR
271P-F	AGAAGTCGGGAGAGAGGGTTT	ChIP-qPCR
271P-R	TCGCTTTCCTCGGCATTGT	ChIP-qPCR
