# Supplementary material for: A heat-induced HagF-HrcA regulatory loop promotes chlamydial fitness and infectious progeny yield under stress
Source: PLoS Pathog. 2026 Nov 4;22(11):e1014749. doi: 10.1371/journal.ppat.1014749 (PMC13750654; doi:10.1371/journal.ppat.1014749)
Supplement: Supplementary file 10 [file ppat.1014749.s014.pdf]

Dataset S2. Uncropped and Unadjusted Blot Images

Figure 3A

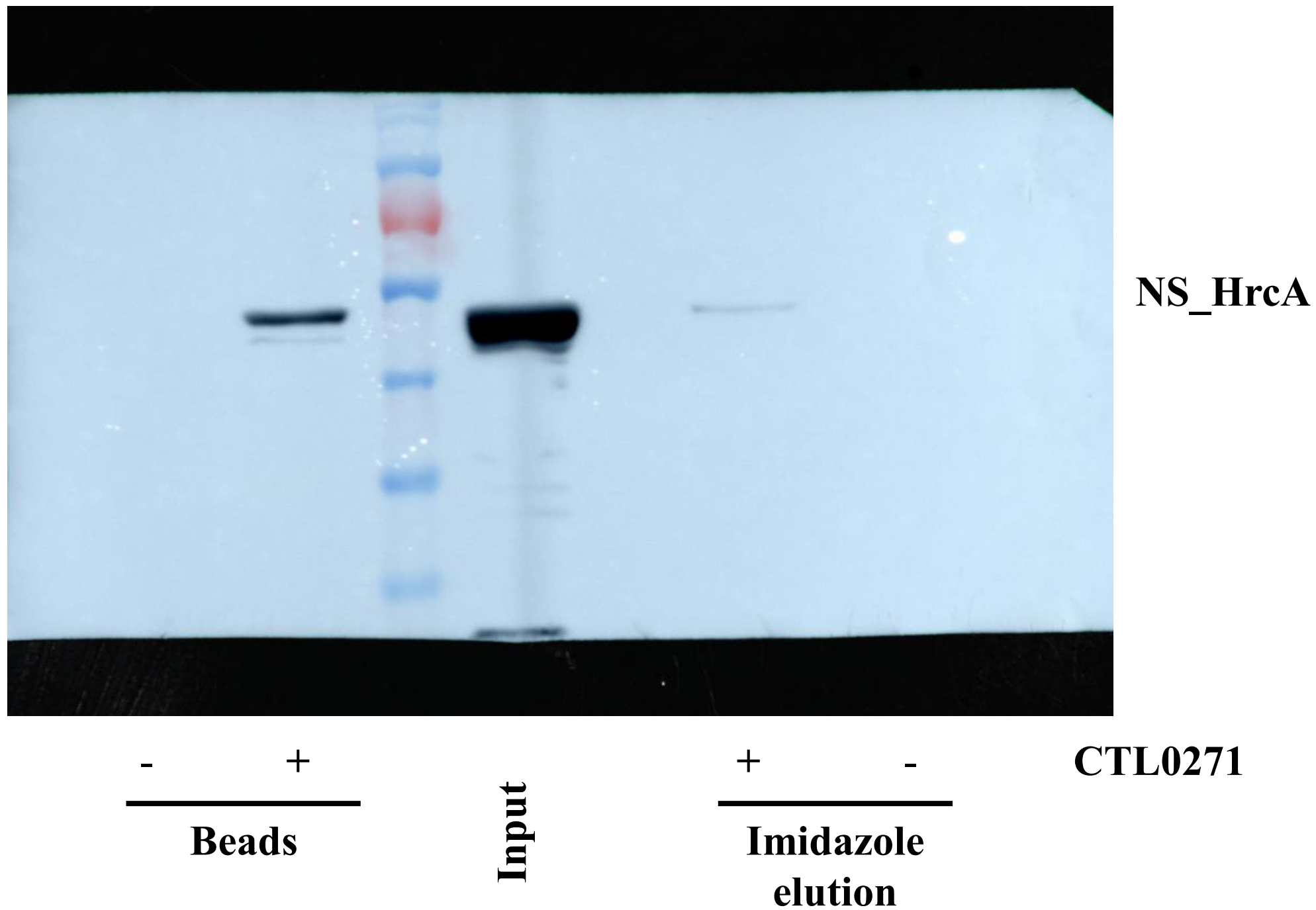

Figure 3 B

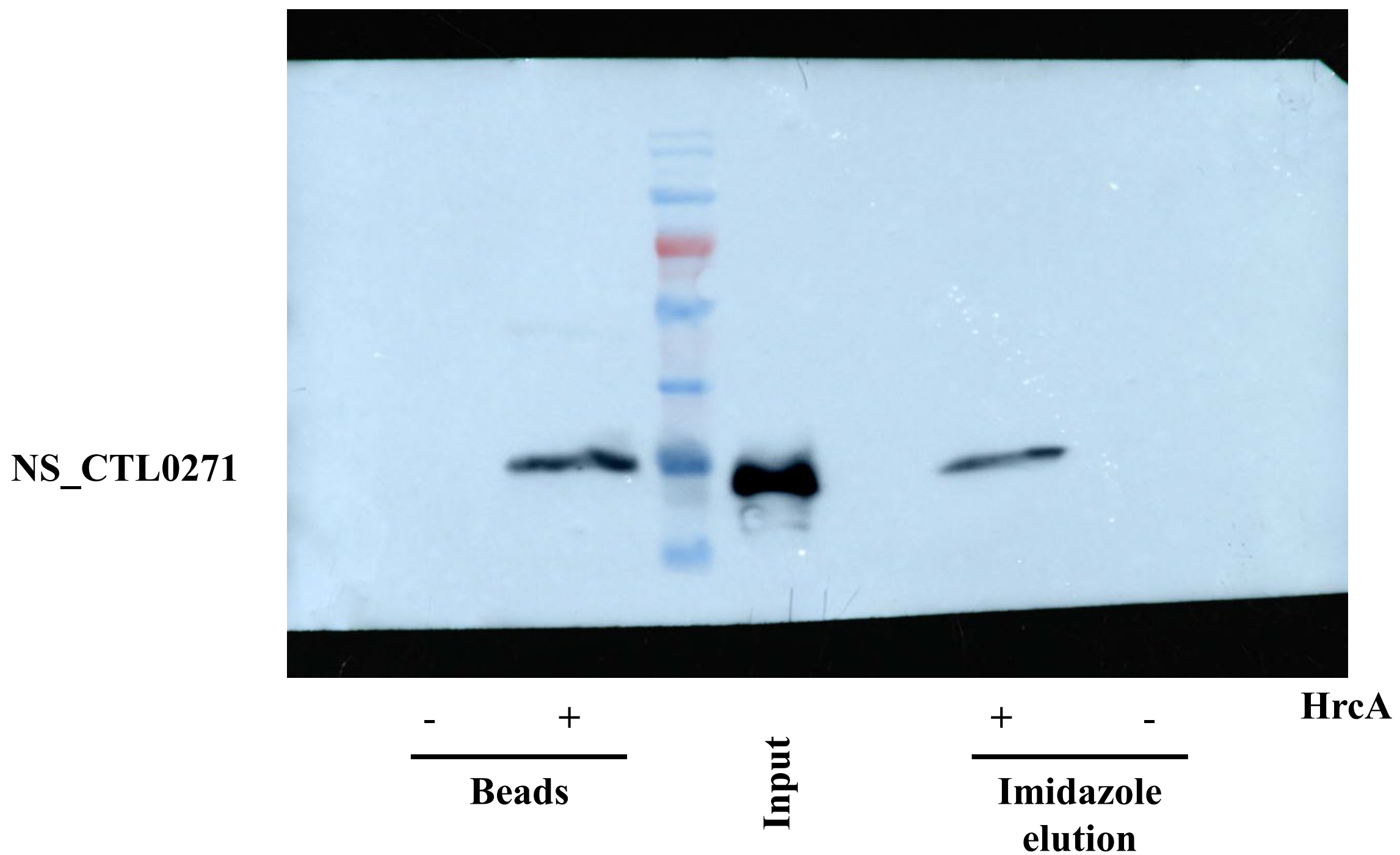

Figure 3 panel G and H

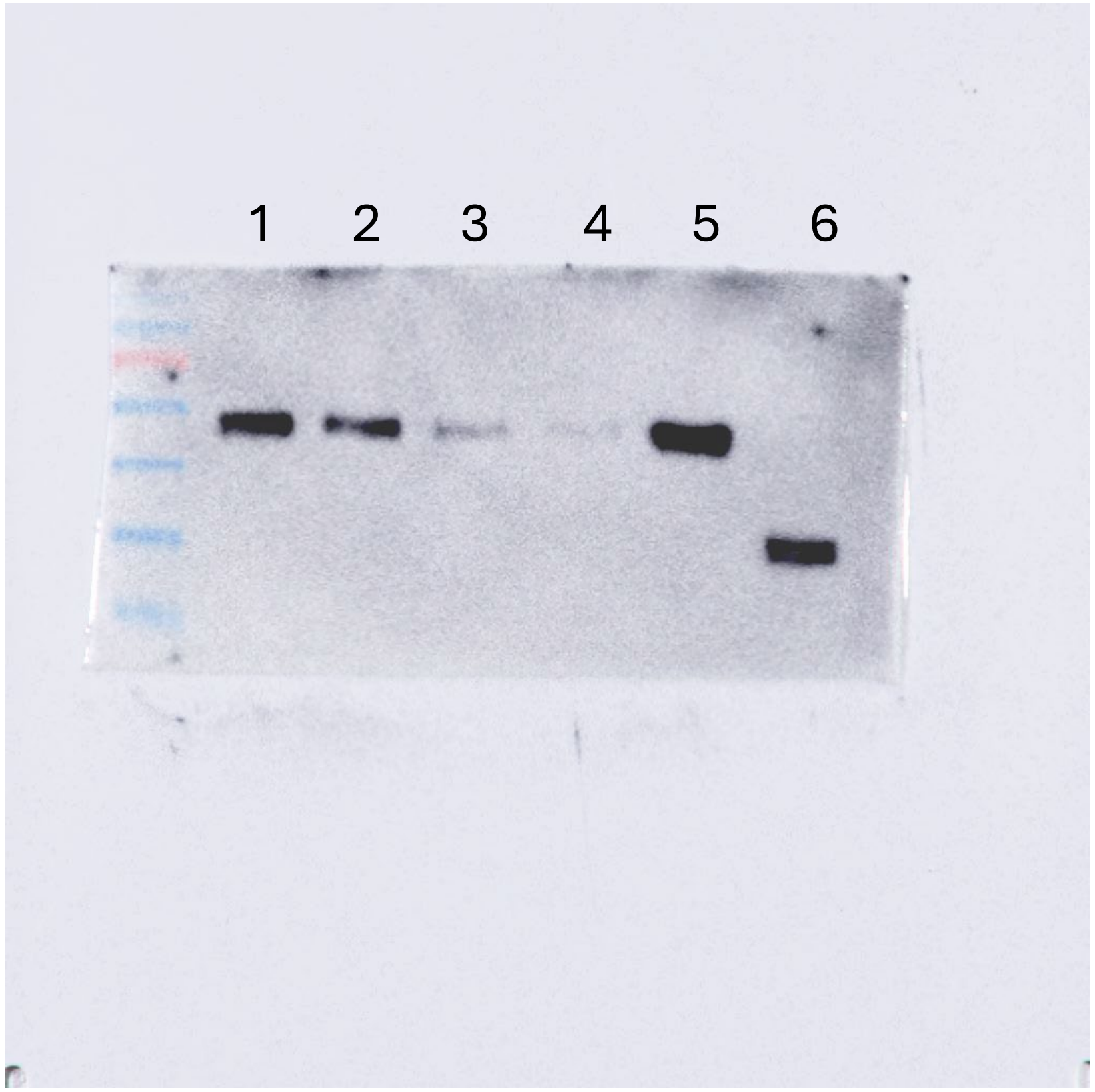

Lane1: NH-HrcA only

Lane2: NH-HrcA + 2x NH-HagF

Lane3: NH-HrcA + 4x NH-HagF

Lane4:NH-HrcA + 8x NH-HagF

Lane5:NH-HrcA (input)

Lane6:NH-HagF (input)

Figure 3 panel H

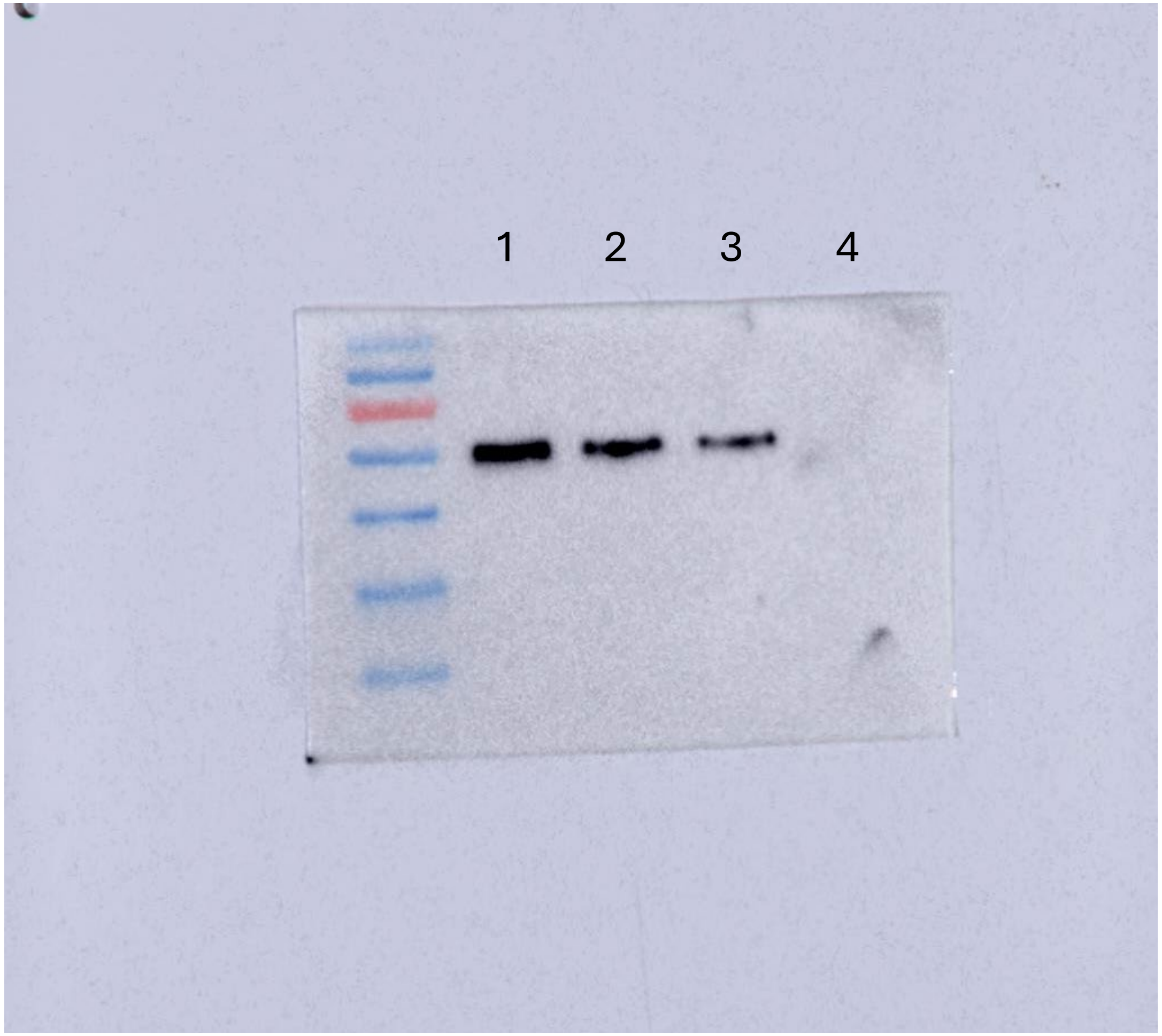

Lane1: NH-HrcA only

Lane2: NH-HrcA + 2x NH-HagF

Lane3: NH-HrcA + 4x NH-HagF

Lane4:NH-HrcA + 8x NH-HagF

Figure 3 panel H

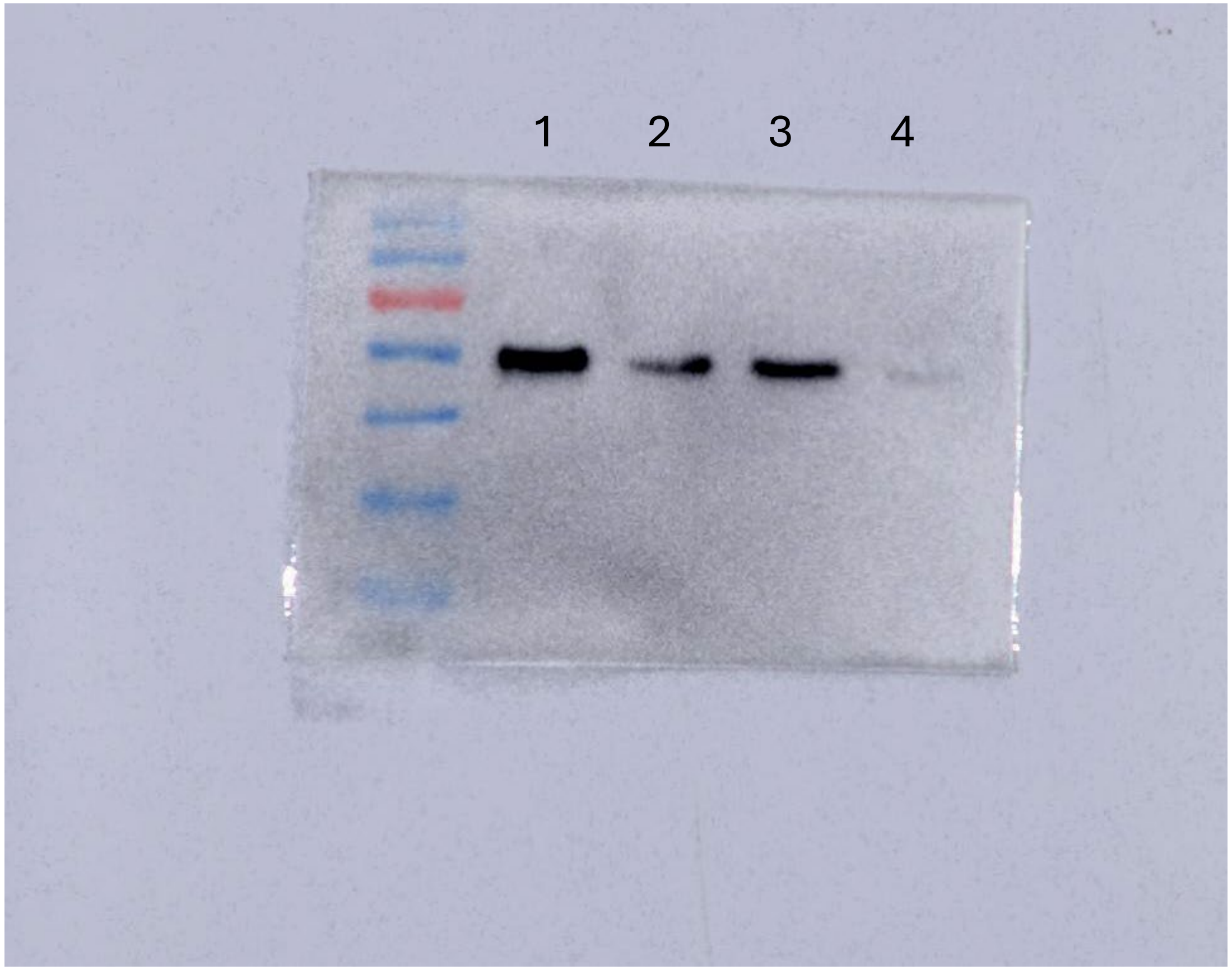

Lane1: NH-HrcA only

Lane2: NH-HrcA + 2x NH-HagF

Lane3: NH-HrcA + 4x NH-HagF

Lane4:NH-HrcA + 8x NH-HagF

Figure 3 panel H

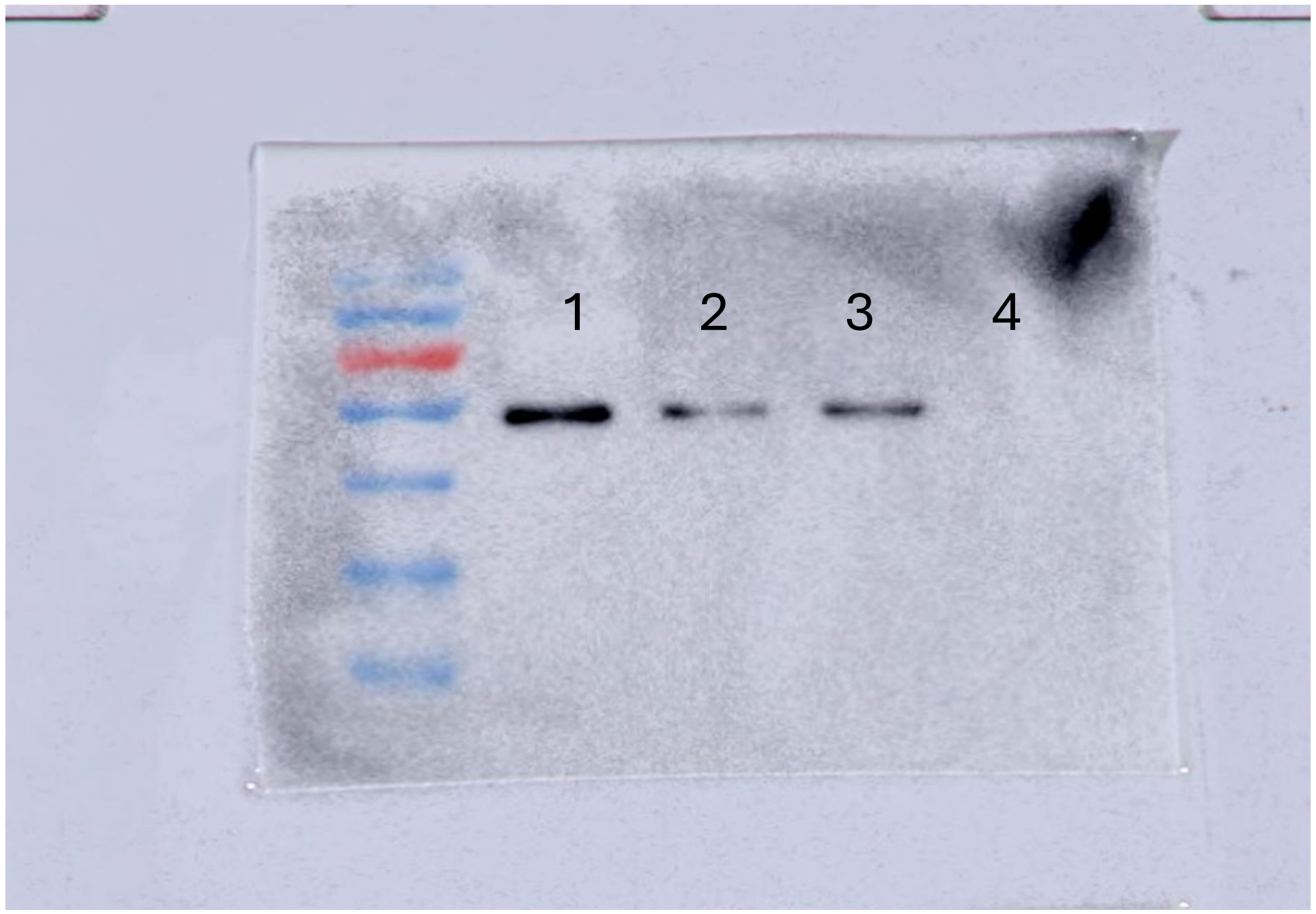

Lane1: NH-HrcA only

Lane2: NH-HrcA + 2x NH-HagF

Lane3: NH-HrcA + 4x NH-HagF

Lane4:NH-HrcA + 8x NH-HagF
