# Supplementary material for: A heat-induced HagF-HrcA regulatory loop promotes chlamydial fitness and infectious progeny yield under stress
Source: PLoS Pathog. 2026 Nov 4;22(11):e1014749. doi: 10.1371/journal.ppat.1014749 (PMC13750654; doi:10.1371/journal.ppat.1014749)
Supplement: Supplementary file 11 [file ppat.1014749.s016.pdf]

Response to Reviewers

We thank the editors and reviewers for their careful evaluation of our manuscript and for their constructive comments. In response to the comments, we have substantially revised the manuscript. We believe that these changes have significantly strengthened our work.

Below, we first present a brief summary of major changes and then a point-by-point response to specific review comments.

Summary of major changes

1. Added biolayer interferometry (BLI) analyses to provide quantitative biophysical support for HagF-HrcA association and for the effect of HagF on HrcA self-association. These data are presented in Fig. 3E, Table S2, and Fig. S8.
2. Revised the AlphaFold3 modeling section to focus on the predicted 2HagF-2HrcA heterotetramer and the reduced HrcA-HrcA interface in this model, now shown in Fig. 3C and 3D and detailed in Table S1.
3. Revised the mechanistic interpretation to avoid overstatement. The revised manuscript concludes that HagF directly interacts with HrcA, weakens HrcA self-association, and reduces HrcA association with CIRCE DNA, while acknowledging that the precise mechanism may involve weakened self-association, steric or allosteric inhibition of DNA binding, or a combination of these mechanisms.
4. Added RT-qPCR data showing that *hagF* knockdown reduces expression of the late, EB-enriched genes *omcA* and *omcB* at 40.5 °C. These data are now presented in Fig. 2I.
5. Replaced or qualified instances of 'EB formation' with 'infectious progeny yield' or related terminology (where appropriate) and explicitly discussed that IFU assays reflect both EB generation and EB infectivity.
6. Added a specificity control showing that overexpression of a σ^{66}-binding-deficient GrgA variant does not induce HrcA-regulated heat shock genes. These data are presented in Fig. S9.
7. Added a new supplemental figure showing the previously reported *hagF* transcriptional start site (TSS) and the predicted -35 and -10 promoter elements, each differing from the chlamydial σ66 consensus by only one nucleotide. This is presented in Fig. S1.
8. Added temperature-titration data showing that *hagF* knockdown reduces infectious progeny yield from 39 °C to 40.5 °C, with the strongest effect at 40.5 °C, and clarified why 45 °C was used for short-term heat shock induction experiments. These data are presented in Fig. S4.
9. Expanded the Introduction and Discussion to better justify the relevance of moderate heat stress and to distinguish our study from Kozusnik et al.

Response to Editors' comments

Comment 1

Additional experimental support is needed for the proposed HagF-HrcA interaction and its mechanistic consequences. While the current data are suggestive, the reviewers felt that the evidence is not yet sufficient to support some of the mechanistic claims presented in the manuscript.

Response

To further define the HagF–HrcA interaction and its mechanistic consequences, we have added new biophysical and structural analyses.

First, we have added BLI assays to quantify the binding relationships among HrcA-HrcA, HrcA-HagF, and HrcA-HagF/HrcA. NH-HrcA bound NS-HagF with an apparent affinity comparable to NH-HrcA binding to NS-HrcA, supporting direct HagF-HrcA association. Importantly, when NH-HrcA was preincubated with NS-HagF before immobilization, the resulting NH-HrcA-NS-HagF complex bound NS-HrcA with significantly reduced apparent affinity relative to the NH-HrcA/NS-HrcA interaction. These added BLI data are presented in Fig. 3E and Table S2, with representative BLI sensorgrams shown in Fig. S8.

Second, we have revised the AlphaFold3 modeling analysis. The revised Fig. 3C now shows a predicted 2HagF-2HrcA heterotetramer, and Fig. 3D compares the predicted HrcA-HrcA interface in the HrcA homodimer and 2HagF-2HrcA heterotetramer models. This analysis indicates that the HagF-containing model retains an HrcA-HrcA interface but with more than 50% fewer predicted interface residues, providing a structural model consistent with the BLI result that HagF weakens, rather than completely abolishes, HrcA self-association.

Third, based on the newly obtained BLI data, structural model, and the CIRCE DNA-binding data, we have revised the mechanistic language throughout the manuscript. We no longer state that HagF-mediated disruption of HrcA dimerization has been definitively demonstrated. Instead, we conclude that HagF interacts with HrcA, weakens HrcA self-association, and reduces HrcA association with CIRCE DNA. We present weakened HrcA self-association, allosteric inhibition, steric inhibition of DNA binding, or a combination of these mechanisms as possible explanations. The revised Discussion now states:

"Mechanistically, HagF antagonizes HrcA by reducing its ability to associate with CIRCE DNA. Reciprocal pulldown assays support a direct association between HagF and HrcA. AlphaFold3 modeling further predicts that HagF and HrcA can form a 2HagF-2HrcA heterotetramer in which the HrcA-HrcA interface is reduced relative to the predicted HrcA homodimer. Consistent with this structural model, BLI analysis showed that HrcA bound HagF with an apparent affinity comparable to HrcA self-association, whereas a preformed HrcA-HagF complex bound additional HrcA with reduced apparent affinity, reflected by decreased apparent k_{on} and increased apparent k_{off}. These data indicate that HagF weakens, rather than abolishes, HrcA self-association. Consistent with this mechanism, HagF reduced the ability of HrcA to associate with CIRCE DNA *in vitro*. Thus, HagF functions as a direct antagonist of HrcA. The precise mechanism by which HagF reduces HrcA-CIRCE association remains to be determined and may involve weakened HrcA self-association, allosteric or steric inhibition of DNA binding, or a combination of these mechanisms."

Comment 2

The causal relationship between HagF function and EB formation remains insufficiently demonstrated. Please provide additional experimental support for this conclusion or revise the interpretation accordingly. The current data more directly support effects on EB viability rather than conclusively demonstrating a halt in developmental progression.

Response

We have revised the manuscript to use more precise terminology. Specifically, where the data are based on IFU assays, we now generally use "infectious progeny yield," "infectious EB yield," or "IFU yield," rather than "EB formation."

To provide additional support for a developmental effect, we have added new RT-qPCR data measuring expression of the late, EB-enriched genes *omcA* and *omcB* at 40.5 °C. ATC treatment did not reduce *omcA* or *omcB* transcript levels in the non-targeting control strain, whereas *hagF* silencing significantly decreased both transcripts in L2/dC12-hagF-g1 and L2/dC12-hagF-g2. These data are now included as Fig. 2I.

We have also revised the Results and Discussion to state that the late-gene expression data are consistent with impaired late developmental progression contributing to the reduced IFU yield, while acknowledging that HagF may also help preserve EB infectivity under heat stress. The revised Discussion states:

"However, because late-stage IFU assay results reflect both the generation of infectious EBs and their ability to remain infectious, HagF may promote infectious progeny yield by supporting EB formation, preserving EB infectivity under heat stress, or both."

Comment 3

The interpretation of gene expression changes observed upon HagF overexpression requires additional controls. Please address whether these effects could arise from nonspecific consequences of protein overexpression.

Response

We have added a specificity control showing that overexpression of a σ^{66}-binding-deficient GrgA variant does not increase expression of HrcA-regulated heat shock genes. These data are now presented in Fig. S9. In the revised Results, we state:

"In contrast, ATC-induced overexpression of a σ66-binding-deficient GrgA variant did not increase expression of these HrcA-regulated heat shock genes, as demonstrated by RT-qPCR analysis (Fig. S9) and RNA-seq analysis [51]. These control experiments argue against a nonspecific protein-overexpression effect and support the conclusion that HagF specifically inhibits HrcA-mediated transcriptional repression."

Comment 4

The reviewers raised concerns regarding the assignment of the putative -35 and CIRCE elements in the *ctl0271* promoter region. Please provide additional experimental evidence or clarification supporting these assignments.

Response

We have added a new Fig. S1 to clarify the promoter architecture upstream of *hagF*. This figure shows the intergenic region between *ctl0270* and *hagF*, the previously reported transcriptional start site, the predicted -35 and -10 elements, and the nearby CIRCE element. The predicted -35 and -10 elements

differ from the chlamydial σ66 consensus elements by only a single nucleotide each, with the unmatched nucleotides indicated.

The revised text now refers to Fig. S1 when discussing the *hagF* promoter region. We also revised the figure legend to clarify that the TSS was previously reported and that the -35 and -10 elements are predicted based on their spacing and close match to the consensus σ66 core promoter.

We note that our ChIP-qPCR data provide experimental support for HrcA occupancy at the *hagF* promoter region in *C. trachomatis*. We agree that footprinting would provide higher-resolution mapping of protected nucleotides, but the combined evidence of the CIRCE-like sequence, promoter architecture, previously mapped TSS, HrcA ChIP enrichment, HrcA-mediated repression, and heat-induced derepression supports assignment of this region as an HrcA-regulated promoter.

Comment 5

Please justify the physiological relevance of the heat shock conditions used in the study, especially 45 versus 40.5 °C.

Response

We have clarified the rationale for using different heat shock conditions in the study. The short-term 45 °C treatment served as a robust induction condition to identify heat-responsive genes and regulators, whereas functional *hagF* knockdown studies were carried out at 40.5 °C, a biologically informative heat shock condition that permits assessment of chlamydial growth and infectious progeny yield.

We have revised the manuscript to better explain the relevance of moderate thermal stress and to clarify the functional temperature range used for the *hagF* knockdown experiments. In the revised Introduction, we now explain that thermal stress can vary in magnitude and duration in host tissues and may impair, rather than completely arrest, the chlamydial developmental cycle. We further distinguish moderate thermal stress from stresses that impose nutrient limitation, antimicrobial damage, or strong immune restriction, emphasizing that moderate thermal stress may primarily challenge protein homeostasis and thereby make chaperone-mediated maintenance of development particularly important.

We also added temperature-titration data showing that *hagF* knockdown reduced infectious progeny yield from 39 to 40.5 °C, with the strongest effect at 40.5 °C, whereas no detectable IFUs were recovered from any strain at 41 °C, with or without ATC treatment. Together, these revisions support the use of 40.5 °C as a biologically informative heat shock condition for growth and infectious progeny assays.

Comment 6

In light of concerns regarding novelty, please more clearly define how the present study advances beyond prior reports.

Response

We have revised the Discussion to more clearly distinguish our findings from Kozusnik et al. Whereas Kozusnik et al. reported stress-induced expression of *ctl0271/hagF* homologues and proposed that

these genes are HrcA targets, our study identifies the encoded *C. trachomatis* protein HagF as a direct antagonist of HrcA. The revised Discussion states:

"Whereas Kozusnik et al. primarily positioned *wcw_0502/ctl0271* as a stress-induced, HrcA-regulated gene, our study demonstrates that the encoded *C. trachomatis* protein HagF functions as an HrcA antagonist, thereby creating a negative feedback loop that modulates chaperone gene expression and infectious progeny yield during heat stress."

Thus, our study advances beyond prior work by demonstrating the physiological function of HagF in *C. trachomatis*, establishing HagF-HrcA association using reciprocal pulldown and BLI (Fig. 3A, B, E; Table S2; Fig. S8), showing that HagF weakens HrcA self-association (Fig. 3E; Table S2), demonstrating that HagF reduces HrcA association with CIRCE DNA (Fig. 3F-H), identifying HagF-dependent modulation of HrcA target genes *in vivo* (Fig. 4 and Fig. S9), and linking this regulatory circuit to infectious progeny yield during heat stress (Fig. 2 and Fig. S4).

Response to Reviewer 1

Major comment 1

The causality between HagF-HrcA regulation and EB formation is not proven. A phenotype is shown whereby *hagF* knockdown reduces infectious EB at 40.5 °C, but this does not directly show it is caused by the HrcA regulon rather than another function. Additional experiments should be carried out to demonstrate this, or the stated conclusions should be weakened.

Response

We have revised the manuscript in two ways to address the causal relationship between HagF-HrcA regulation and EB formation.

First, we softened the interpretation throughout the manuscript. We now refer primarily to "infectious progeny yield" or related terminology when describing IFU assay results, rather than using "EB formation" as the sole interpretation. We also explicitly acknowledge in the Discussion that IFU assay results reflect both the generation of infectious EBs and their ability to remain infectious.

Second, we have added new RT-qPCR data showing that *hagF* knockdown at 40.5 °C significantly reduces expression of the late, EB-enriched genes *omcA* and *omcB*, whereas ATC treatment does not reduce these transcripts in the non-targeting control strain. These results are now presented in Fig. 2I and support the interpretation that impaired late developmental progression contributes to the reduced infectious progeny yield.

We also revised the Discussion to acknowledge that the physiological effects of HagF may arise, at least in part, through altered regulation of the HrcA-controlled chaperone system, without excluding additional HagF functions.

Major comment 2

The mechanistic claim about HagF disrupting HrcA dimerization is not directly proven. There is a reciprocal pulldown and AlphaFold modeling, but no direct evidence about HrcA dimerization. This could be done by SEC-MALS, ultracentrifugation, etc., and point mutagenesis to test the predicted interface.

Response

To address this concern experimentally, we have added BLI assays to test HagF–HrcA binding and the effect of HagF on HrcA self-association. These experiments showed that HrcA binds HagF with an apparent affinity comparable to HrcA self-association. Importantly, a preformed HrcA–HagF complex retained the ability to bind additional HrcA but did so with reduced apparent affinity, indicating that HagF weakens, rather than abolishes, HrcA self-association *in vitro*. These data are now presented in Fig. 3E and Table S2, with representative sensorgrams shown in Fig. S8.

We also revised the AlphaFold3 modeling section. The revised manuscript now presents a predicted 2HagF-2HrcA heterotetramer and compares the HrcA-HrcA interface in the HrcA homodimer and HagF-containing heterotetramer models. This analysis shows that the heterotetramer retains a substantially reduced HrcA-HrcA interface, providing a structural model consistent with the BLI data. These data are shown in Fig. 3C and 3D and detailed in Table S1.

We interpret these results conservatively. The revised manuscript concludes that HagF weakens HrcA self-association and reduces HrcA association with CIRCE DNA, while acknowledging that the precise mechanism may involve weakened HrcA self-association, allosteric or steric inhibition of DNA binding, or a combination of these mechanisms.

Major comment 3

ChIP data uses tagged and induced HrcA. Native promoter occupancy is not shown.

Response

Native HrcA ChIP would be a useful complementary experiment. However, the lack of a validated antibody suitable for efficient native HrcA ChIP in *C. trachomatis* makes this technically challenging. We therefore used a His-tagged HrcA construct to enable specific immunoprecipitation.

Importantly, this experiment included positive-control HrcA target promoters, KP1 and EP, which showed strong enrichment, and the *hagF* promoter was enriched to a comparable level. In addition, our conclusion that *hagF* is HrcA-regulated is supported by multiple independent lines of evidence: the presence of a CIRCE-like element upstream of *hagF*, the clarified σ^{66} promoter architecture shown in Fig. S1, HrcA occupancy by ChIP-qPCR, repression of *hagF* expression by HrcA overexpression, and heat-induced derepression of *hagF*. We have revised the text to present the ChIP data as one component of this combined evidence.

Minor comment 1

The title/discussion imply a broad stress phenotype but the actual data only looks at heat shock. Other stressors are not explored. The title/discussion should be adjusted to reflect this.

Response

We have revised the manuscript to focus the functional conclusions on heat stress. The title now refers to infectious progeny yield under stress, and throughout the Results and Discussion, we specify heat stress or heat-responsive regulation when referring to our experimental data. We retained broader stress-responsive language only where it refers to the HrcA system generally, prior studies, or broader conceptual implications.

Minor comment 2

There is no rescue experiment for the *hagF* knockdown experiments in Fig. 2. This would strengthen this experiment.

Response

A rescue experiment would further strengthen the genetic analysis. However, we did not pursue complementation because we were concerned that ectopic *hagF* expression could introduce an overexpression-associated phenotype. Consistent with this concern, our HagF overexpression experiments showed that unregulated HagF expression itself impairs chlamydial growth. To strengthen the specificity of the loss-of-function phenotype, we instead used two independent *hagF*-targeting guide RNAs (gRNAs) together with a non-targeting control strain. Both *hagF* gRNAs reduced *hagF* transcript levels and reduced infectious progeny yield under heat stress, whereas ATC treatment of the non-targeting control had no such effect. In addition, both *hagF* knockdown strains showed reduced expression of the late, EB-enriched genes *omcA* and *omcB* at 40.5 °C. The concordant knockdown, molecular, and infectious progeny phenotypes observed with two independent gRNAs make an off-target CRISPRi effect unlikely.

Minor comment 3

Could elaborate in the introduction about the physiological role of heat stress in Chlamydiae, presumably during fever.

Response

We have revised the Introduction to better discuss the relevance of thermal stress. We now explain that thermal stress may vary in magnitude and duration in host tissues and may impair, rather than completely arrest, chlamydial development. We also distinguish moderate thermal stress from stresses that impose nutrient limitation, antimicrobial damage, or strong immune restriction. This revision provides a clearer rationale for why chaperone-mediated maintenance of development may be particularly important during heat stress.

Minor comment 4

Fig. 1 legend should detail meaning of N9 and N5.

Response

We have revised the figure legend to clarify the nucleotide spacer designations.

Minor comment 5

Elaborate on whether there are other genes in the genome with predicted CIRCE elements, or are all listed in Fig. 1.

Response

We clarify that Fig. 1 focuses on the established HrcA-regulated promoters KP1 and EP, together with the newly identified *hagF* promoter. We do not intend Fig. 1 to represent a complete genome-wide prediction of all possible CIRCE-like sequences. Moreover, because functional CIRCE elements may

tolerate sequence variation, a genome-wide motif search would identify candidate sites but would not definitively exclude additional HrcA-regulated promoters. Our conclusion is therefore focused on *hagF*, which represents an additional experimentally supported HrcA-regulated gene based on promoter architecture, HrcA ChIP enrichment, HrcA-dependent repression, and heat-induced derepression.

Minor comment 6

Fig. 2A contains two panels. This is confusing and would be better as two separate panels.

Response

We have revised Fig. 2 so that dC12 expression and *hagF* knockdown measurements are presented as separate panels.

Reviewer 2

Comment 1, Figure 4

Given the authors' hypothesis, why do *groES* and *groEL* levels increase upon *hagF* overexpression but do not decrease upon *hagF* silencing? Could the enhanced expression of *groES* and *groEL* simply result from overexpressing any protein from an inducible plasmid?

Response

To address the possibility that the observed effects result from nonspecific protein overexpression, we have added a new control experiment using ATC-induced overexpression of a σ^{66}-binding-deficient GrgA variant. This variant is expressed from an inducible plasmid but does not increase the expression of HrcA-regulated heat shock genes. These data are now shown in Fig. S9. Thus, induction of HrcA target genes by HagF overexpression is not simply a generic consequence of overexpressing any plasmid-encoded protein.

We have also expanded the Discussion to address the differential effect on the *hrcA-grpE-dnaK* and *groES-groEL* operons during *hagF* knockdown. We propose that the KP1 CIRCE element, which is more highly conserved than the EP CIRCE element, may bind residual functional HrcA more effectively during heat stress. Under this model, the KP1-controlled *hrcA*-grpE-dnaK operon remains more dependent on HagF-mediated relief of HrcA repression, whereas the *groES-groEL* promoter may be sufficiently derepressed by heat-induced HrcA destabilization alone.

Comment 2, Figure 2

Given that IFU assays are actually EB viability assays, how do the authors distinguish whether differentiation is being halted or EB viability is simply being reduced under *hagF* depletion? A demonstration of transcriptional differences between control and *hagF* knockdowns for genes required for EB formation would allow a more convincing argument.

Response

We have added RT-qPCR analysis of the late, EB-enriched genes *omcA* and *omcB* under heat stress. ATC treatment did not reduce *omcA* or *omcB* expression in the non-targeting control strain, whereas *hagF* silencing significantly reduced both transcripts in L2/dC12-hagF-g1 and L2/dC12-hagF-g2. These data are now presented in Fig. 2I and support the interpretation that impaired late developmental progression contributes to the reduced infectious progeny yield.

We have also revised the manuscript wording to reflect that IFU assay results may be influenced by EB infectivity. Accordingly, we now generally use “infectious progeny yield” or related terminology when referring to IFU assay results, rather than presenting these assays as direct measurements of EB formation. In addition, we explicitly acknowledge in the Discussion that HagF may promote infectious progeny yield by supporting EB formation, preserving EB infectivity under heat stress, or both.

Minor comment: Lines 60-62

Please provide references for this statement. (In response to milder stress, some RBs may enter persistence while others continue to replicate slowly, eventually allowing EB production.)

Response

We have added references supporting this statement. These studies show that stressors such as heat shock and IFN-γ-mediated stress can affect chlamydial developmental progression and infectious progeny production in a severity- and duration-dependent manner.

Minor comment: Line 62

Generally, chlamydial persistence has been understood to act as a means of halting chlamydial development until an extracellular/intracellular stressor has passed, and only then does development continue. The concept of sustaining EB formation during an ongoing stressor is not entirely intuitive. The idea being that it would be disadvantageous for Chlamydia to continue its developmental cycle under conditions that would prove deleterious to its extracellular form. The authors should try to provide a better rationale for why this may differ for heat stress, when compared to several other stressors (such as high levels of interferon gamma, antibiotics, nutrient starvation, etc).

Response

We agree that strong or prolonged stress can halt chlamydial development and promote persistence. We have revised the Introduction to better distinguish this persistence framework from the moderate thermal-stress conditions used in our functional assays. The revised text now states:

"In response to milder stress, some RBs may enter persistence, whereas others continue to replicate slowly, resulting in reduced but detectable infectious progeny yield [19, 20]. This distinction is particularly relevant for thermal stress, which can vary in magnitude and duration in host tissues and may impair, rather than completely arrest, the developmental cycle. Unlike stresses that impose specific nutrient limitation, antimicrobial damage, or strong immune restriction, moderate thermal stress may primarily challenge protein homeostasis, making chaperone-mediated maintenance of development particularly important."

Figure 1 comment

Image resolution appears below 300 dpi.

Response

We have prepared revised high-resolution figure files for resubmission.

Figure 1 heat shock relevance

While 45 °C is convenient for demonstrating this pathway *in vitro*, this temperature range is seldom experienced in humans. Is there a more physiologically relevant stressor?

Response

As mentioned above, 45 °C is not intended to model a sustained physiological temperature encountered during infection. Rather, the short-term 45 °C treatment in Fig. 1 was used as an induction condition to robustly identify heat-responsive genes and regulators. Therefore, we performed the functional *hagF* knockdown experiments at 40.5 °C, which is a more biologically informative heat shock condition that still permits assessment of chlamydial growth and infectious progeny yield.

To further address this point, we have added temperature-titration data showing that *hagF* knockdown reduced infectious progeny yield at 39, 40, and 40.5 °C, with the strongest effect at 40.5 °C; no detectable IFUs were recovered from any strain at 41 °C, with or without ATC treatment. These data are presented in Fig. S4 and show that the *hagF* knockdown phenotype occurs across a moderate heat shock range, not only under the 45 °C induction condition.

We have also revised the Introduction to better explain why moderate thermal stress may be relevant to chlamydial development. The revised text states:

"This distinction is particularly relevant for thermal stress, which can vary in magnitude and duration in host tissues and may impair, rather than completely arrest, the developmental cycle. Unlike stresses that impose specific nutrient limitation, antimicrobial damage, or strong immune restriction, moderate thermal stress may primarily challenge protein homeostasis, making chaperone-mediated maintenance of development particularly important."

Figure 2 comment

Why is 40.5 °C used here while 45 °C was used in Figure 1?

Response

As noted above, the two temperatures were used for different purposes. The short-term 45 °C treatment in Fig. 1 was used as an induction condition to robustly identify heat-responsive genes and regulators, whereas 40.5 °C was used for growth and infectious progeny assays because it permits measurable chlamydial development under a biologically informative heat shock condition. We have clarified this distinction in the revised manuscript and have also added temperature-titration data showing that *hagF* knockdown reduced infectious progeny yield from 39 °C to 40.5 °C, with the strongest effect at 40.5 °C (Fig. S4).

Figure 2 comment

Why were EB yields measured at different time points for different temperatures?

Response

Infectious progeny yields were measured at 30 hpi for 37 °C cultures and at 40 hpi for 40.5 °C cultures to account for slower chlamydial development at elevated temperature. This has been clarified in the revised figure legend.

Figure 2 comment

To eliminate confounding variables, it would help to demonstrate that cellular autophagy levels in infected cells grown at 40.5 °C are similar across all three chlamydial strains under ATC induction.

Response

Autophagy could be an interesting host-cell readout under heat stress. However, we did not add autophagy analysis because the experimental design already includes controls that address the key interpretation. All strains were grown under the same host-cell, temperature, and ATC conditions, and ATC treatment of the non-targeting control strain did not reduce genome replication or infectious progeny yield at 40.5 °C. In addition, *hagF* knockdown reduced IFU yield without significantly reducing genome replication kinetics, arguing against generalized host-cell toxicity or a broad growth defect as the sole explanation. We have revised the manuscript to interpret the phenotype more conservatively as an effect on infectious progeny yield and late developmental gene expression.

Lines 202-204

The results suggest fewer viable EBs when *hagF* is silenced. To demonstrate a reduction in EB formation, the authors would need to show fewer EBs or altered expression of EB-formation related genes.

Response

We agree. We have added RT-qPCR analysis of the late, EB-enriched genes *omcA* and *omcB*. These genes were significantly reduced by *hagF* knockdown at 40.5 °C but not by ATC treatment in the non-targeting control strain. These data are now shown in Fig. 2I. We also revised the language throughout the manuscript to avoid implying that IFU assays directly measure EB formation and to reflect the interpretation that these assays report infectious progeny yield.

Lines 210-213

The resolution does not eliminate the possibility that HagF could also be associated with the bacterial surface. A surface marker and high-resolution image would be needed to rule this out.

Response

We agree. We have revised the text, which now reads:

"These observations support the presence of HagF within chlamydiae and are consistent with substantial cytoplasmic localization, although they do not exclude the possibility that a fraction of HagF may also associate with the bacterial surface."

Figure 5 comment

Why is there a reduction in genome counts in addition to a reduction in viable EB counts when HagF is overexpressed at 37 °C?

Response

We interpret this result as evidence that unregulated HagF expression impairs overall chlamydial growth at 37 °C. Under conditions that impair chlamydial growth, reduced genome accumulation and reduced infectious progeny yield often occur together. Thus, the decrease in genome copies reflects slower bacterial replication/growth, whereas the decrease in IFUs reflects reduced infectious progeny yield.

Figure 5 comment

Does this trend hold when comparing growth under native/inducing conditions at 40.5 °C?

Response

We tested the effect of HagF overexpression at elevated temperature and did not observe a protective effect. Instead, forced HagF expression further impaired growth under heat stress. Because this result introduces an additional dosage-dependent interpretation and is not required for the core loss-of-function conclusion, we did not incorporate it into the manuscript.

Lines 388-390

The authors' readout is viable EBs, not "EB formation."

Response

We agree and revised the manuscript accordingly. We now use "infectious progeny yield" when discussing IFU assay results. We only discuss EB formation or late developmental progression in the context of the new *omcA* and *omcB* expression data, and we explicitly acknowledge that IFU assay results reflect both EB generation and maintenance of EB infectivity.

Lines 398-400

The argument about febrile hosts and transmission is not persuasive. The authors should incorporate stronger pathogenic/immunological context or consider that this pathway may be used more broadly during stress responses.

Response

We have revised the manuscript to avoid suggesting a role during fever. We now frame the pathway as one that supports chlamydial fitness and developmental competence under restrictive conditions. We also note that HagF orthologs are upregulated by heat shock and other stressors in environmental

chlamydiae, suggesting that this regulatory strategy may be relevant to broader stress-responsive adaptation.

Response to Reviewer 3

General comment

We respectfully disagree that the novelty of the study is limited to confirming heat-induced expression or HrcA-dependent regulation of *ctl0271*. Kozusnik et al. primarily positioned *wcw_0502/ctl0271* homologues as stress-induced, HrcA-regulated genes. In contrast, our study demonstrates that the encoded *C. trachomatis* protein HagF functions as a direct antagonist of HrcA.

Specifically, the revised manuscript shows that HagF interacts with HrcA by reciprocal pulldown and BLI (Fig. 3A, B, E; Table S2; Fig. S8), weakens HrcA self-association *in vitro* (Fig. 3E; Table S2), reduces HrcA association with CIRCE DNA *in vitro* (Fig. 3F–H), modulates HrcA-regulated gene expression *in vivo* (Figs. 4 and S9), and promotes infectious progeny yield during heat stress (Figs. 2 and S4). These findings establish HagF not merely as an HrcA-regulated gene product, but as a regulator that feeds back on HrcA activity. We also revised the Discussion to explicitly contrast our study with Kozusnik et al.

To address the reviewer's critique that the HagF-HrcA interaction was supported only by pulldown assays, we have substantially expanded the mechanistic analysis. The revised Fig. 3 now includes an AlphaFold3-predicted 2HagF-2HrcA heterotetramer, comparison of the HrcA-HrcA interface in the predicted HrcA homodimer and HagF-containing heterotetramer models (Fig. 3C, D; Table S1), BLI kinetic analysis (Figs. 3E and S8, Table S2), and a CIRCE DNA-binding assay (Fig. 3F-H). These revised modeling and BLI data are also discussed in the revised Discussion, where we explicitly contrast our study with Kozusnik et al.

Major comment 1

No experimental evidence is provided to define the transcription start site of *ctl0271*, so the actual promoter remains unknown. The proposed HrcA-binding site remains hypothetical and should be substantiated through biochemical and/or genetic approaches, such as footprinting assays.

Response

We have added a new Fig. S1 to clarify the promoter architecture. This figure shows the intergenic region between *ctl0270* and *hagF*, the previously reported TSS of *hagF*, and the predicted -35 and -10 elements. These predicted promoter elements differ from the chlamydial σ^{66} consensus elements by only a single nucleotide each.

In addition, our ChIP-qPCR data show HrcA enrichment at the *hagF* promoter region in *C. trachomatis*. We agree that footprinting would provide higher-resolution mapping, but the combination of a previously mapped TSS, near-consensus -35 and -10 elements, a nearby CIRCE-like element, HrcA ChIP enrichment, HrcA-mediated repression, and heat-induced derepression provides strong support for HrcA regulation of this promoter.

We have revised the manuscript to clarify these points and avoid implying nucleotide-resolution mapping beyond the available data.

Major comment 2

The proposed interaction between ctl0271 and HrcA requires more rigorous validation, such as *in vivo* cross-linking followed by immunoprecipitation or bacterial two-hybrid assays.

Response

To strengthen the evidence for HagF-HrcA interaction and its functional consequences, we have expanded the mechanistic analysis beyond the original pulldown assay. The reciprocal pulldown experiments were performed with purified recombinant HagF and HrcA proteins, included tag-swapped configurations, and used negative controls lacking the His-tagged bait protein. We have also added BLI analysis, which provides orthogonal quantitative support for HagF-HrcA association and shows that HagF weakens HrcA self-association *in vitro* (Fig. 3E; Table S2; Fig. S8). Specifically, NH-HrcA bound NS-HagF with an apparent affinity comparable to NH-HrcA binding to NS-HrcA, whereas a preformed NH-HrcA-NS-HagF complex bound NS-HrcA with reduced apparent affinity.

In parallel, AlphaFold3 modeling predicts a 2HagF-2HrcA complex in which the HrcA-HrcA interface is reduced relative to the predicted HrcA homodimer (Fig. 3C, D; Table S1), providing a structural hypothesis consistent with the BLI data. Finally, the CIRCE DNA-binding assay showed that HagF reduces HrcA association with CIRCE DNA (Fig. 3F-H), supporting a functional consequence of HagF-HrcA interaction.

Together, these purified-protein interaction, BLI, modeling, and DNA-binding data strongly support the conclusion that HagF directly interacts with HrcA, weakens HrcA self-association, and reduces HrcA association with CIRCE DNA. We have revised the mechanistic claims accordingly and no longer state that HrcA dimer disruption is directly proven.

Major comment 3

The study uses 45 °C as a heat shock condition, but this may not be physiologically meaningful. A more appropriate approach would be to use temperatures such as 40.5 °C.

Response

As noted above, 40.5 °C is more appropriate for functional studies of chlamydial growth and infectious progeny yield. The 45 °C treatment was used only as a short-term heat shock induction condition in Fig. 1, whereas the functional *hagF* knockdown assays were performed at 40.5 °C. We also added temperature-titration data showing that *hagF* knockdown reduced infectious progeny yield from 39 to 40.5 °C, with the strongest effect at 40.5 °C; no detectable IFUs were recovered at 41 °C from any strain, with or without ATC treatment (Fig. S4).

Major comment 4

The conclusion that HagF overexpression enhances HrcA-regulated chaperone genes and disrupts growth/EB formation would be strengthened by controls such as mutants lacking *hrcA* or other HrcA-regulated genes.

Response

Additional perturbation of the HrcA pathway would be informative, but these experiments would be difficult to interpret because HrcA and its target genes play a central role in bacterial proteostasis—loss of HrcA would lead to chaperone overexpression. Moreover, to the best of our knowledge, stable HrcA-deficient mutants have not been reported, suggesting that loss of HrcA is likely lethal.
