# Supplementary material for: A heat-induced HagF-HrcA regulatory loop promotes chlamydial fitness and infectious progeny yield under stress
Source: PLoS Pathog. 2026 Nov 4;22(11):e1014749. doi: 10.1371/journal.ppat.1014749 (PMC13750654; doi:10.1371/journal.ppat.1014749)
Supplement: Supplementary file 12 [file ppat.1014749.s017.pdf]

Response to Reviewers

We thank the editors and reviewers for their careful evaluation of our revised manuscript. We are pleased that Reviewers 1 and 2 considered their previous concerns adequately addressed. Reviewer 3 raised several additional points for consideration. We have carefully considered each of these comments and revised the manuscript to clarify the interpretation and scope of our conclusions where appropriate. A point-by-point response follows.

Reviewer 3

Major Concerns

1. Validation of the transcription start site: The transcription start site used in this study appears to be based solely on a previous high-throughput TSS-seq analysis. The reviewer suggests independent targeted experimental validation of the proposed TSS.

Response:
We respectfully disagree that independent re-determination of the *hagF* transcription start site is necessary to support the conclusions of the present study. The TSS used in our analysis was experimentally mapped in a previously published deep-sequencing-based transcriptome study of *C. trachomatis* and therefore represents prior experimental evidence rather than a computational prediction.

More importantly, our conclusion that *hagF* is regulated by HrcA does not depend solely on the TSS assignment. It is supported independently by several experimental observations: the presence of a CIRCE-like element upstream of *hagF*, direct enrichment of HrcA at the *hagF* promoter by ChIP-qPCR, reduced *hagF* expression following HrcA overexpression, and strong induction of *hagF* during heat shock. The ChIP-qPCR experiment showed a 19.1-fold enrichment of HrcA at the *ctl0271/hagF* promoter.

We have nevertheless revised the manuscript to make the experimental basis of the TSS assignment explicit. The Results now state that the predicted promoter is positioned relative to "a transcription start site previously mapped by deep-sequencing-based transcriptome analysis," and the Fig. S1 legend now states that "the transcriptional start site of *hagF* was experimentally mapped by deep-sequencing-based transcriptome analysis."

We therefore believe that targeted re-mapping of an experimentally established TSS would provide additional confirmation but is not necessary to support the conclusions of this study.

2. Appropriate controls for the interaction assays: The reviewer suggests an unrelated His-tagged protein control for the pulldown experiments and additional controls for the CIRCE-binding assay, including DNA lacking the CIRCE sequence, an unrelated protein, and heat-treated HrcA.

Response: We agree that the suggested experiments could provide additional validation. However, we do not believe they are required to establish the conclusions currently reached, because the HagF-HrcA interaction is supported by multiple complementary experimental approaches rather than by the pulldown assay alone.

The reciprocal pulldown experiments were performed using purified recombinant proteins in tag-swapped configurations: His-tagged HagF recovered Strep-tagged HrcA, and His-tagged HrcA recovered Strep-tagged HagF. Negative-control reactions omitted the His-tagged bait protein. More

importantly, the interaction is independently supported by BLI, which directly demonstrated HrcA-HagF association and further showed that HagF weakens HrcA self-association. A preformed HrcA-HagF complex retained the ability to bind additional HrcA, but with reduced apparent affinity, including a reduced apparent association rate and increased apparent dissociation rate.

The purpose of the CIRCE-binding experiment was not to re-establish the previously characterized specificity of the HrcA-CIRCE interaction, but to determine whether HagF affects HrcA association with its target DNA. Under these conditions, increasing HagF produced a concentration-dependent reduction in CIRCE-bound HrcA, and this effect was reproduced in four independent experiments.

Likewise, testing heat-treated HrcA would address a different mechanistic question—whether HagF preferentially interacts with a heat-altered HrcA species. As clarified in response to Comment 6 below, our model does not require such preferential binding.

We therefore consider the suggested controls useful potential extensions of the biochemical analysis, but the reciprocal pulldown, orthogonal BLI analysis, and functional CIRCE-binding assay together provide sufficient support for the conclusions that HagF interacts directly with HrcA, weakens HrcA self-association, and reduces HrcA association with CIRCE DNA.

3. Interpretation of AlphaFold3 structural predictions: The reviewer notes that AlphaFold3 predictions are hypothesis-generating and do not by themselves demonstrate an authentic protein complex, and suggests mutational validation of predicted interface residues.

Response:
We agree with the reviewer that AlphaFold3 modeling should be interpreted as hypothesis-generating rather than as an experimental demonstration of a specific complex architecture or interface.

We have revised the Results to make this distinction explicit. The manuscript now states:

"These modeling results generate the hypothesis that HagF binding may weaken, rather than completely disrupt, HrcA self-association, but do not by themselves establish the stoichiometry or molecular interface of an authentic complex."

We also revised the Discussion to state explicitly that "the predicted 2HagF–2HrcA architecture and specific interface residues remain to be experimentally established."

Importantly, the inference that HagF weakens HrcA self-association does not depend on AlphaFold3 alone. It is tested independently by BLI, which showed reduced apparent HrcA-HrcA affinity when HrcA was pre-associated with HagF. Thus, we use the AlphaFold3 model to provide a structural hypothesis consistent with the biochemical data rather than as independent proof of the interaction or its precise architecture.

Targeted mutational analysis of predicted interface residues would be valuable for defining the detailed structural basis of the interaction, but this represents a further mechanistic extension rather than an experiment required for the conclusions presently made.

4. Physiological relevance and cellular consequences of the 45°C heat-shock treatment: The reviewer asks whether 45°C for 30 min causes substantial stress or loss of viability in the host cells or intracellular *Chlamydia* and suggests assessing host-cell viability and, where feasible, chlamydial viability or developmental integrity.

Response:

We appreciate the reviewer's concern and agree that exposure to 45°C imposes heat stress on both the host cell and intracellular *Chlamydia*. We have revised the manuscript to clarify the purpose and scope of this experiment.

The 45°C treatment was used only as a short-term experimental heat-shock induction condition to measure acute transcriptional responses; it was not intended to model physiological fever or sustained chlamydial growth at 45°C. The revised Results now state this explicitly:

"We used 45°C as a short-term experimental heat-shock induction condition, rather than as a model of physiological fever, to robustly activate the chlamydial heat-shock response through heat-induced relief of HrcA-mediated repression."

The manuscript also notes that this acute condition has previously been used to examine HrcA-dependent heat-shock transcription in infected cells.

The relevant question for Fig. 1F is therefore whether short-term heat exposure elicits an interpretable chlamydial heat-shock transcriptional response, rather than whether host-cell physiology remains completely unaltered. Under this condition, *hagF* and the established HrcA-regulated genes *hrcA, grpE, dnaK, groES,* and *groEL* all showed the expected coordinated heat-induced transcriptional response.

Importantly, none of the functional conclusions concerning HagF depends on growth at 45°C. The functional *hagF* knockdown experiments were performed over the substantially lower 39–40.5°C range. *hagF* knockdown reduced infectious progeny yield throughout this range, with the strongest effect at 40.5°C, whereas 41°C completely prevented recovery of detectable inclusion-forming units (IFUs) from all strains.

Previous work has also demonstrated that the effects of heat shock on *C. trachomatis* can be reversible after limited exposure, and this literature is already cited in the manuscript.

We therefore believe that detailed characterization of host-cell viability and heat-stress physiology at 45°C would address an additional biological question concerning the host response to severe thermal stress, rather than being necessary to interpret the acute transcriptional induction experiment presented here.

5. Proposed essentiality of HrcA: The reviewer notes that the absence of reported stable *hrcA*-deficient mutants does not establish that *hrcA* is essential and suggests qualification unless direct experimental evidence is available.

Response:

We agree that the absence of reported viable *hrcA*-deficient mutants does not establish that *hrcA* is essential. Neither the previous nor the current version of the manuscript makes a claim regarding *hrcA* essentiality. In our previous response, the point was raised only in discussing why additional perturbation of the HrcA pathway could be difficult to interpret.

Experimentally establishing the essentiality or non-essentiality of *hrcA* in *C. trachomatis* would require a dedicated genetic analysis and is outside the scope of the present study. Importantly, no conclusion regarding HagF-HrcA regulation depends on HrcA essentiality.

6. Mechanistic model for the role of HagF: The reviewer asks whether HagF is proposed to preferentially interact with heat-destabilized HrcA and, if heat-induced destabilization of HrcA already promotes dissociation from CIRCE, what additional regulatory function HagF provides.

Response:
We appreciate this comment because it identified a point in the model that required clearer explanation. We do not propose that HagF preferentially recognizes or binds a heat-destabilized form of HrcA.

Our model is instead sequential and additive. Heat-induced destabilization of HrcA provides initial relief from repression, allowing induction of hagF along with other HrcA-regulated genes. As HagF accumulates, it binds residual functional HrcA and further attenuates HrcA-mediated repression. Thus, HagF provides a second regulatory layer rather than replacing the primary heat-dependent effect on HrcA.

The functional evidence for this additional layer is provided by the *hagF* knockdown experiments. At 40.5°C, when heat stress itself is already relieving HrcA repression, *hagF* silencing nevertheless significantly reduced expression of *hrcA*, *grpE*, and *dnaK*. This shows that heat-induced reduction of HrcA activity does not eliminate the regulatory contribution of HagF.

We revised the Discussion accordingly:

"Under heat stress, HrcA destabilization provides the initial relief of repression and permits induction of *hagF*. Accumulated HagF then binds residual HrcA and further attenuates its repressor activity, thereby amplifying chaperone gene expression... This model does not require preferential binding of HagF to a heat-destabilized HrcA species; rather, HagF provides an additional layer of derepression by antagonizing residual functional HrcA during heat stress."

This clarification also explains the promoter-specific phenotype observed during *hagF* knockdown. The *hrcA-grpE-dnaK* operon remains more dependent on HagF-mediated relief of HrcA repression, whereas the *groES-groEL* promoter may be sufficiently derepressed by heat-induced HrcA destabilization alone.

We believe the revised text now clearly distinguishes the primary heat-induced effect on HrcA from the subsequent HagF-dependent amplification of derepression.

Minor Issue: The manuscript should clarify how *hagF* was initially identified as a new HrcA-regulated gene and distinguish the present findings from the report of Kozusnik et al.

Response:
The manuscript already identified our previous heat shock transcriptomic study (Huang et al., 2021; doi:10.3389/fmicb.2021.812448) as the origin of the present investigation of *hagF*. In that study, *ctl0271* was identified as a gene strongly induced by heat shock. In the present study, the presence of a CIRCE-like element upstream of *ctl0271* led us to test directly whether it is regulated by HrcA.

We have revised the Discussion to distinguish clearly between these prior observations and the experimental and mechanistic evidence provided here. The revised Discussion now states:

"While finalizing this study, Kozusnik et al. reported stress-induced expression of *ctl0271* (*hagF*) and its orthologs in Chlamydia-like organisms and proposed that these genes are HrcA targets. In contrast to that proposed assignment, our study directly demonstrates HrcA binding to the *ctl0271* promoter and HrcA-dependent repression in *C. trachomatis* and further shows that the encoded protein HagF is a direct antagonist of HrcA..."
