# Supplementary material for: A heat-induced HagF-HrcA regulatory loop promotes chlamydial fitness and infectious progeny yield under stress
Source: PLoS Pathog. 2026 Nov 4;22(11):e1014749. doi: 10.1371/journal.ppat.1014749 (PMC13750654; doi:10.1371/journal.ppat.1014749)
Supplement: Supplementary file 13 [file ppat.1014749.s001.zip › References.docx]

References:

1. Tan M. 2006. Regulation of gene expression, p 103–131. *In* Bavoil P, Wyrick P (ed), *Chlamydia* Genomics and Pathogenesis. Horizon Bioscience, Wymondham, Norfolk, UK.

2. Albrecht M, Sharma CM, Reinhardt R, Vogel J, Rudel T. 2010. Deep sequencing-based discovery of the *Chlamydia trachomatis* transcriptome. Nucleic acids research 38:868–877.
