# Supplementary material for: A heat-induced HagF-HrcA regulatory loop promotes chlamydial fitness and infectious progeny yield under stress
Source: PLoS Pathog. 2026 Nov 4;22(11):e1014749. doi: 10.1371/journal.ppat.1014749 (PMC13750654; doi:10.1371/journal.ppat.1014749)
Supplement: Supplementary file 13 [file ppat.1014749.s001.zip › S1_Fig.pdf]

A

Consensus σ^{66} core promoter

TTGACAnnnnnnnnnnnnnnnnnTATAAT

B

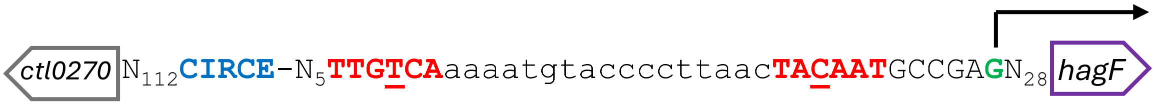

Fig. S1. Predicted σ^{66} promoter and CIRCE element upstream of hagF (ctl0271). (A) Consensus sequence of the chlamydial σ^{66} core promoter (1). (B) Sequence analysis of the intergenic region between *ctl0270* and *hagF* identified a putative CIRCE element (Fig. 1) and a predicted σ^{66}-dependent promoter. The previously reported transcriptional start site of *hagF* is indicated in green (2). The predicted -35 (TGTACA) and -10 (TACAAT) elements, shown in red, each differ from the corresponding consensus elements of σ^{66} by a single nucleotide, with the unmatched nucleotide underlined.

References:

1. Tan M. 2006. Regulation of gene expression, p 103–131. *In* Bavoil P, Wyrick P (ed), *Chlamydia* Genomics and Pathogenesis. Horizon Bioscience, Wymondham, Norfolk, UK.
2. Albrecht M, Sharma CM, Reinhardt R, Vogel J, Rudel T. 2010. Deep sequencing-based discovery of the *Chlamydia trachomatis* transcriptome. Nucleic acids research 38:868–877.
