# Supplementary material for: A heat-induced HagF-HrcA regulatory loop promotes chlamydial fitness and infectious progeny yield under stress
Source: PLoS Pathog. 2026 Nov 4;22(11):e1014749. doi: 10.1371/journal.ppat.1014749 (PMC13750654; doi:10.1371/journal.ppat.1014749)
Supplement: Supplementary file 14 [file ppat.1014749.s009.zip › Reference.docx]

References:

1. Wurihan W, Zou Y, Weber AM, Weldon K, Huang Y, Bao X, et al. Identification of a GrgA-Euo-HrcA transcriptional regulatory network in *Chlamydia*. mSystems. 2021;6(4):e0073821. Epub 2021/08/04. doi: 10.1128/mSystems.00738-21. PubMed PMID: 34342542; PubMed Central PMCID: PMCPMC8409740.

2. Wurihan W, Weber AM, Gong Z, Lou Z, Sun S, Zhou J, et al. GrgA overexpression inhibits *Chlamydia trachomatis* growth through sigma66- and sigma28-dependent mechanisms. Microb Pathog. 2021;156:104917. doi: doi.org/10.1016/j.micpath.2021.104917. PubMed Central PMCID: PMCPMCID: PMC8187326.
