# Supplementary material for: A heat-induced HagF-HrcA regulatory loop promotes chlamydial fitness and infectious progeny yield under stress
Source: PLoS Pathog. 2026 Nov 4;22(11):e1014749. doi: 10.1371/journal.ppat.1014749 (PMC13750654; doi:10.1371/journal.ppat.1014749)
Supplement: Peer review history

# Peer review: Decision Letter 0

**DOI:** 10.1371/journal.ppat.1014749.r001

**Authors:** Thomas Guillard, Leanid Laganenka

14 May 2026

PPATHOGENS-D-26-00858

A heat-induced HagF–HrcA regulatory loop promotes chlamydial fitness and infectious progeny formation under stress

PLOS Pathogens

Dear Dr. Fan,

Thank you for submitting your manuscript to PLOS Pathogens. After careful consideration, we feel that it has merit but does not fully meet PLOS Pathogens's publication criteria as it currently stands. Therefore, we invite you to submit a revised version of the manuscript that addresses the points raised during the review process.

Please submit your revised manuscript by Jul 13 2026 11:59PM. If you will need more time than this to complete your revisions, please reply to this message or contact the journal office at plospathogens@plos.org. When you're ready to submit your revision, log on to https://www.editorialmanager.com/ppathogens/ and select the 'Submissions Needing Revision' folder to locate your manuscript file.

Please include the following items when submitting your revised manuscript:

* A letter that responds to each point raised by the editor and reviewer(s). You should upload this letter as a separate file labeled 'Response to Reviewers'. This file does not need to include responses to any formatting updates and technical items listed in the 'Journal Requirements' section below.

* A marked-up copy of your manuscript that highlights changes made to the original version. You should upload this as a separate file labeled 'Revised Manuscript with Track Changes'.

* An unmarked version of your revised paper without tracked changes. You should upload this as a separate file labeled 'Manuscript'.

If you would like to make changes to your financial disclosure, competing interests statement, or data availability statement, please make these updates within the submission form at the time of resubmission. Guidelines for resubmitting your figure files are available below the reviewer comments at the end of this letter. As the corresponding author, your ORCID iD is verified in the submission system and will appear in the published article. PLOS supports the use of ORCID, and we encourage all coauthors to register for an ORCID iD and use it as well. Please encourage your coauthors to verify their ORCID iD within the submission system before final acceptance, as unverified ORCID iDs will not appear in the published article. Only the individual author can complete the verification step; PLOS staff cannot verify ORCID iDs on behalf of authors.

We look forward to receiving your revised manuscript.

Kind regards,

Leanid Laganenka

Guest Editor

PLOS Pathogens

Thomas Guillard

Section Editor

PLOS Pathogens Sumita Bhaduri-McIntosh

Editor-in-Chief

PLOS Pathogens

orcid.org/0000-0003-2946-9497

Michael Malim

Editor-in-Chief

PLOS Pathogens

orcid.org/0000-0002-7699-2064

**Additional Editor Comments :**

All reviewers agree that your study addresses an interesting and potentially important aspect of the heat-shock response in C. trachomatis. The reviewers also recognize the strengths of the molecular and genetic approaches used in the study. However, they raise several significant concerns regarding the strength of the mechanistic conclusions and the extent to which the current data support the proposed model.

In preparing your revision, please pay particular attention to the following issues. First, additional experimental support is needed for the proposed HagF-HrcA interaction and its mechanistic consequences. While the current data are suggestive, the reviewers felt that the evidence is not yet sufficient to support some of the mechanistic claims presented in the manuscript.

Second, the reviewers noted that the causal relationship between HagF function and EB formation remains insufficiently demonstrated. Please provide additional experimental support for this conclusion or revise the interpretation accordingly. I would also suggest to reconsider the instances where you use the term “EB formation”, as the current data more directly support effects on “EB viability” rather than conclusively demonstrating a halt in developmental progression. To demonstrate impaired EB formation, you might want to show not only reduced EB viability but also that fewer EBs are actually generated. This could be addressed, for example, by comparing expression of EB-specific genes late in the chlamydial developmental cycle or by immunofluorescence labeling using EB-specific markers such as OmcB.

Third, the interpretation of the gene expression changes observed upon HagF overexpression requires additional controls. In particular, please address whether these effects could arise from nonspecific consequences of protein overexpression (for example, through inclusion of an inactive HagF variant control), or otherwise adjust the conclusions drawn from these experiments.

The reviewers also raised concerns regarding the assignment of the putative -35 and CIRCE elements in the ctl0271 promoter region. Please provide additional experimental evidence or clarification supporting these assignments.

In addition, please justify the physiological relevance of the heat shock conditions used in the study (45 °C versus 40.5 °C). Finally, in light of the concerns raised regarding the novelty of the work, I encourage you to more clearly define how the present study advances beyond prior reports and to more explicitly highlight the novel contributions of the manuscript.

**Journal Requirements:**

1) Please ensure that the CRediT author contributions listed for every co-author are completed accurately and in full.At this stage, the following Authors/Authors require contributions: Guangming Zhong, and Huizhou Fan. Please ensure that the full contributions of each author are acknowledged in the "Add/Edit/Remove Authors" section of our submission form.The list of CRediT author contributions may be found here: https://journals.plos.org/plospathogens/s/authorship#loc-author-contributions 2) We ask that a manuscript source file is provided at Revision. Please upload your manuscript file as a .doc, .docx, .rtf or .tex. If you are providing a .tex file, please upload it under the item type u2018LaTeX Source Fileu2019 and leave your .pdf version as the item type u2018Manuscriptu2019. 3) We have noticed that you have uploaded Supporting Information files, but you have not included a list of legends. Please add a full list of legends for your Supporting Information files after the references list. 4)

Some material included in your submission may be copyrighted. According to PLOS’s copyright policy, authors who use figures or other material (e.g., graphics, clipart, maps) from another author or copyright holder must demonstrate or obtain permission to publish this material under the Creative Commons Attribution 4.0 International (CC BY 4.0) License used by PLOS journals. Please closely review the details of PLOS’s copyright requirements here: PLOS Licenses and Copyright. If you need to request permissions from a copyright holder, you may use PLOS's Copyright Content Permission form.

Please respond directly to this email and provide any known details concerning your material's license terms and permissions required for reuse, even if you have not yet obtained copyright permissions or are unsure of your material's copyright compatibility. Once you have responded and addressed all other outstanding technical requirements, you may resubmit your manuscript within Editorial Manager.

Potential Copyright Issues:

We note that Figures 1, 6, and S6 are created through BioRender. Please confirm that you hold a Premium account and provide a pdf copy of the CC BY 4.0 Licence as provided by BioRender. For instructions on how to generate a CC BY 4.0 license for your figure, please see the guidelines here: https://help.biorender.com/hc/en-gb/articles/21282341238045-Publishing-in-open-access-resources

Please ensure the figure legend contains appropriate attribution text & a link to biorender.com

If you are using the free assets from BioRender, we are unable to publish these images as they are licenced under a stricter licence than CC BY 4.0. In this case we ask you to remove the BioRender images and replace them with open source alternatives.

See these open source resources you may use to replace images / clip-art:

- https://bioart.niaid.nih.gov/

- https://bioicons.com/

- https://healthicons.org/

- https://scidraw.io/

- https://reactome.org/icon-lib

- https://www.phylopic.org/images

- https://journals.plos.org/plosbiology/article?id=10.1371/journal.pbio.3002395.

5) Please provide a complete Data Availability Statement in the submission form, ensuring you include all necessary access information or a reason for why you are unable to make your data freely accessible. If your research concerns only data provided within your submission, please write "All data are in the manuscript and/or supporting information files" as your Data Availability Statement. Note: Authors must share the “minimal data set” for their submission. PLOS defines the minimal data set to consist of the data required to replicate all study findings reported in the article, as well as related metadata and methods (https://journals.plos.org/plosone/s/data-availability#loc-minimal-data-set-definition).For example, authors should submit the following data: 1) The values behind the means, standard deviations and other measures reported;2) The values used to build graphs;3) The points extracted from images for analysis..Authors do not need to submit their entire data set if only a portion of the data was used in the reported study.If your submission does not contain these data, please either upload them as Supporting Information files or deposit them to a stable, public repository and provide us with the relevant URLs, DOIs, or accession numbers. For a list of recommended repositories, please see https://journals.plos.org/plosone/s/recommended-repositories. If there are ethical or legal restrictions on sharing a de-identified data set, please explain them in detail (e.g., data contain potentially sensitive information, data are owned by a third-party organization, etc.) and who has imposed them (e.g., an ethics committee). Please also provide contact information for a data access committee, ethics committee, or other institutional body to which data requests may be sent. If data are owned by a third party, please indicate how others may request data access. 6) Please amend your detailed Financial Disclosure statement. This is published with the article. It must therefore be completed in full sentences and contain the exact wording you wish to be published.1) State what role the funders took in the study. If the funders had no role in your study, please state: "The funders had no role in study design, data collection and analysis, decision to publish, or preparation of the manuscript.". Note: If the reviewer comments include a recommendation to cite specific previously published works, please review and evaluate these publications to determine whether they are relevant and should be cited. There is no requirement to cite these works unless the editor has indicated otherwise.

**Reviewers' Comments:**

Reviewer's Responses to Questions

**Part I - Summary**

Please use this section to discuss strengths/weaknesses of study, novelty/significance, general execution and scholarship.

Reviewer #1: This is an interesting study that examines the heat response in C. trachomatis, and identifies a new gene that is regulated by the heat shock regulator HrcA. They name this new gene hagF and demonstrate that it is induced by heat shock and/or hrcA expression, that its reduction leads to a decrease in EB formation, that its protein binds HrcA/blocks DNA binding, and that this leads to decrease in its activity. It is generally well written and presented although there are some limitations that affect the reliability of the interpretations as written.

Reviewer #2: In this manuscript, Huang and colleagues characterize a new component (HagF) of the heat-stress regulatory pathway in the intracellular pathogen Chlamydia trachomatis. The authors use in silico modeling, molecular, genetic, and immunofluorescence techniques (all strengths) to investigate the contributions of HagF to the chlamydial stress response pathway, and convincingly demonstrate its importance during heat shock in this pathogen. While the study is well-designed and the approaches rigorous (the section demonstrating HagF interacts with HrcA and interferes with its ability to bind CIRCE DNA was particularly convincing), some of the authors' conclusions drawn appear to exceed that of their reported results. All of these instances can be easily addressed with either the inclusion of additional data or a reconsideration of the language used in describing their model. With the incorporation of changes suggested below, the overall significance of their findings appear to be high, and broaden the elements of this highly conserved stress response pathway.

Reviewer #3: The study has some merit in that it examines heat-shock-responsive regulation in obligate intracellular bacteria within the Chlamydiales, an area of continuing interest. However, its novelty appears limited. Much of the work parallels prior findings reported by Kozusnik et al. in Microbiology (Reading) (2025), including the identification of ctl0271 in C. trachomatis as a homolog of wcw_0502 in a distant chlamydial species Waddlia chondrophila, both of which are induced under heat-shock conditions in an HrcA-dependent manner. Additionally, the presence of a putative CIRCE consensus sequence recognized by HrcA had already been described in that earlier work. The only potentially interesting hypothesis concerns a possible interaction between ctl0271 and HrcA; however, this is based solely on an in vitro pull-down assay using purified HrcA and ctl0271 proteins, without the inclusion of appropriate negative controls.

In terms of execution, while the study reproduces these observations, it does not substantially extend them. In particular, it lacks deeper mechanistic investigation into the HrcA–ctl0271 (also referred to as HagF) regulatory pathway. This limits the overall significance and impact of the work, as it does not sufficiently advance understanding beyond what has already been established.

From a scholarly perspective, the manuscript would benefit from clearer differentiation from prior studies and a stronger emphasis on novel insights. Without additional mechanistic data or conceptual advances, the contribution remains incremental rather than transformative.

**********

**Part II – Major Issues: Key Experiments Required for Acceptance**

Please use this section to detail the key new experiments or modifications of existing experiments that should be absolutely required to validate study conclusions.

Generally, there should be no more than 3 such required experiments or major modifications for a "Major Revision" recommendation. If more than 3 experiments are necessary to validate the study conclusions, then you are encouraged to recommend "Reject".

Reviewer #1: Major comments:

1. The causality between HagF-HcrA regulation and EB formation is not proven. A phenotype is shown whereby hagF k/d reduces infectious EB at 40.5C, but this does not directly show it is caused by the HrcA regulon rather than another function. Additional experiments should be carried out to demonstrate this, or the stated conclusions should be weakened.

2. The mechanistic claim about HagF disrupting HrcA dimerization is not directly proven. There is a reciprocal pulldown, and alphafold modelling, but no direct evidence about HrcA dimerization. This could be done by SEC-MALs, ultracentrifugation etc, and also point mutagenesis to test predicted interface.

3. ChIP data uses tagged and induced HrcA. Native promoter occupancy is not shown.

Reviewer #2: 1- Figure 4:

- Given the authors’ hypothesis, why do groES and groEL levels increase upon hagF over-expression but do not decrease upon hagF silencing? (the authors mention this but do not address it in lines 386-388)

- Playing Devils Advocate, the enhanced expression of groES and groEL may simply be the result of over-expressing any protein from an inducible promoter via plasmid in Chlamydia trachomatis. Per the authors' model (Fig. 6) this would result in GroESL binding unfolded / over-expressed protein, thereby indirectly influencing expression of hrcA, grpE, dnaK, etc …

- Taken a step farther, this further impacts the interpretation of Fig 4A. The over-expression of any protein may trigger GroESL and relieve repression of the hrcA operon. If they wish to include this data in the manuscript, the authors likely need to demonstrate that over-expression of an inactive form of HagF has no impact on the expression of hrcA, grpE, dnaK, groES, and groEL.

2- Figure 2:

- Given that IFU assays are actually ‘EB viability assays’, how do the authors distinguish whether differentiation is being halted or EB viability is simply being reduced under hagF depletion? A demonstration of transcriptional differences between control and hagF knock downs for genes required for EB formation would allow for a more convincing argument to be made. Alternatively, this could also be achievable by immunofluorescence labeling with EB-specific markers, such as anti-OmcB, late in the chlamydial developmental cycle.

Reviewer #3: Overall, the experimental design appears preliminary and raises several major concerns:

1. The description in lines 99-102 states that “the ctl0271 CIRCE element is located five nucleotides upstream of the -35 core promoter element, the same positioning as the CIRCE elements found in the promoters of the hrcA-grpE–dnaK and groES–groEL operons.” However, no experimental evidence is provided to define the transcription start site of the ctl0271 gene (thus the actual ctl0271 promoter remains unknown). Without clearly establishing the promoter region, it is unclear how the authors determined the location of the -35 element or validated the CIRCE element shown in Figure 1C. Moreover, the proposed HrcA-binding site remains hypothetical and should be substantiated through biochemical and/or genetic approaches, such as footprinting assays.

2. The proposed interaction between ctl0271 and HrcA requires more rigorous validation. More definitive genetic and biochemical methods with appropriate negative controls should be employed, such as in vivo cross-linking followed by immunoprecipitation or bacterial two-hybrid assays.

3. There are concerns regarding the physiological relevance of the experimental conditions used to test the hypothesis. For instance, the study uses 45 °C as a heat shock condition (Figure 1), yet it is unclear whether Chlamydia, as a human obligate intracellular pathogen, would encounter such temperatures in vivo. This raises questions about whether 45 °C represents a physiologically meaningful condition. A more appropriate and consistent approach would be to use temperatures such as 40.5 °C, which were applied in other experiments (e.g., Figure 2).

4. Based on Figure 5, the authors suggest that overexpression of HagF enhances the expression of HrcA-regulated chaperone genes and disrupts chlamydial growth and elementary body (EB) formation under non-stressed conditions. This conclusion would be strengthened by including additional controls, such as parallel growth analyses of mutants lacking hrcA or other HrcA-regulated genes, to confirm that the observed effects are specifically mediated through this regulatory pathway.

**********

**Part III – Minor Issues: Editorial and Data Presentation Modifications**

Please use this section for editorial suggestions as well as relatively minor modifications of existing data that would enhance clarity.

Reviewer #1: Minor comments

1. The title/discussion imply a broad “stress” phenotype but the actual data only looks at heat shock. Other stressors are not explored. The title/discussed should be adjusted to reflect this.

2. There is no rescue experiment for the hagF knockdown experiments in Fig 2. This would strengthen this experiment.

3. Could elaborate in the introduction about the physiological role of heat stress in Chlamydiae, presumably during fever

4. Fig 1, figure legend, should detail meaning of N9 and N5

5. Elaborate on whether there are other genes in the genome with predicted CIRCE elements, or are all listed in Fig. 1.

6. Fig 2 – A contains two panels. This is confusing and would be better as two separate panels, one for each graph.

Reviewer #2: Lines 60-62: Please provide references for this statement.

Line 62: Generally, chlamydial persistence has been understood to act as a means of halting chlamydial development until an extracellular / intracellular stressor has passed, and only then does development continue. The concept of sustaining EB formation during an ongoing stressor is not entirely intuitive. The idea being that it would be disadvantageous for Chlamydia to continue its developmental cycle under conditions that would prove deleterious to its extracellular form. The authors should try to provide a better rationale for why this may differ for heat stress, when compared to several other stressors (such as high levels of interferon gamma, antibiotics, nutrient starvation, etc).

Figure 1:

- Image resolution appears below 300dpi.

- While a temperature of 45 C is convenient for demonstrating this stress response pathway in vitro (prior reports have used 42C in the characterization of htrA) this temperature range is seldom experienced in human beings. For context, a high fever is ~40C with only a few reported cases in the mid-40s in heat stroke patients. Do the authors suspect that this stress pathway is an evolutionary holdover from a time when Chlamydiae resided within host organisms that experienced greater temperature fluctuations? Alternatively, while responsive to heat stress, is there a more physiologically relevant stressor that Chlamydia may have repurposed this pathway for?

Figure 2:

- Why is 40.5 C used here while 45 C was used in Figure 1? (the authors need to justify this discrepancy in the text)

- Why were EB yields measured at different time points for different temperatures?

- In order to eliminate confounding variables, it would help to demonstrate that cellular autophagy levels in infected cells grown at 40.5 C are similar across all three chlamydia strains under aTc induction (autophagy is generally enhanced at heightened temperatures).

Lines 202-204: The results suggest fewer viable EBs when dagF is silenced. To demonstrate a reduction in EB formation, the authors would need to show either i) fewer EBs present in their dagF-deplete strain compared to their control and/or ii) delayed / altered expression of EB-formation related genes.

Lines 210-213: While colocalization is evident in these images and HIS signal appears to be specific to bacteria, the resolution provided does not eliminate the possibility that HagF could also be associated with the bacterial surface. A surface marker and a high-resolution image would be needed to effectively rule out this possibility.

Figure 5:

- Why is there a reduction in genome counts in addition to a reduction in viable EB counts when you over-express hagF at 37C?

- Does this trend hold when comparing growth under native/inducing conditions at 40.5 C?

Lines 388-390: The authors readout for these assays are viable EBs, not "EB formation" (what if EBs are forming and simply become non-viable as a result of heat stress in the absence of HagF?)

Line 398-400: Again, this is not entirely persuasive. Febrile hosts are unlikely to be successful in spreading urogenital infections between individuals (for a variety of reasons). It is not entirely clear the degree to which an inflamed environment would even be beneficial for chlamydia cell-to-cell spread. Increases in temperature can act on immune cells, both enhancing their activity and their production of interferon gamma. If the authors wish to make this argument both here and throughout the text, they need to make it while incorporating a stronger pathogenic / immunological context. Alternatively, they should consider the possibility that this pathway has been repurposed by these microbes to be utilized more broadly during a variety of stress responses.

Reviewer #3: (No Response)

**********

PLOS authors have the option to publish the peer review history of their article (what does this mean?). If published, this will include your full peer review and any attached files.

If you choose “no”, your identity will remain anonymous but your review may still be made public.

**Do you want your identity to be public for this peer review?**  For information about this choice, including consent withdrawal, please see our Privacy Policy.

Reviewer #1: No

Reviewer #2: No

Reviewer #3: No

[NOTE: If reviewer comments were submitted as an attachment file, they will be attached to this email and accessible via the submission site. Please log into your account, locate the manuscript record, and check for the action link "View Attachments". If this link does not appear, there are no attachment files.]

**Figure resubmission:**

While revising your submission, we strongly recommend that you use PLOS’s NAAS tool (https://ngplosjournals.pagemajik.ai/artanalysis) to test your figure files. NAAS can convert your figure files to the TIFF file type and meet basic requirements (such as print size, resolution), or provide you with a report on issues that do not meet our requirements and that NAAS cannot fix.

After uploading your figures to PLOS’s NAAS tool - https://ngplosjournals.pagemajik.ai/artanalysis, NAAS will process the files provided and display the results in the "Uploaded Files" section of the page as the processing is complete. If the uploaded figures meet our requirements (or NAAS is able to fix the files to meet our requirements), the figure will be marked as "fixed" above. If NAAS is unable to fix the files, a red "failed" label will appear above. When NAAS has confirmed that the figure files meet our requirements, please download the file via the download option, and include these NAAS processed figure files when submitting your revised manuscript.

**Reproducibility:**

To enhance the reproducibility of your results, we recommend that authors of applicable studies deposit laboratory protocols in protocols.io, where a protocol can be assigned its own identifier (DOI) such that it can be cited independently in the future. Additionally, PLOS ONE offers an option to publish peer-reviewed clinical study protocols. Read more information on sharing protocols at https://plos.org/protocols?utm_medium=editorial-email&utm_source=authorletters&utm_campaign=protocols

# Author comment: Author response to Decision Letter 1

**DOI:** 10.1371/journal.ppat.1014749.r002

19 Aug 2026

Attachment Submitted filename: Rebuttal.pdf

# Peer review: Decision Letter 1

**DOI:** 10.1371/journal.ppat.1014749.r003

**Authors:** Thomas Guillard, Leanid Laganenka

23 Sep 2026

PPATHOGENS-D-26-00858R1

A heat-induced HagF-HrcA regulatory loop promotes chlamydial fitness and infectious progeny yield under stress

PLOS Pathogens

Dear Dr. Fan,

Thank you for submitting the revised version of your manuscript to PLOS Pathogens. Although two reviewers agreed that their comments were adressed, one reviewer still raised several points that you might want to consider. Therefore, we invite you to submit a revised version of the manuscript that addresses the points raised during the review process.

Please submit your revised manuscript by Nov 22 2026 11:59PM. If you will need more time than this to complete your revisions, please reply to this message or contact the journal office at plospathogens@plos.org. When you're ready to submit your revision, log on to https://www.editorialmanager.com/ppathogens/ and select the 'Submissions Needing Revision' folder to locate your manuscript file.

Please include the following items when submitting your revised manuscript:

* A letter that responds to each point raised by the editor and reviewer(s). You should upload this letter as a separate file labeled 'Response to Reviewers'. This file does not need to include responses to any formatting updates and technical items listed in the 'Journal Requirements' section below.

* A marked-up copy of your manuscript that highlights changes made to the original version. You should upload this as a separate file labeled 'Revised Manuscript with Track Changes'.

* An unmarked version of your revised paper without tracked changes. You should upload this as a separate file labeled 'Manuscript'.

If you would like to make changes to your financial disclosure, competing interests statement, or data availability statement, please make these updates within the submission form at the time of resubmission. Guidelines for resubmitting your figure files are available below the reviewer comments at the end of this letter. As the corresponding author, your ORCID iD is verified in the submission system and will appear in the published article. PLOS supports the use of ORCID, and we encourage all coauthors to register for an ORCID iD and use it as well. Please encourage your coauthors to verify their ORCID iD within the submission system before final acceptance, as unverified ORCID iDs will not appear in the published article. Only the individual author can complete the verification step; PLOS staff cannot verify ORCID iDs on behalf of authors.

We look forward to receiving your revised manuscript.

Kind regards,

Leanid Laganenka

Guest Editor

PLOS Pathogens

Thomas Guillard

Section Editor

PLOS PathogensSumita Bhaduri-McIntosh

Editor-in-Chief

PLOS Pathogens

orcid.org/0000-0003-2946-9497

Dominique Soldati-Favre

Editor-in-Chief

PLOS Pathogens

orcid.org/0000-0003-4156-2109

**Journal Requirements:**

1. Please amend your detailed Financial Disclosure statement. This is published with the article. It must therefore be completed in full sentences and contain the exact wording you wish to be published.

a.State what role the funders took in the study. If the funders had no role in your study, please state: “The funders had no role in study design, data collection and analysis, decision to publish, or preparation of the manuscript.”

b. If any authors received a salary from any of your funders, please state which authors and which funders.

2. Your current Financial Disclosure states, 'This work was supported by the National Institutes of Health grant numbers AI140167 and AI154305 (to H.F.) and AI182210 (to G.Z. and H.F.). Y.H. was supported by a scholarship (award no. 201806370171) from the China Scholarship Council (CSC) from September 2018 to September 2020' . However, your funding information on the submission form states the 'Division of Microbiology and Infectious Diseases, National Institute of Allergy and Infectious Diseases- 182210 to Guangming Zhong' .  Please indicate by return email the full and correct funding information for your study and confirm the order in which funding contributions should appear. Please be sure to indicate whether the funders played any role in the study design, data collection and analysis, decision to publish, or preparation of the manuscript.

3. Some material included in your submission may be copyrighted. According to PLOS’s copyright policy, authors who use figures or other material (e.g., graphics, clipart, maps) from another author or copyright holder must demonstrate or obtain permission to publish this material under the Creative Commons Attribution 4.0 International (CC BY 4.0) License used by PLOS journals. Please closely review the details of PLOS’s copyright requirements here: PLOS Licenses and Copyright. If you need to request permissions from a copyright holder, you may use PLOS's Copyright Content Permission form.

Please respond directly to this email and provide any known details concerning your material's license terms and permissions required for reuse, even if you have not yet obtained copyright permissions or are unsure of your material's copyright compatibility. Once you have responded and addressed all other outstanding technical requirements, you may resubmit your manuscript within Editorial Manager.

Potential Copyright Issues:

a. Figure 3F : Please confirm whether you drew the images / clip-art within the figure panels by hand. If you did not draw the images, please provide (a) a link to the source of the images or icons and their license / terms of use; or (b) written permission from the copyright holder to publish the images or icons under our CC BY 4.0 license. Alternatively, you may replace the images with open source alternatives. See these open source resources you may use to replace images / clip-art:

- https://commons.wikimedia.org

- https://openclipart.org/

If the reviewer comments include a recommendation to cite specific previously published works, please review and evaluate these publications to determine whether they are relevant and should be cited. There is no requirement to cite these works unless the editor has indicated otherwise.

[Note: HTML markup is below. Please do not edit.]

**Reviewers' Comments:**

Reviewer's Responses to Questions

**Part I - Summary**

Please use this section to discuss strengths/weaknesses of study, novelty/significance, general execution and scholarship.

Reviewer #1: The authors have adequately addressed my concerns

Reviewer #2: (No Response)

Reviewer #3: Please refer to my comment from the previous round of review.

**********

**Part II – Major Issues: Key Experiments Required for Acceptance**

Please use this section to detail the key new experiments or modifications of existing experiments that should be absolutely required to validate study conclusions.

Generally, there should be no more than 3 such required experiments or major modifications for a "Major Revision" recommendation. If more than 3 experiments are necessary to validate the study conclusions, then you are encouraged to recommend "Reject".

Reviewer #1: (No Response)

Reviewer #2: The authors have adequately addressed all of my previous concerns. They have provided additional clarifying data and/or modified their text to take into account the few limitations of the approaches they have utilized. As a result, their experimental results now more accurately match their conclusions.

Reviewer #3: Overall, several of the major concerns raised during the first round of review remain unresolved or have been only partially addressed. The comments below follow up on these issues and identify points that, in my view, still require clarification or additional experimental support.

Major Concerns

1. Validation of the transcription start site: The transcription start site (TSS) used in this study appears to be based solely on a previous high-throughput TSS-seq analysis from another research group. While TSS-seq is highly valuable and generally appropriate for genome-wide analyses and expression profiling, additional experimental validation is necessary when precise identification of a specific TSS is important for the mechanistic characterization of an individual promoter. Given the importance of the identified TSS to the conclusions of this study, I remain concerned that independent validation of this site has not been provided. The authors should consider experimentally validating the proposed TSS using an appropriate targeted approach.

2. Appropriate controls for the interaction assays: Appropriate negative controls are still missing from several of the experiments. In particular, relying primarily on His-tag pull-down assays to demonstrate a specific protein-protein interaction can be problematic because of potential nonspecific binding and false-positive interactions. Particularly, for the experiments shown in Fig. 3A and 3B, in which His-tagged HrcA or HagF was used to pull down the interacting protein, the authors should consider including an unrelated His-tagged protein as a negative control to assess nonspecific binding. Similarly, the experiments presented in Fig. 3F-3H will definitely benefit from additional controls. For example, the authors should consider including a biotinylated DNA fragment lacking the CIRCE sequence, an unrelated protein, and heat-treated HrcA (e.g., HrcA exposed to 40.5°C) as appropriate controls. These controls should help establish the specificity of the observed protein-DNA and/or protein-protein interactions and strengthen the conclusions drawn from these experiments.

3. Interpretation of AlphaFold3 structural predictions: I remain uncertain about the extent to which the AlphaFold3 predictions presented in the manuscript can be interpreted as evidence for an authentic protein complex. My understanding is that such predictions provide structural models that can generate hypotheses regarding potential protein-protein interactions and identify candidate interface residues, but they do not, by themselves, experimentally demonstrate that the predicted interaction occurs. In this case, specific interactions or interface residues predicted by AlphaFold3 are central to the proposed HrcA-HagF interaction, additional biochemical or genetic validation will substantially strengthen the conclusions. For example, targeted substitutions of key predicted interface residues followed by assessment of their effects on protein-protein interaction and biological function could provide direct experimental support for the proposed model.

4. Physiological relevance and cellular consequences of the 45°C heat-shock treatment: It is normally reasonable that bacteria occupying diverse environmental niches may encounter a broad range of stressful conditions, including high temperatures. However, I would like to re-emphasize my previous concern regarding the use of 45°C for 30 minutes in the HeLa cell infection experiments (Fig. 1F in the revised manuscript). Because Chlamydia is an obligate intracellular bacterium and these experiments were performed using infected HeLa cells, the relevant question is not only whether Chlamydia can respond to this temperature but also whether the host cells remain sufficiently viable and physiologically intact under these conditions. Although the authors explain that this treatment was intended to provide a brief and strong heat-shock stimulus, it remains unclear whether exposure to 45°C for 30 minutes causes substantial stress, loss of viability, or other physiological alterations in the host cells and/or intracellular Chlamydia.

Therefore, I believe that the physiological consequences of this treatment should be experimentally assessed or otherwise adequately justified. In particular, evidence regarding host-cell viability and, where feasible, Chlamydia viability or developmental integrity under these conditions would help establish whether the observed responses can be interpreted specifically as heat-shock regulation rather than as consequences of severe cellular damage.

5. Proposed essentiality of HrcA: The authors state that stable HrcA-deficient mutants have not been reported and suggest that loss of HrcA is therefore likely to be lethal. However, the absence of reported mutants does not necessarily establish that the gene is essential. Has disruption (such as knockdown) or deletion (knockout) of hrcA been experimentally attempted in Chlamydia, and, if so, what were the results? If direct experimental evidence for essentiality is not available, I suggest that the authors qualify this statement and clearly distinguish between experimentally demonstrated essentiality and a hypothesis based on the absence of reported viable mutants.

6. Mechanistic model for the role of HagF: The proposed role of HagF remains unclear to me (see Lines 85-92), and I would appreciate further clarification of the mechanistic model. As I understand the authors' hypothesis, HrcA appears to be temperature-sensitive or destabilized during heat stress. If this is correct, is the proposed model that HagF, which is strongly induced by heat shock, preferentially interacts with the destabilized form of HrcA? More specifically, do the authors propose that HagF binds to destabilized HrcA and thereby interferes with its association with CIRCE DNA? If so, it is unclear how this mechanism is reconciled with the expectation that destabilization of HrcA itself would reduce its DNA-binding activity and promote its dissociation from CIRCE. In other words, if heat-induced destabilization of HrcA is already sufficient to cause its dissociation from CIRCE, what additional regulatory function does HagF provide? Clarification of this point, together with experimental evidence supporting the proposed sequence of molecular events, would be important for understanding the central mechanistic model of the manuscript.

**********

**Part III – Minor Issues: Editorial and Data Presentation Modifications**

Please use this section for editorial suggestions as well as relatively minor modifications of existing data that would enhance clarity.

Reviewer #1: (No Response)

Reviewer #2: The authors have adequately addressed all of my previous concerns. They have provided additional clarifying data and/or modified their text to take into account the few limitations of the approaches they have utilized. As a result, their experimental results now more accurately match their conclusions.

Reviewer #3: The manuscript should clarify how hagF was initially identified as a new HrcA-regulated gene. In the Discussion (Lines 283-297), the authors first describe HagF as a sixth HrcA-regulated gene identified in this study, but subsequently state that “Kozusnik et al. reported stress-induced expression of ctl0271 (hagF) and its orthologs in Chlamydia-like organisms and proposed that these genes are HrcA targets.”. These statements create some ambiguity regarding what is novel in the present study. It would therefore be helpful for the authors to distinguish clearly between what was previously reported or proposed regarding hagF regulation and what new evidence provided in this manuscript establishes hagF as an HrcA-regulated gene.

**********

PLOS authors have the option to publish the peer review history of their article (what does this mean?). If published, this will include your full peer review and any attached files.

If you choose “no”, your identity will remain anonymous but your review may still be made public.

**Do you want your identity to be public for this peer review?**  For information about this choice, including consent withdrawal, please see our Privacy Policy.

Reviewer #1: No

Reviewer #2: No

Reviewer #3: No

[NOTE: If reviewer comments were submitted as an attachment file, they will be attached to this email and accessible via the submission site. Please log into your account, locate the manuscript record, and check for the action link "View Attachments". If this link does not appear, there are no attachment files.]

**Figure resubmission:**

While revising your submission, we strongly recommend that you use PLOS’s NAAS tool (https://ngplosjournals.pagemajik.ai/artanalysis) to test your figure files. NAAS can convert your figure files to the TIFF file type and meet basic requirements (such as print size, resolution), or provide you with a report on issues that do not meet our requirements and that NAAS cannot fix.

After uploading your figures to PLOS’s NAAS tool - https://ngplosjournals.pagemajik.ai/artanalysis, NAAS will process the files provided and display the results in the "Uploaded Files" section of the page as the processing is complete. If the uploaded figures meet our requirements (or NAAS is able to fix the files to meet our requirements), the figure will be marked as "fixed" above. If NAAS is unable to fix the files, a red "failed" label will appear above. When NAAS has confirmed that the figure files meet our requirements, please download the file via the download option, and include these NAAS processed figure files when submitting your revised manuscript.

**Reproducibility:**

To enhance the reproducibility of your results, we recommend that authors of applicable studies deposit laboratory protocols in protocols.io, where a protocol can be assigned its own identifier (DOI) such that it can be cited independently in the future. Additionally, PLOS ONE offers an option to publish peer-reviewed clinical study protocols. Read more information on sharing protocols at https://plos.org/protocols?utm_medium=editorial-email&utm_source=authorletters&utm_campaign=protocols

# Author comment: Author response to Decision Letter 2

**DOI:** 10.1371/journal.ppat.1014749.r004

24 Sep 2026

Attachment Submitted filename: Rebuttal R2.pdf

# Editor report: Decision Letter 2

**DOI:** 10.1371/journal.ppat.1014749.r005

**Authors:** Thomas Guillard, Leanid Laganenka

22 Oct 2026

Dear Dr. Fan,

We are pleased to inform that your manuscript, "A heat-induced HagF-HrcA regulatory loop promotes chlamydial fitness and infectious progeny yield under stress", has been editorially accepted for publication at PLOS Pathogens.

Before your manuscript can be formally accepted and sent to production, you will need to complete our formatting changes, which you will receive by email within a week. Please note that your manuscript will not be scheduled for publication until you have made the required changes.

IMPORTANT NOTES

(1) Copyediting and Proofreading: The corresponding author will receive a typeset proof for review, to ensure errors have not been introduced during production. Please review the PDF proof of your manuscript carefully, as this is the last chance to correct any errors. Please note that major changes, or those which affect the scientific understanding of the work, will likely cause delays to the publication date of your manuscript.

(2) Appropriate Figure Files: Please remove all name and figure # text from your figure files. Please also take this time to check that your figures are of high resolution, which will improve the readbility of your figures and help expedite your manuscript's publication. Please note that figures must have been originally created at 300dpi or higher. Do not manually increase the resolution of your files. For instructions on how to properly obtain high quality images, please review our Figure Guidelines, with examples at: http://journals.plos.org/plospathogens/s/figures.

We encourage you to use PACE (http://pace.apexcovantage.com/), a figure-checking tool which helps authors ensure all figures meet PLOS requirements so that figures can publish in the highest quality. PACE is a digital diagnostic and conversion tool for figure files. It will provide information about any failed check(s) and, if able, will automatically convert the figure into an acceptable file that passes quality checks.

To use PACE, you must first register as a user. Then, login and navigate to the UPLOAD tab, where you will find detailed instructions on how to use the tool.

(3) Striking Image: Please upload a striking still image to accompany your article if one is available (you can include a new image or an existing one from within your manuscript). Should your paper be accepted, this image will be considered for our monthly issue image and may also appear on our website to feature your article. Please upload this as a separate file, selecting "striking image" as the file type upon upload. Please also include a separate "Other" file with a caption, including credits and any potential copyright information. Please do not include the caption in the main article file. If your image is from someone other than yourself, please ensure that the artist has read and agreed to the terms and conditions of the Creative Commons Attribution License at http://journals.plos.org/plospathogens/s/content-license. Please note that PLOS cannot publish copyrighted images.

(4) Press Release or Related Media: If you or your institution will be preparing press materials for this manuscript, please inform our press team in advance at plospathogens@plos.org as soon as possible. We ask that you contact us within one week to plan ahead of our fast Production schedule. If you need to know your paper's publication date for related media purposes, you must coordinate with our press team, and your manuscript will remain under a strict press embargo until the publication date and time.

(5) Update your Profile Information: Now that your manuscript has been provisionally accepted, please log into Editorial Manager and update your profile, if needed. Go to http://www.editorialmanager.com/ppathogens, log in, and click on the "Update My Information" link at the top of the page. Please update your user information to ensure an efficient production and billing process.

(6) LaTeX users only: Our staff will ask you to upload a TEX file in addition to the PDF before the paper can be sent to typesetting, so please carefully review our Latex Guidelines http://journals.plos.org/plospathogens/s/latex in the meantime.

Thank you again for supporting Open Access publishing; we are looking forward to publishing your work in PLOS Pathogens.

Best regards,

Leanid Laganenka

Guest Editor

PLOS Pathogens

Thomas Guillard

Section Editor

PLOS Pathogens

Dominique Soldati-Favre

Editor-in-Chief

PLOS Pathogens

***********************************************************

# Editor report: Acceptance letter

**DOI:** 10.1371/journal.ppat.1014749.r006

**Authors:** Thomas Guillard, Leanid Laganenka

Dear Dr. Fan,

We are delighted to inform you that your manuscript, "A heat-induced HagF-HrcA regulatory loop promotes chlamydial fitness and infectious progeny yield under stress," has been formally accepted for publication in PLOS Pathogens.

We have now passed your article onto the PLOS Production Department who will complete the rest of the pre-publication process. All authors will receive a confirmation email upon publication.

Thank you again for supporting open-access publishing; we are looking forward to publishing your work in PLOS Pathogens.

Best regards,

Dominique Soldati-Favre

Editor-in-Chief

PLOS Pathogens
